# Ruthenium Half-Sandwich Type Complexes with Bidentate Monosaccharide Ligands Show Antineoplastic Activity in Ovarian Cancer Cell Models through Reactive Oxygen Species Production

**DOI:** 10.3390/ijms221910454

**Published:** 2021-09-28

**Authors:** István Kacsir, Adrienn Sipos, Gyula Ujlaki, Péter Buglyó, László Somsák, Péter Bai, Éva Bokor

**Affiliations:** 1 Department of Organic Chemistry, University of Debrecen, P.O. Box 400, H-4002 Debrecen, Hungary; kacsir.istvan@science.unideb.hu (I.K.); somsak.laszlo@science.unideb.hu (L.S.); 2 Doctoral School of Chemistry, University of Debrecen, P.O. Box 400, H-4002 Debrecen, Hungary; 3 Department of Medical Chemistry, Faculty of Medicine, University of Debrecen, H-4032 Debrecen, Hungary; siposadri@med.unideb.hu (A.S.); ujlaki.gyula@med.unideb.hu (G.U.); 4 Department of Inorganic & Analytical Chemistry, Faculty of Sciences and Technology, University of Debrecen, H-4032 Debrecen, Hungary; buglyo@science.unideb.hu; 5 NKFIH-DE Lendület Laboratory of Cellular Metabolism, H-4032 Debrecen, Hungary; 6 Research Center for Molecular Medicine, Faculty of Medicine, University of Debrecen, H-4032 Debrecen, Hungary

**Keywords:** ovarian cancer, ruthenium complex, half-sandwich, cooperative binding, reactive oxygen species production, glycosyl heterocycle, oxadiazole, triazole, rucaparib

## Abstract

Ruthenium complexes are developed as substitutes for platinum complexes to be used in the chemotherapy of hematological and gynecological malignancies, such as ovarian cancer. We synthesized and screened 14 ruthenium half-sandwich complexes with bidentate monosaccharide ligands in ovarian cancer cell models. Four complexes were cytostatic, but not cytotoxic on A2780 and ID8 cells. The IC_50_ values were in the low micromolar range (the best being 0.87 µM) and were similar to or lower than those of the clinically available platinum complexes. The active complexes were cytostatic in cell models of glioblastoma, breast cancer, and pancreatic adenocarcinoma, while they were not cytostatic on non-transformed human skin fibroblasts. The bioactive ruthenium complexes showed cooperative binding to yet unidentified cellular target(s), and their activity was dependent on reactive oxygen species production. Large hydrophobic protective groups on the hydroxyl groups of the sugar moiety were needed for biological activity. The cytostatic activity of the ruthenium complexes was dependent on reactive species production. Rucaparib, a PARP inhibitor, potentiated the effects of ruthenium complexes.

## 1. Introduction

**Figure 1:**
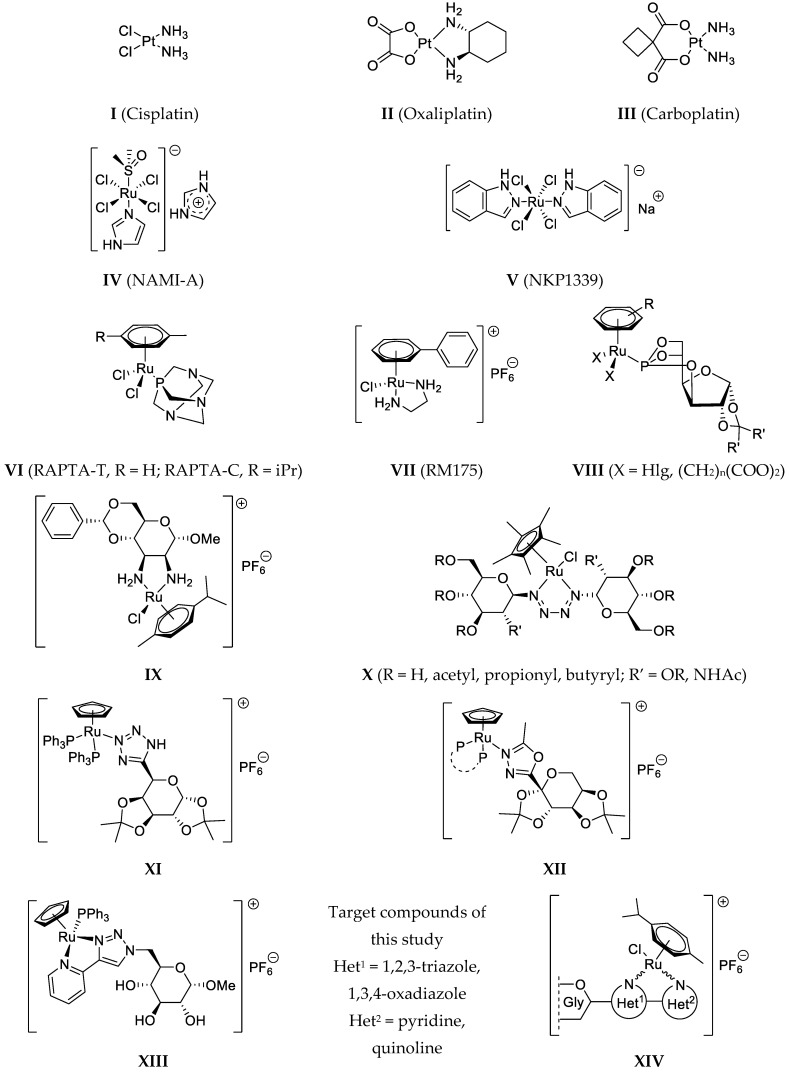
Selected platinum anticancer drugs and ruthenium complexes with proven anticancer potential.

Metal-based drugs used in cancer therapy include square planar platinum(II) complexes, e.g., cisplatin (Figure 1, **I**), oxaliplatin (**II**), and carboplatin (**III**), which are registered worldwide. Although Pt complexes are versatile tools of the trade, their applicability has shortcomings, such as the development of platinum resistance in tumors [1,2], ototoxicity [3], and nephrotoxicity [4,5], with the latter two being very characteristic for cisplatin.

Ruthenium complexes can be promising alternatives to platinum complexes due to their similar chemical characteristics in terms of ligand exchange [6]. In addition, Ru-complexes have enhanced delivery properties compared to Pt-based drugs. For example, Ru-based drugs have improved cellular entry under hypoxic conditions that characterize aggressively growing tumors [7,8], or they can be delivered by binding to transferrin [6]. Importantly, multiple studies have pointed out reduced toxicity for Ru-complexes as compared to Pt complexes in both cellular and animal models [9,10,11,12]. Both Pt- and Ru-based drugs can be targeted to enzymes or cellular compartments by coupling bait molecules such as biotin, nonsteroidal anti-inflammatory drugs, hormones, or carbohydrates to enhance their chemotherapeutic potential (thoroughly reviewed in [6]).

Among the Ru(III)- and Ru(II)-based complexes, a great number of derivatives have been tested as anticancer metallodrugs, and two of them, the imidazolium salt of tetrachlorido(dimethylsulfoxide)(imidazole)ruthenium(III) (Figure 1, **IV**) and sodium tetrachloridobis(indazole)ruthenium(III) (**V**), reached clinical trials [6,13,14,15,16]. The half-sandwich type Ru(II)-arene organometallics [17], due to a high structural variability, represent one of the most widely investigated compound classes in the development of new candidates of multitargeted metallodrugs [6,18]. The presence of an η^6^-arene or η^5^-arenyl residue in the coordination sphere contributes to the stabilization of the +2 oxidation state of the metal ion and to the maintenance of the hydrophilic/lipophilic balance of the whole molecule. The remaining three coordination sites of the Ru(II) ion are usually occupied by at least one leaving group and mono- or bidentate ligands, as can be seen in RAPTA-T and -C (**VI**) and RM175 (**VII**), respectively, which, among others, have become leads for Ru(II)-based complexes [15,17,19].

Due to the biological relevance of sugars, the incorporation of a carbohydrate-containing ligand into platinum group metal complexes in general [6,20] and into Ru(II)-arene/arenyl complexes in particular [15] seems a rational choice for further drug design. Thus, several functional features of carbohydrates, such as their contribution to different cellular phenomena (e.g., to cell–cell recognition and adhesion), their crucial role in cellular energy supply, and their binding capacities to carbohydrate-specific proteins (e.g., lectins, glucose transporters, and glycoenzymes), can be exploited to obtain new platinum group metal complexes with anticancer potential [15,20].

For several sugar-containing half-sandwich Ru(II) complexes, such as RAPTA analogs with glycofuranose-based phosphite ligands [15] (**VIII**) and Ru(II) complexes having 2,3-diamino-2,3-dideoxy-hexopyranoside [21] (**IX**) or 1,4-bis(β-d-glycopyranosyl)tetrazene-type *N,N*-chelating ligands [22] (**X**), the antiproliferative activity has already been justified. In addition, certain cyclopentadienyl-ruthenium(II) complexes with sugar-based heterocyclic mono- (**XI**, **XII**) or bidentate ligands (**XIII**) have also been shown to display low micromolar cytotoxic activity in human cervical carcinoma (HeLa) and colon cancer HCT116 cell lines [23,24,25].

Unlike organic drug molecules, the biological effects of metal complexes can be modulated by a wider array of parameters including size and charge of the species, hard–soft character of the metal ion, stability, inertness, and geometry of the complex, just to mention a few. In this regard, a more comprehensive study of Ru–sugar conjugate-based complexes, in which (A) the role of the metal chelating part of the ligand, (B) the basicity and binding strength of the coordinating donor atoms, and (C) the effect of the lipophilic/hydrophilic character of the complex, tuned by the presence/absence of various protecting groups at the sugar moiety, were explored, may provide a more detailed outlook of the structure–activity relationship (SAR) of these types of complexes.

As mentioned earlier, a rationale for the design and application of ruthenium complexes is to replace platinum compounds by ruthenium complexes in clinical settings. To the best of our knowledge, no real *C*- and *N*-glycopyranosyl heterocyclic ligands as potential bidentate chelators have so far been used to obtain Ru(II) arene/arenyl complexes. In an ongoing project focused on these types of sugar derivatives capable of forming either five- or six-membered chelates with Ru(II), herein we report on the synthesis and comprehensive characterization of a set of *C*- and *N*-glycopyranosyl azoles and their half-sandwich Ru-complexes (Figure 1, **XIV**). Since platinum complexes are widely used in the treatment of hematological and gynecological malignancies, the anticancer potential of the above ligands and their complexes were studied in comparison with Pt-complexes **I–III** against various human ovarian cancer cell lines.

## 2. Results

### 2.1. Chemistry

For the formation of the planned Ru(II) complexes, the sugar-based heterocyclic *N,N*-chelating ligands were prepared first.

The synthesis of 1-(β-d-glucopyranosyl)-4-hetaryl-1,2,3-triazoles was accomplished by the well-known copper(I)-catalyzed azide–alkyne cycloaddition [26,27] (CuAAc). Thus, the easily available 2,3,4,6-tetra-*O*-acetyl-β-d-glucopyranosyl azide [28,29] (**1**) was treated with ethynyl heterocycles **a** and **b** in the presence of bis-triphenylphosphano-copper(I)-butyrate [30,31] to give the expected *O*-peracetylated 1-(β-d-glucopyranosyl)-4-(pyridin-2-yl)- and -4-(quinolin-2-yl)-1,2,3-triazoles (**L-1a,b**) in high yields (Table 1). Removal of the *O*-acetyl protecting groups of **L-1a,b** was effected by the Zemplén method, resulting in the unprotected derivatives **L-3a,b** in good yields. *O*-Perbenzoylation of compound **L-3a** was then also carried out to give the 1-(2′,3′,4′,6′-tetra-*O*-benzoyl-β-d-glucopyranosyl)-4-(pyridin-2-yl)-1,2,3-triazole (**L-2a**) in excellent yield.

**Scheme 1:**
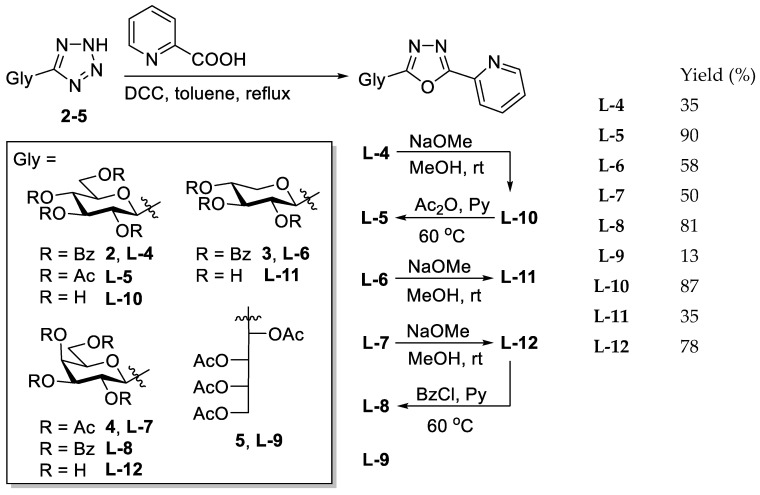
Synthesis of the monosaccharide-based 5-(pyridin-2-yl)-1,3,4-oxadiazoles.

The preparation of the sugar-based 5-(pyridin-2-yl)-1,3,4-oxadiazoles was carried out via the ring transformation of the corresponding 5-substituted tetrazoles following our previously reported procedure [32]. Thus, tetrazoles **2–5** were reacted with 2-picolinic acid in the presence of DCC under heating to furnish the desired 1,3,4-oxadiazoles **L-4**, **L-6**, **L-7**, and **L-9** in moderate yields (Scheme 1). The *O*-deprotected derivatives **L-10–L-12** were then obtained upon treatment of **L-4**, **L-6**, and **L-7**, respectively, with sodium methoxide in methanol. Under these conditions, the open-chain sugar derivative **L-9** did not furnish the expected *O*-deacetylated derivative. This might be due to the ring opening of the oxadiazole, as it was demonstrated for 2-(d-*arabino*-1,2,3,4-tetraacetoxybutyl)-5-methyl-1,3,4-oxadiazole [33]. Acetylation of **L-10** and benzoylation of **L-12** by using standard methods afforded the expected *O*-peracetylated 2-glucosyl-1,3,4-oxadiazole **L-5** and the *O*-perbenzoylated 2-galactosyl-1,3,4-oxadiazole **L-8**, respectively, in high yields.

To get the desired cationic half-sandwich Ru(II) complexes, the above heterocyclic monosaccharide derivatives were reacted with the commercially available dichloro(η^6^-*p*-cymene)ruthenium(II) dimer ([(η^6^-*p*-cym)RuCl_2_]_2_, **Ru-dimer**).

**Scheme 2:**
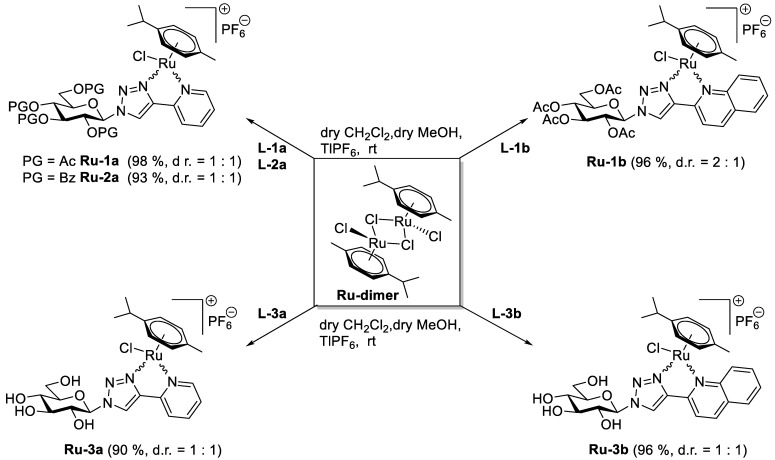
Synthesis of [(η^6^-*p*-cym)Ru^II^(N-N)Cl]PF_6_ complexes containing 1-(β-d-glucopyranosyl)-4-hetaryl-1,2,3-triazole ligands.

The complexation reactions of the **Ru-dimer** with an equimolar amount or a slight excess of the *O*-peracylated (**L-1** and **L-2**) and the *O*-unprotected (**L-3**) 1-(β-d-glucopyranosyl)-4-hetaryl-1,2,3-triazoles in the presence of the halide abstraction reagent TlPF_6_ in a CH_2_Cl_2_–MeOH solvent mixture were smoothly accomplished at room temperature to give the PF_6_^−^ salts of the expected [(η^6^-*p*-cym)Ru^II^(N-N)Cl]^+^ complexes **Ru-1–Ru-3** in excellent yields (Scheme 2). The complexes containing the *O*-peracylated glucosyl-1,2,3-triazole ligands (**Ru-1a,b** and **Ru-2a**) were stable and inert enough to be purified by column chromatography on silica gel, while the isolation of the highly polar complexes having the *O*-deprotected heterocyclic chelators (**Ru-3a,b**) could be effected by crystallization. Due to the formation of a new stereogenic center on the metal ion and the chiral nature of the glucose unit, diastereomers of the complexes were formed in each case, whose separation could be achieved neither by column chromatography nor by crystallization.

Complexation of the **Ru-dimer** with the monosaccharide-based 5-(pyridin-2-yl)-1,3,4-oxadiazoles (**L-4–L-12**) was also performed by applying the same procedure, resulting in diastereomeric mixtures of the corresponding [(η^6^-*p*-cym)Ru^II^(N-N)Cl]PF_6_ half-sandwich type complex molecules (Table 2). Column chromatographic purification for compounds **Ru-4–Ru-9** and recrystallization for **Ru-10–Ru-12** furnished the test molecules in moderate to high yields.

**Figure 2:**
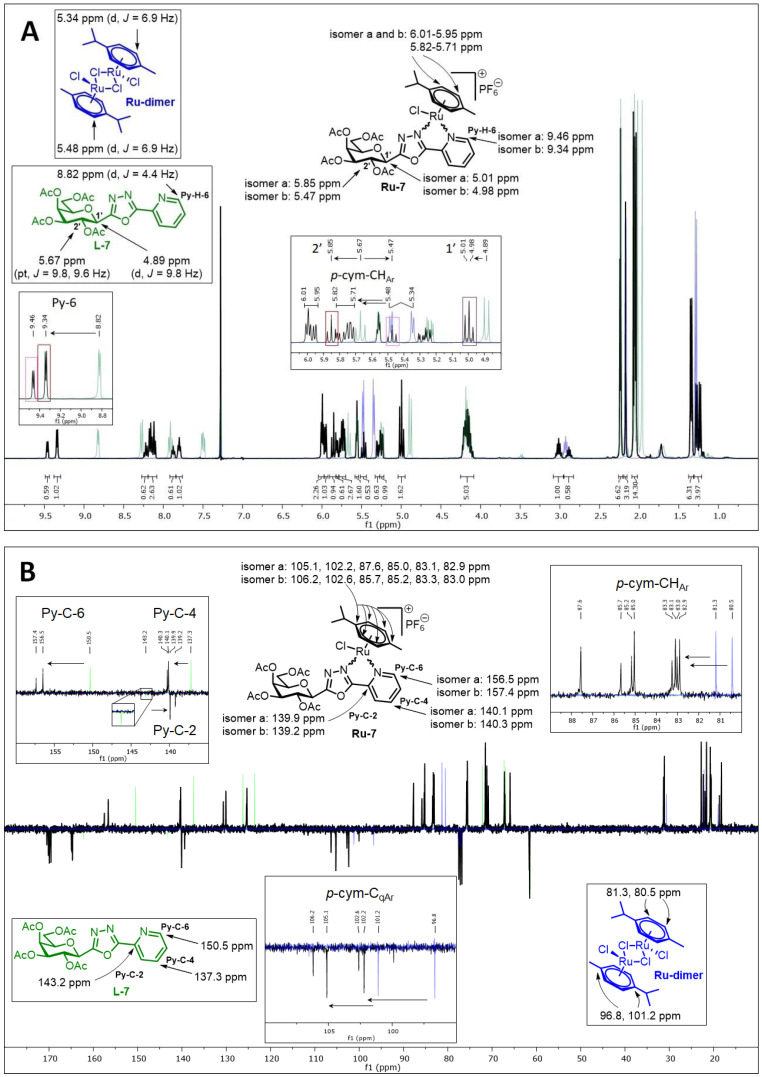
Superposition of the ^1^H-NMR (**A**) and ^13^C-NMR (**B**) spectra of **Ru-7** (black), ligand **L-7** (green), and **Ru-dimer** (blue) in CDCl_3_.

The formation of the complexes and the existence of the diastereomeric pairs were confirmed by ^1^H- and ^13^C-NMR spectroscopy in each case. As a representative, the superposition of the ^1^H- and ^13^C-NMR spectra of **Ru-7**, the free ligand **L-7**, and the **Ru-dimer**, respectively, are presented in Figure 2. Generally, the conversion of **Ru-dimer** into the corresponding [(η^6^-*p*-cym)Ru^II^(N-N)Cl]PF_6_ complexes (**Ru-1–Ru-12**) led to remarkable downfield shifts of the aromatic *p*-cymene signals in both the ^1^H- and the ^13^C-NMR spectra. No significant changes in the chemical shifts of the proton and carbon resonances of the sugar moiety were observed, except for those which were close to the coordination sphere. Thus, H-1′ protons of the sugar-based heterocyclic ligands usually displayed noticeable downfield shifts up to 0.05–0.27 ppm upon coordination. On the other hand, e.g., in the case of the *O*-acyl protected glycosyl-1,3,4-oxadiazole derivatives **L-4–L-8** the coordination resulted in either a downfield or an upfield shift of the H-2′ resonances depending on the diastereomers formed. The formation of the 5-membered chelates with the participation of heterocyclic aglycone parts was also shown by the change in the chemical shift of several heteroaromatic signals. For example, in case of the 2-pyridyl substituted derivatives, well-traceable and consistent changes were observed in the appearance of the proton and carbon signals of the pyridine ring. For example, the H-6 and C-6 signals of the pyridine ring showed downfield shifts up to 0.48–0.83 ppm (^1^H-NMR) and 5.7–7.8 ppm (^13^C-NMR), respectively, as a result of the coordination. A more detailed collection of these data can be found in Tables S1–S8 in the Supplementary Materials.

The aqueous stability of the complexes was also studied over time. As an example, the time dependence of the NMR spectra of **Ru-3a** is shown in Figure S1. The small shifts of signals attributable to the structural change of the complex can be observed; however, by adding KCl to the 2 day old equilibrated sample, the original signals could be recovered. This unambiguously proves that only the exchange of the coordinating chloride ion by a water molecule occurred in a reversible manner without affecting the dissociation of the five-membered *N*,*N*chelate.

**Scheme 3:**
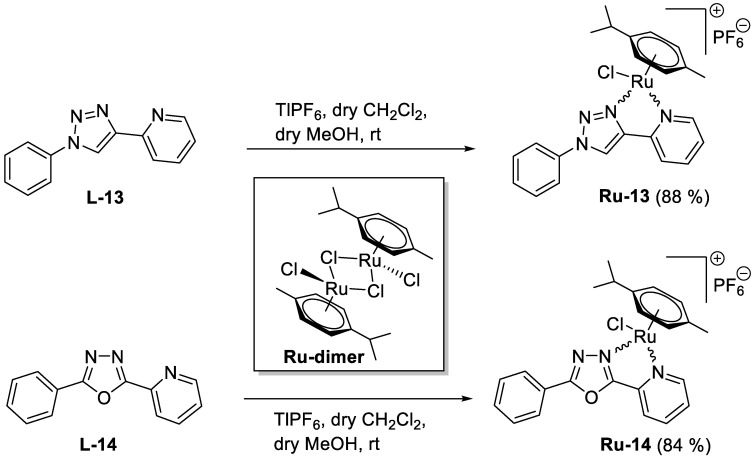
Synthesis of [(η^6^-*p*-cym)Ru^II^(N-N)Cl]PF_6_ complexes containing non-sugar-based azole ligands.

For comparative biological studies, two additional Ru(II) complexes containing non-sugar based ligands (Scheme 3, **Ru-13** and **Ru-14**) were also synthesized, starting from the **Ru-dimer** with 1-phenyl-4-(pyridine-2-yl)-1,2,3-triazole [34] (**L-13**) and 2-phenyl-5-(pyridine-2-yl)-1,3,4-oxadiazole [35,36] (**L-14**), respectively.

### 2.2. Identification of Ruthenium Compounds with Antineoplastic Properties

**Figure 3:**
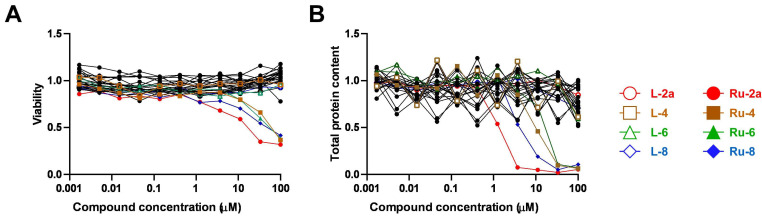
Screening of ruthenium complexes for cytostatic properties. (**A**,**B**) A total of 1 × 10^4^ (on panel A, MTT assays) or 3 × 10^3^ (on panel B, SRB assays) A2780 cells were plated on 96-well plates. (**A**) Cells were treated with the compounds indicated for 4 h, and then the MTT assay was performed. (**B**) Cells were treated with the compounds indicated for 48 h, and then the SRB assay was performed. Those molecules that were used in the subsequent studies are highlighted in color. All assays were performed in duplicate; all data represent three biological replicates. Values were normalized to vehicle-treated cells, where the absorbance of vehicle-treated cells was equal to 1.

We screened 14 ligands and their ruthenium complexes, bringing up the number of compounds to 28 in a concentration range of 100–0.0017 µM. The compounds were tested in an assay aiming to assess short-term toxicity (methylthiazolyldiphenyl-tetrazolium bromide (MTT) reduction assay, 4 h) and long-term cytostasis or cytotoxicity (sulforhodamine B (SRB) proliferation assay, 48 h) on A2780 human ovarian carcinoma cells. We found four complexes, **Ru-2a**, **Ru-4**, **Ru-6**, and **Ru-8**, possessing cytostatic properties in both long-term SRB and short-term MTT assays (Figure 3A,B). The uncomplexed ligands **L-2a**, **L-4**, **L-6**, and **L-8** had no biological activity (Figure 3A,B).

### 2.3. Ruthenium Compounds Have Similar Inhibitory Characteristics to Platinum Compounds

Next, we assessed the four active complexes, **Ru-2a**, **Ru-4**, **Ru-6**, and **Ru-8**, and the corresponding ligands, **L-2a**, **L-4**, **L-6**, and **L-8**, in detail. All compounds were tested on two ovarian cancer cell lines (A2780 and ID8) and on human primary skin fibroblasts (non-transformed, primary cells) in MTT and SRB assays. MTT assays were performed 4 h post treatment and indicated rapid toxicity of the compounds, while SRB assays were performed 2 days post treatment and represented long-term cytostasis or toxicity.

**Figure 4:**
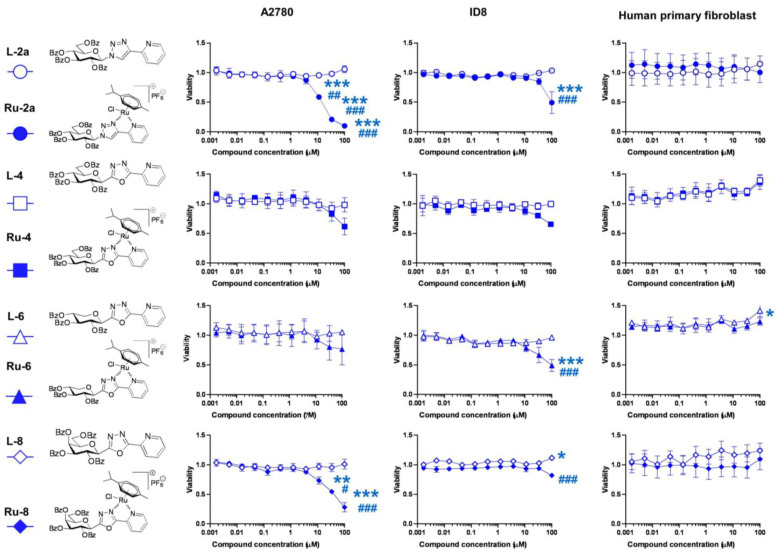
Assessment of the bioactive ruthenium complexes for acute toxicity. A total of 1 × 10^4^ A2780 cells, 8 × 10^3^ ID8 cells, and 6 × 10^3^ primary fibroblasts were plated on 96-well plates. Cells were treated with the compounds in the concentrations indicated for 4 h, and then the MTT assay was performed. Data are represented as the average ± SEM from three biological replicates; individual assays were performed in duplicate. Normality was tested; the **Ru-4**/**L4** fibroblast dataset had a normal distribution, **Ru-4**/**L4** ID8 dataset normality was achieved by logarithmic transformation, and the **Ru-4**/**L-4** A2780, **Ru-6**/**L-6** A2780, **Ru-8**/**L-8** A2780, **Ru-2a**/**L-2a** A2780, **Ru-6**/**L-6** ID8, **Ru-8**/**L-8** ID8, **Ru-2a**/**L-2a** ID8, **Ru-4**/**L-4** fibroblast, **Ru-8**/**L-8** fibroblast, and **Ru-2a**/**L-2a** fibroblast datasets were normalized using the Box–Cox normalization method. Statistical significance was determined using a two-way ANOVA test, and all measurement points were compared with each other; *, **, and *** indicate statistically significant differences between vehicle-treated (control) and free ligand/ruthenium complex-treated cells at *p* < 0.05, *p* < 0.01, and *p* < 0.001, respectively; ^#^, ^##^, and ^###^ indicate statistically significant differences between free ligand-treated and ruthenium complex-treated cells at *p* < 0.05, *p* < 0.01, and *p* < 0.001, respectively. Values were normalized to vehicle-treated cells, where the absorbance of vehicle-treated cells was equal to 1.

None of the free ligands exerted rapid toxicity on any of the cell lines in short-term MTT assays (Figure 4). Nevertheless, the application of **Ru-2a** and **Ru-8** above 10 µM concentration reduced the MTT signal in A2780 and ID8 cells. **Ru-6** reduced the MTT signal in ID8 cells at 100 µM, and a similar trend was observed on A2780 cells. Although it was not statistically significant, **Ru-4** led to a similar reduction in MTT signal at 100 µM in A2780 and ID8 cells. Complexes **Ru-2a**, **Ru-4**, **Ru-6**, and **Ru-8** had no effect on primary fibroblasts in MTT assays.

**Figure 5:**
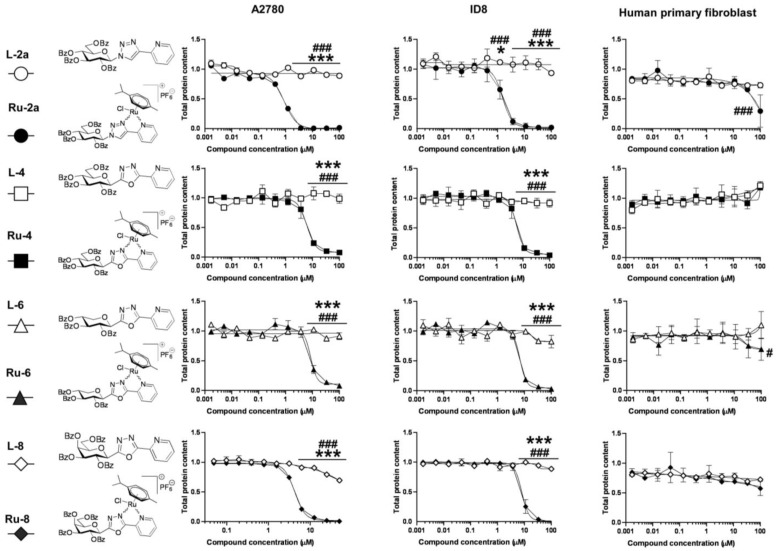
Assessment of the bioactive ruthenium complexes for cytostatic activity. A total of 3 × 10^3^ A2780 cells, 2 × 10^3^ ID8 cells, and 4 × 10^3^ primary fibroblasts were plated on 96-well plates. Cells were treated with the compounds in the concentrations indicated for 48 h, and then the SRB assay was performed. Data are represented as the average ± SEM from three biological replicates; individual assays were performed in duplicate. Normality was tested; the **Ru-4**/**L-4** A2780, **Ru-6**/**L-6** A2780, **Ru-8**/**L-8** A2780, **Ru-4**/**L-4** ID8, **Ru-6**/**L-6** ID8, **Ru-8**/**L-8** ID8, and **Ru-2a**/**L-2a** ID8 datasets had a normal distribution, **Ru-2a**/**L-2a** A2780 and **Ru-6**/**L-6** fibroblast dataset normality was achieved by logarithmic transformation, and the **Ru-4**/**L-4** fibroblast, **Ru-8**/**L-8** fibroblast, and **Ru-2a**/**L-2a** fibroblast datasets were normalized using the Box–Cox normalization method. Statistical significance was determined using a two-way ANOVA test, and all measurement points were compared with each other; * and *** indicate statistically significant differences between vehicle-treated (control) and free ligand/ruthenium complex-treated cells at *p* < 0.05, *p* < 0.01, and *p* < 0.001, respectively; ^#^ and ^###^ indicate statistically significant differences between free ligand-treated and ruthenium complex-treated cells at *p* < 0.05, *p* < 0.01, and *p* < 0.001, respectively. Nonlinear regression was performed on the data. Values were normalized to vehicle treated cells, where the absorbance of vehicle-treated cells was equal to 1.

Ruthenium complexes **Ru-2a**, **Ru-4**, **Ru-6**, and **Ru-8** were cytostatic, while none of the corresponding ligands (**L-2a**, **L-4**, **L-6**, and **L-8**) had cytostatic properties in long-term SRB assays on A2780 and ID8 cells (Figure 5). On primary human fibroblasts, the ligands, **L-2a**, **L-4**, **L-6**, and **L-8**, as well as two complexes, **Ru-4** and **Ru-8**, had no effect. In contrast, **Ru-2a** and **Ru-6** reduced the SRB signal at 100 µM on fibroblasts.

**Figure 6:**
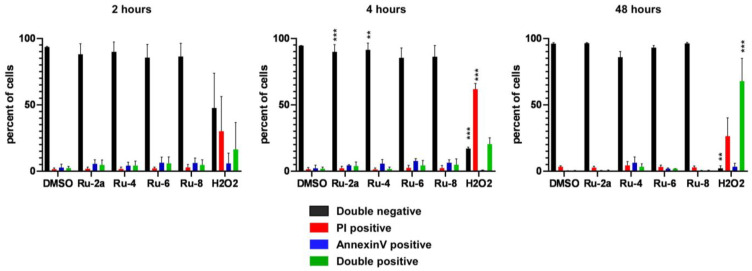
Ruthenium complexes do not induce cell death. A total of 1 × 10^6^ A2780 cells were plated on six-well plates and were treated with ruthenium complexes (**Ru-2a** at 1.3 µM, **Ru-4** at 6.2 µM, **Ru-6** at 8.5 µM, and **Ru-8** at 3.7 µM) and 300 µM hydrogen peroxide for 48 h. Cells were then stained with Annexin V and propidium iodide (PI), and cells were subjected to flow cytometry as described in Section 5. The percentages of cells in the quadrants are plotted. Data are presented as the average ± SEM from three biological replicates; individual assays were performed in duplicate. Normality was tested; on all datasets, Box–Cox normalization was performed to achieve a normal distribution. Statistical significance was determined using a two-way ANOVA test, and all measurement points were compared with each other; ** and *** indicate statistically significant differences between vehicle-treated (control) and treated cells (ruthenium complexes or 300 µM H_2_O_2_) corresponding to the same quadrant (e.g., vehicle-treated double-negative cells vs. **Ru-2a**-treated double-negative cells) at *p* < 0.01 and *p* < 0.001, respectively.

Cytostasis in the long term can be due to enhanced cell death. To exclude that possibility, we performed annexin V–propidium iodide (PI) double staining. Treating A2780 cells with **Ru-2a**, **Ru-4**, **Ru-6**, and **Ru-8** did not largely increase the proportions of PI-positive, annexin V-positive, and double-positive cells in contrast to hydrogen peroxide that was used as a positive control whether at 2 h, 4 h, or 48 h post treatment (Figure 6). The 4 h and 48 h timepoints were chosen to be the same as those used in the previous assays. The 2 h timepoint was found in our previous study to be optimal for the induction of apoptosis, marked by phosphatidylserine exposure [37,38,39].

**Figure 7:**
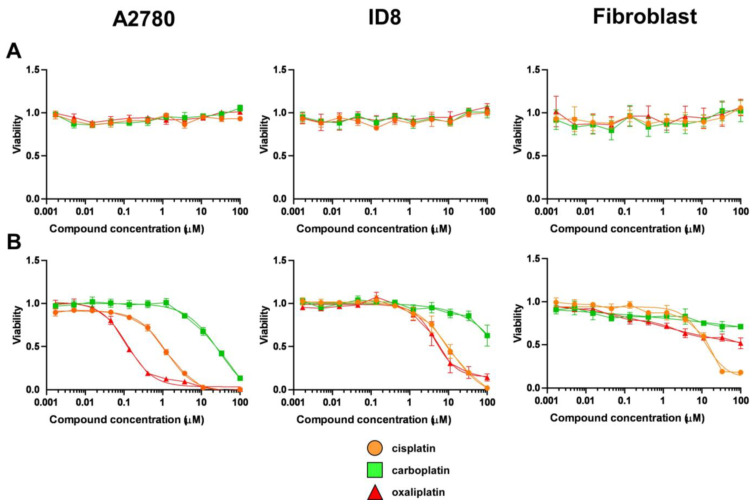
Determination of the cytotoxic and cytostatic activity of platinum compounds. (**A**) A total of 1 × 10^4^ A2780 cells, 8 × 10^3^ ID8 cells, and 6 × 10^3^ primary fibroblasts were plated on 96-well plates. Cells were treated with the compounds in the concentrations indicated for 4 h, and then the MTT assay was performed. (**B**) A total of 3 × 10^3^ A2780 cells, 2 × 10^3^ ID8 cells, and 4 × 10^3^ primary fibroblasts were plated on 96-well plates. Cells were treated with the compounds in the concentrations indicated for 48 h, and then the SRB assay was performed. Nonlinear regression was performed on the data. Values were normalized to vehicle-treated cells, where the absorbance of vehicle-treated cells was equal to 1.

Since ruthenium complexes are regarded as alternatives to platinum-centered drugs [6], we used the currently therapeutically available platinum-based drugs cisplatin (**I**), oxaliplatin (**II**), and carboplatin (**III**) as reference compounds and tested them on A2780 and ID8 cells, as well as on primary human fibroblasts. Platinum drugs had no effect in MTT assays (Figure 7A), while they were cytostatic on A2780 and ID8 cells in SRB assays (Figure 7B). Carboplatin and oxaliplatin had no effect on primary human fibroblasts in SRB assays, while cisplatin was cytostatic above 10 µM (Figure 7).

To compare the inhibitory properties of **Ru-2a**, **Ru-4**, **Ru-6**, and **Ru-8**, we performed a nonlinear regression of the long-term cytostatic curves (SRB curves) (Figure 5 and Figure 7B, Table 3). The IC_50_ values of **Ru-2a**, **Ru-4**, **Ru-6**, and **Ru-8** fell between 0.9 and 9 µM, and the A2780 cells were more sensitive to ruthenium complexes as compared to ID8 cells (Table 3). **Ru-2a** had the lowest IC_50_ value in A2780 and ID8 cells (0.9 and 1.5 µM, respectively). The IC_50_ values of the ruthenium complexes (0.9–9 µM) were comparable to or lower than those of the platinum compounds (0.1–28 µM) (Table 3).

In addition to the IC_50_ value, regression analysis yielded the Hill slope. The Hill slope is a readout, characterized by the slope of the curve, that provides information on the binding characteristics of a compound [40]. The Hill slope of platinum compounds on A2780 and ID8 cells was in the range of 0.6–1.6. In stark contrast to that, the Hill slope of the ruthenium complexes was in the range of 1.5–3.7 (Table 3). A higher Hill slope suggests higher cooperativity upon the binding of the ruthenium complexes to target biomolecules than in the case of platinum compounds.

### 2.4. The Biological Activity of Ruthenium Complexes Is Dependent on Reactive Oxygen Species Production

**Figure 8:**
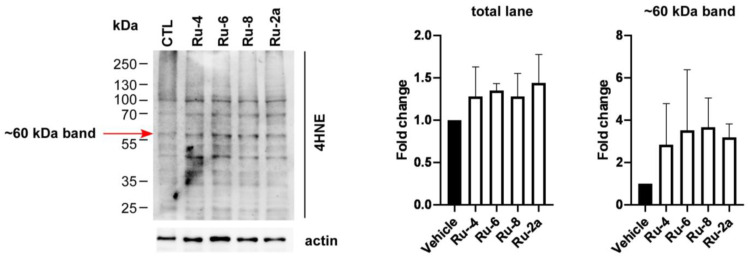
Ruthenium complexes induce reactive oxygen species production. A total of 1 × 10^6^ A2780 cells were plated on six-well plates. Cells were treated with **Ru-2a** at 1.3 µM, **Ru-4** at 6.2 µM, **Ru-6** at 8.5 µM, and **Ru-8** at 3.7 µM for 48 h. Then, the cells were harvested, and the protein lysate was prepared. Subsequently, 20 µg protein was separated by SDS-PAGE on an 8% gel, followed by Western blotting, and the blot was stained with 4HNE and actin antibodies. Densitometry values were normalized to the control. Data are represented as the average ± SEM from three biological replicates. Since there was no variance in the control group, we did not perform statistical analysis. Values were normalized to vehicle-treated cells, where the absorbance of vehicle-treated cells was equal to 1.

Currently available ruthenium complexes with antineoplastic activity have diverse modes of action involving (mitochondrial) reactive oxygen species production [9,15,41,42] and the induction of DNA damage [43,44]. We detected an increase in the expression of an oxidative stress marker, 4-hydroxy-nonenal (4HNE), at the level of the whole lane, as well as when a specific band was assessed, in A2780 cells treated with the active ruthenium complexes **Ru-2a**, **Ru-4**, **Ru-6**, and **Ru-8** corresponding to the IC_50_ values (Figure 8). 

**Figure 9:**
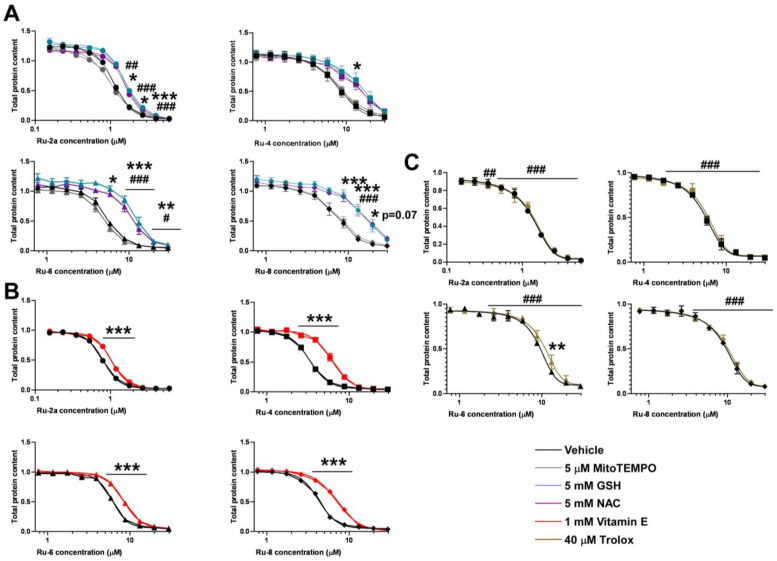
Reduced thiols and vitamin E can block the cytostatic effects of ruthenium complexes. (**A**) A total of 3 × 10^3^ A2780 cells were plated on 96-well plates. Cells were treated with the reduced thiol compounds in the concentrations indicated for 48 h, and then the SRB assay was performed. Data are presented as the average ± SEM from three biological replicates. The individual SRB assays were performed in duplicate. Normality was tested; the **Ru-4** dataset had a normal distribution, whereas **Ru-2a**, **Ru-6**, and **Ru-8** dataset normality was achieved by logarithmic transformation. Statistical significance was determined using a two-way ANOVA test, and all measurement points were compared with each other. (**B**) A total of 3 × 10^3^ A2780 cells were plated on 96-well plates. Cells were treated with the compounds in the concentrations indicated for 48 h, and then the SRB assay was performed. Data are presented as the average ± SEM from three biological replicates. The individual SRB assays were performed in duplicate. Normality was tested; the **Ru-4** and **Ru-8** datasets had a normal distribution, whereas **Ru-2a** and **Ru-6** dataset normality was achieved by logarithmic transformation. (**C**) A total of 3 × 10^3^ A2780 cells were plated on 96-well plates. Cells were treated with Trolox in the concentration indicated for 48 h, and then the SRB assay was performed. Data are presented as the average ± SEM from three biological replicates. The individual SRB assays were performed in duplicate. Normality was tested; the **Ru-2a**, **Ru-6**, and **Ru-8** datasets had a normal distribution, whereas **Ru-4** dataset normality was achieved by logarithmic transformation. Statistical significance was determined using a two-way ANOVA test, and all measurement points were compared with each other; *, **, and *** indicate statistically significant differences between vehicle-treated (control) and antioxidant (GSH, NAC, vitamin E, or Trolox)-treated cells at *p* < 0.05, *p* < 0.01, and *p* < 0.001, respectively; ^#^, ^##^, and ^###^ indicate statistically significant differences between vehicle-treated (control) and ruthenium-treated cells at *p* < 0.05, *p* < 0.01, and *p* < 0.001, respectively. Values were normalized to vehicle-treated cells, where the absorbance of vehicle-treated cells was equal to 1.

We assessed whether oxidative stress had a role in the cytostatic effects exerted by **Ru-2a**, **Ru-4**, **Ru-6**, and **Ru-8**. To that end, we tried to revert the cytostatic effects of ruthenium complexes using strong reductants such as reduced glutathione (GSH) and *N*-acetyl-cysteine (NAC). Furthermore, we also tested MitoTEMPO, a mitochondrially targeted antioxidant that can efficiently detoxify mitochondria-derived reactive oxygen species [45,46]. GSH and NAC cotreatment attenuated the cytostatic effects induced by **Ru-2a**, **Ru-4**, **Ru-6**, and **Ru-8** (Figure 9A) pointing to the causative role of reactive oxygen species production in cytostasis. Nevertheless, MitoTEMPO did not modulate the effects of **Ru-2a**, **Ru-4**, **Ru-6**, and **Ru-8** (Figure 9A) suggesting that mitochondria are not the source of the reactive oxygen species.

As thiols are soft Lewis bases and ruthenium is a soft Lewis acid, excess amounts of GSH or NAC can lead to the disassembly of ruthenium complexes. To provide evidence against this scenario, we applied another antioxidant, vitamin E, that does not have thiol groups. The application of vitamin E, similar to GSH and NAC, attenuated the cytostatic effects of the bioactive ruthenium complexes (Figure 9B), providing further evidence on the involvement of reactive oxygen species. Interestingly, treatment of the cells with Trolox, a derivative of vitamin E lacking the apolar phytyl chain, did not provide protection against ruthenium compounds (Figure 9C).

**Figure 10:**
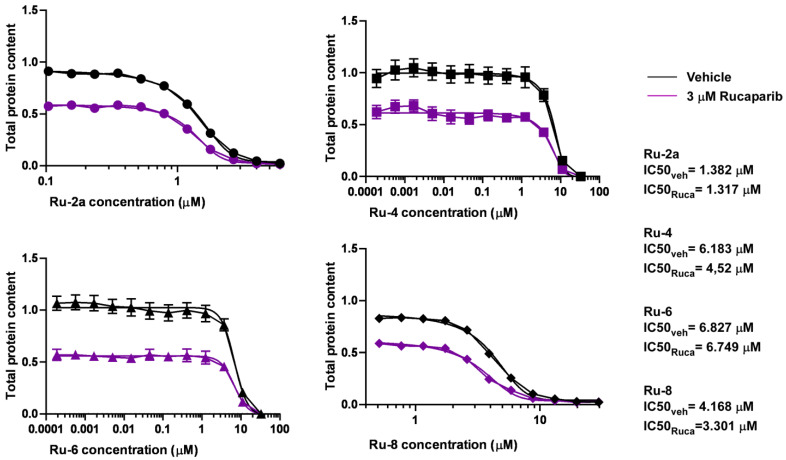
Rucaparib potentiates the cytostatic effects of ruthenium complexes. A total of 3 × 10^3^ A2780 cells were plated on 96-well plates. Cells were treated with rucaparib at 3 µM concentration for 48 h, and then the SRB assay was performed. Data are represented as the average ± SEM from four biological replicates. Nonlinear regression was performed to obtain the IC_50_ values. Values were normalized to vehicle-treated cells, where the absorbance of vehicle-treated cells was equal to 1.

Reactive oxygen species production leads to DNA damage and poly(ADP-ribose) polymerase (PARP) activation [47,48]. Certain ruthenium complexes were shown to potentiate the effects of PARP inhibitors [41,42,49,50]. We assessed whether **Ru-2a**, **Ru-4**, **Ru-6**, and **Ru-8** have similar properties by treating cells with 3 µM rucaparib, a clinically available potent PARP inhibitor. Rucaparib reduced cell proliferation in agreement with previous publications (e.g., [51]) (Figure 10). When we performed nonlinear regression analysis to assess the IC_50_ values, we found that rucaparib potentiated the effects of all ruthenium complexes marked by a decrease in the IC_50_ values in combination treatment (Figure 10). Nevertheless, it is of note that the potentiation was not as marked as the effects of antioxidants; hence, PARP-mediated effects probably have minor importance.

### 2.5. Bioactive Ruthenium Complexes Can Cause Cytostasis in Breast Carcinoma, Pancreatic Adenocarcinoma, and Glioblastoma Cell Lines

**Figure 11:**
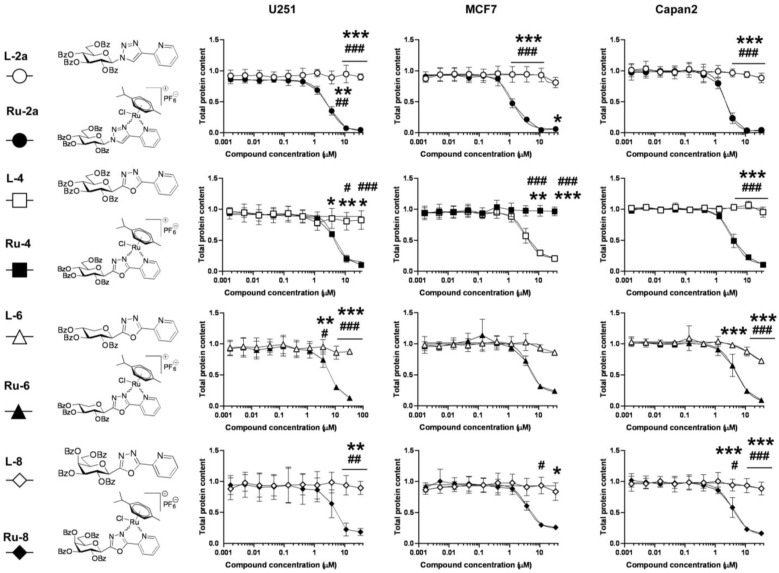
Assessment of the bioactive ruthenium complexes on models for glioblastoma, breast cancer, and pancreatic adenocarcinoma for cytostatic activity. A total of 2 × 10^3^ U251 cells (glioblastoma model), 3 × 10^3^ MCF7 cells (breast cancer), and 2 × 10^3^ Capan2 (pancreatic adenocarcinoma) were plated on 96-well plates. Cells were treated with the compounds in the concentrations indicated for 48 h, and then the SRB assay was performed. Data are presented as the average ± SEM from three biological replicates; individual assays were performed in duplicate. Normality was tested; the **Ru-4**/**L-4** U251, **Ru-8**/**L8** U251, **Ru-2a**/**L-2a** U251, **Ru-4**/**L-4** MCF7, **Ru-8**/**L8** MCF7, **Ru-4**/**L-4** Capan2, **Ru-8**/**L8** Capan2, and **Ru-2a**/**L-2a** Capan2 datasets had a normal distribution, **Ru-6**/**L-6** U251, **Ru-2a**/**L-2a** MCF7, and **Ru-6**/**L-6** Capan2 dataset normality was achieved by logarithmic transformation, and the **Ru-6**/**L-6** MCF7 dataset was normalized using the Box–Cox normalization method. Statistical significance was determined using a two-way ANOVA test, and all measurement points were compared with each other; *, **, and *** indicate statistically significant differences between vehicle-treated (control) and free ligand/ruthenium complex-treated cells at *p* < 0.05, *p* < 0.01, and *p* < 0.001, respectively; ^#^, ^##^, and ^###^ indicate statistically significant differences between free ligand-treated and ruthenium complex-treated cells at *p* < 0.05, *p* < 0.01, and *p* < 0.001, respectively. Nonlinear regression was performed on the data. Values were normalized to vehicle-treated cells, where the absorbance of vehicle-treated cells was equal to 1.

Prior studies [22,23,52,53] have suggested that sugar-based ruthenium complexes may be active on other cell lines and, therefore, potentially in other neoplastic diseases. We assessed breast cancer (modelled by MCF7 cells) as prior data [22] suggested the potential effectiveness of ruthenium complexes. Pancreatic adenocarcinoma (modelled by Capan2 cells) and glioblastoma (modelled by U251 cells), similar to ovarian cancer, are usually malign diseases where treatment options are limited [54,55,56]. All complexes exerted cytostatic effects on all cell lines in proliferation assays (SRB) (Figure 11). The IC_50_ values of the ruthenium complexes on U251, MCF7, and Capan2 were higher than on A2780 or ID8 cells (Table 3).

### 2.6. The Carbohydrate Moiety Is Important for the Bioactivity of the Ruthenium Complexes

**Figure 12:**
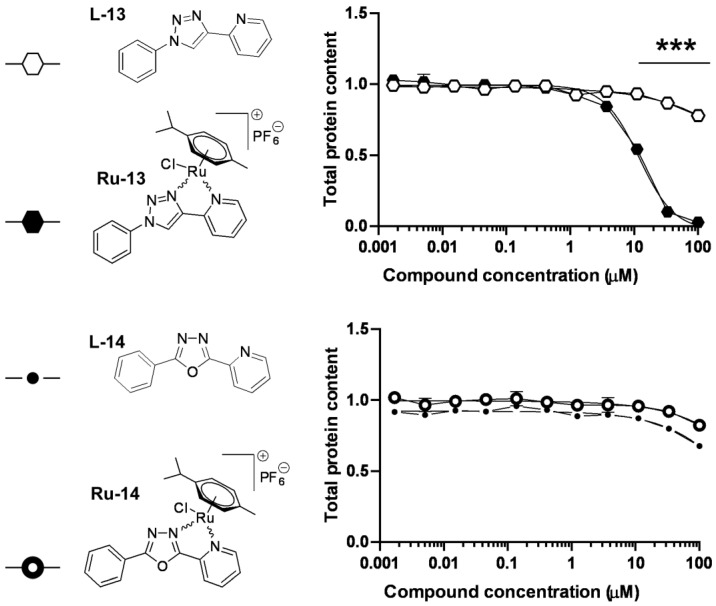
Assessment of non-sugar ruthenium complexes on A2780 cells for cytostatic activity. A total of 3 × 10^3^ A2780 cells were plated on 96-well plates. Cells were treated with the compounds in the concentrations indicated for 48 h, and then the SRB assay was performed. Data are represented as the average ± SEM, from three biological replicates; individual assays were performed in duplicate. Normality was tested; both datasets were normalized using the Box–Cox normalization method. Statistical significance was determined using a two-way ANOVA test, and all measurement points were compared with each other; *** indicates statistically significant differences between vehicle-treated (control) and free ligand/ruthenium complex-treated cells at *p* < 0.001. Nonlinear regression was performed on the data. Values were normalized to vehicle-treated cells, where the absorbance of vehicle-treated cells was equal to 1.

To assess the role of the monosaccharide moiety in the bioactive compounds, we synthesized and assessed two molecules where the monosaccharide unit was substituted with a phenyl group (**Ru-13**/**L-13** and **Ru-14**/**L-14**). Neither of the free ligands, **L-13** or **L-14**, had cytostatic activity on A2780 cells in SRB assays (Figure 12). The triazole-containing **Ru-13** was cytostatic on A2780 cells with an IC_50_ value of ~12 µM, which was higher by one order of magnitude than the IC_50_ value of the sugar-containing molecule **Ru-2a** (Figure 12, Table 3). Furthermore, the Hill coefficient of **Ru-13** was 1.5, suggesting no or little cooperativity. The oxadiazole-containing **Ru-14** had no cytostatic activity (Figure 12).

## 3. Discussion

In this study, we screened a set of carbohydrate-based half-sandwich type organoruthenium complexes. Four compounds were identified to display long-term cytostatic effects but little rapid toxicity on two different ovarian cancer cell lines. These compounds were not toxic toward primary human fibroblasts. The practical absence of toxicity on non-transformed fibroblasts aligns well with the low toxicity of other ruthenium complexes [9,10,11,12]. The activity of the compounds was dependent on the presence of ruthenium(II), as the ligands had no biological activity. The IC_50_ values of the active compounds were comparable or superior to the currently applied platinum compounds (cisplatin, oxaliplatin, and carboplatin) and other sugar-containing ruthenium complexes [22,23,52,53]. The identified Ru(II) compounds were active upon long-term application in SRB assays, similar to platinum compounds.

**Figure 13:**
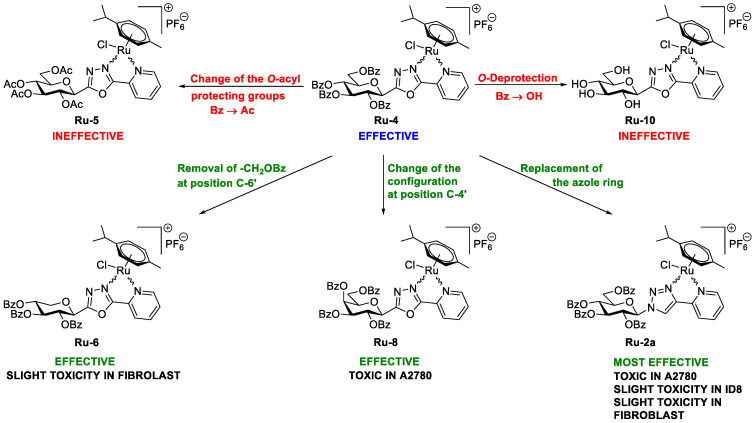
Structure–function relationship assessment of the active compounds.

We assessed the relationship between the biological activity and the structure of the active molecules. We compared all complexes to **Ru-4** that had considerable cytostatic potential in SRB assays on ovarian cancer cells but no activity on primary fibroblasts (Figure 13). An important structural feature that plays a key role in the biological activity of the molecules is the presence and size of the protecting groups on the hydroxyl groups of the carbohydrate moieties. All bioactive molecules (**Ru-2a**, **Ru-4**, **Ru-6**, and **Ru-8**) have *O*-benzoyl groups (Figure 13). Changing the *O*-benzoyl groups to smaller *O*-acetyl groups (**Ru-4** vs. **Ru-5**) or complete deprotection (**Ru-4** vs. **Ru-10**) abrogated the inhibitory activity. These findings are similar to the observations of Hamala and colleagues [22], who showed that the inhibitory activity of carbohydrate-based ruthenium complexes (Figure 1, **X**) was enhanced when the hydroxyl groups of the sugar units were protected by esters. Furthermore, increasing the length of the acyl chain improved the inhibitory efficacy (acetyl < propionyl < butyryl). In good agreement with that, the replacement of the protected monosaccharide in the molecule with a single phenyl group lowered the cytostatic capacity of the ruthenium complex (**Ru-13**) or fully abrogated it (**Ru-14**). This finding also corroborates the inevitable role of the sugar moiety in determining the biological activity. Apparently, increasing the lipophilic character of the compounds improves the cytostatic properties of sugar-based ruthenium complexes. This is underlined by the logD values in Table 3 and Table S9 showing a significant difference in lipophilicity in favor of the benzoylated derivatives. Hanif and colleagues [52] also had similar findings with RAPTA analogs as a function of the size of the arene cap and the acetal protecting group. It is tempting to speculate that these large, apolar protective groups could facilitate the membrane permeability of these compounds. In good agreement with that, Trolox, a derivative of vitamin E that lacks the long apolar phytyl chain, was ineffective in protecting cells against cytostasis induced by ruthenium compounds. This finding suggests oxidative stress in apolar, lipid-containing compartments.

Furthermore, we found two other structural features that impacted bioactivity: (A) modification of the carbohydrate moiety from glucose to xylose by a formal removal of the hydroxymethyl group at position 5 (**Ru-6**) or changing the configuration of the C-4 center (glucose to galactose as in **Ru-8**), and (B) replacement of the 1,3,4-oxadiazole ring by 1,2,3-triazole (**Ru-2a**). These changes increased the rapid toxicity of the molecules or rendered the molecules toxic on primary cells (Figure 13). In addition, a triazole ring in the molecule ensured better cytostatic properties than an oxadiazole ring (**Ru-13** vs. **Ru-14** and **Ru-2a** vs. **Ru-4**).

It was a surprising observation that the binding of the active compounds showed a high level of cooperativity, as suggested by the value of the Hill slope, being 2–3 for **Ru-2a**, **Ru-4**, **Ru-6**, and **Ru-8**. This suggests that the binding of a ruthenium complex facilitates the binding of the subsequent molecules. The Hill slope was also determined for the reference platinum-based drugs; however, these molecules did not show signs of cooperative binding as the Hill slope was ~1. Apparently, the binding and, probably, the mode of action of the ruthenium complexes identified in this study differ from those of the reference platinum compounds. In the study of Hanif et al. [52] assessing RAPTA analogs (general formula **VIII** in Figure 1), one compound was identified with a steep inhibitory curve, suggesting cooperative binding. This observation implicates that cooperative binding is not characteristic for all sugar-containing ruthenium complexes.

Our primary aim was to assess the applicability of ruthenium complexes in ovarian cancer, as these compounds were intended to be used as substitutes to platinum complexes [6]. Nevertheless, we provided evidence that the ruthenium complexes are also active in cell models of glioblastoma, breast cancer, and pancreatic adenocarcinoma, although the complexes showed preference toward ovarian cancer cells. Other sugar-containing ruthenium complexes were also active in ovarian cancer cell lines (A2780 [22,53], SK-OV-3 [22], CH1 [52,53]) further underlying the applicability of such compounds in ovarian cancer. Nevertheless, Ru–sugar complexes showed a cytostatic response in MDA-MD-231 breast cancer cells [22], colon cancer [52,53], non-small-cell lung cancer [52], and cervical carcinoma (HeLa) cells [23], suggesting a wider applicability of such compounds. 

Apparently, the mode of action of ruthenium complexes is pleiotropic and involves binding to polynucleotides [44] or the production of reactive oxygen species [9,15,41,42]. The actual mode of action or the dominant pathway to induce cytostasis differs for different classes of ruthenium complexes [15]. Carbohydrate–ruthenium complexes were shown to induce apoptosis that we did not detect for our compounds in concentrations corresponding to the IC_50_ values [22]. The active compounds identified in this study led to oxidative stress marked by increased 4HNE expression, a marker for lipid peroxidation. The functional role of reactive oxygen species production was confirmed by the application of GSH and NAC, two strong reductants, that reverted the cytostatic effects of the bioactive ruthenium complexes, as well as confirmed by using vitamin E. The source of reactive oxygen species was other than the mitochondria. Reactive oxygen species production was shown to be cytostatic in numerous carcinomas [9,15,41,42,57]. In addition to oxidative stress, we showed that the effects of ruthenium complexes is potentiated by a PARP inhibitor, rucaparib, similar to others [41,42,49,50]. Nevertheless, the level of potentiation was lower than the anticytostatic effects of antioxidants, pointing to a lower importance for PARP-mediated pathways. 

## 4. Conclusions

In this work, 14 novel half-sandwich Ru(II) complexes with real glycosyl azole type bidentate ligands were synthesized. To this end, 4-(pyridin-2-yl)- and 4-(quinolin-2-yl)-1-(β-d-glucopyranosyl)-1,2,3-triazoles (*N*-glycosyl derivatives) and 2-(β-d-glycosyl)-5-(pyridin-2-yl)-1,3,4-oxadiazoles (*C*-glycosyl derivatives) were prepared by 1,3-dipolar cycloaddition reactions. Treatment of these *N,N*-chelators with dichloro(η^6^-*p*-cymene)ruthenium(II) dimer ([(η^6^-*p*-cym)RuCl_2_]_2_) in the presence of TlPF_6_ yielded the expected Ru(II)-centered complexes with the general formula [(η^6^-*p*-cym)Ru^II^(N-N)Cl]PF_6_ as mixtures of diastereomers. 

In the biological study of the complexes in ovarian adenocarcinoma cells, antineoplastic properties characterized by little acute toxicity but long-term cytostasis were identified. The bioactive ruthenium complexes had micromolar IC_50_ values on A2780 and ID8 cells, while they had little or no activity on primary, non-transformed human fibroblasts, highlighting the low toxicity and selectivity of these compounds toward transformed cancer cells. The presence of the sugar moiety equipped with large hydrophobic protective groups on the hydroxyl groups of was found to be crucial for the biological activity. The bioactive ruthenium complexes, identified herein, showed cooperative binding to yet unidentified cellular target(s) and induced oxidative stress that was essential for their cytostatic activity.

## 5. Materials and Methods

### 5.1. Synthesis

#### 5.1.1. General Methods

Optical rotations were measured on a Jasco P-2000 polarimeter (Jasco, Easton, MD, USA) at r.t., and the data referred to the average of three parallel measurements. NMR spectra were recorded with DRX360 (360/90 MHz for ^1^H/^13^C) or DRX400 (400/100 MHz for ^1^H/^13^C) spectrometers (Bruker, Karlsruhe, Germany). Chemical shifts are referenced to Me_4_Si (^1^H-NMR) or to the residual solvent signals (^13^C-NMR). Assigments of the proton and carbon signals of the new compounds were based on COSY and HSQC correlations. MS spectra were obtained using a Bruker maXis II (ESI-HRMS) spectrometer. TLC analysis were carried out by using DC Kieselgel 60 F_254_ plates (Sigma-Aldrich, Saint Louis, MO, USA), and the spots were visualized under UV light and by gentle heating. For column chromatographic purification, Kieselgel 60 (Molar Chemicals, Halásztelek, Hungary, particle size 0.063–0.2 mm) silica gel was applied. Anhydrous solvents were obtained by using standard distillation procedures. Anhydrous CH_2_Cl_2_, CHCl_3_, and toluene were produced by distillation from P_4_O_10_ and then stored over 4 Å molecular sieves (CH_2_Cl_2_, CHCl_3_) or sodium wires (toluene). MeOH was dried by distillation over Mg turnings and iodine. The 2-ethynylpyridine (TCI Chemicals, Zwijndrecht, The Netherlands) and the dichloro(η^6^-*p*-cymene)ruthenium(II) dimer (**Ru-dimer**, Strem Chemicals, Newburyport, MA, USA) were purchased from the indicated suppliers. The 2,3,4,6-tetra-*O*-acetyl-β-d-glucopyranosyl-azide [28,29] (**1**), the 5-(2′,3′,4′,6′-tetra-*O*-benzoyl-β-d-glucopyranosyl)-tetrazole [58,59] (**2**), the 5-(2′,3′,4′-tri-*O*-benzoyl-β-d-xylopyranosyl)-tetrazole [60] (**3**), the 5-(2′,3′,4′,6′-tetra-*O*-acetyl-β-d-galactopyranosyl)-tetrazole [61] (**4**), the 2-(l-*arabino*-1′,2′,3′,4′-tetraacetoxybutyl)-tetrazole [33] (**5**), the 2-ethynylquinoline [62], 1-phenyl-4-(pyridine-2-yl)-1,2,3-triazole [34] (**L-13**), and the 2-phenyl-5-(pyridine-2-yl)-1,3,4-oxadiazole [35,36] (**L-14**) were synthesized according to procedures in the literature.

#### 5.1.2. General Procedure I for the Preparation of 1-(2′,3′,4′,6′-Tetra-*O*-acetyl-β-d-glucopyranosyl)-4-hetaryl-1,2,3-triazoles

To a solution of 1-(2,3,4,6-tetra-*O*-acetyl-β-d-glucopyranosyl)-azide [28,29] (**1**) in CH_2_Cl_2_ (1 mL/50 mg azide), the appropriate 2-ethynylated heterocycle (1 eq.) and the C_3_H_7_COOCu(PPh_3_)_2_ catalyst [30] (3 mol.%) were added. The reaction mixture was stirred at r.t. for 1 day, and the completion of the reaction was judged by TLC (1:1 EtOAc–hexane). The solvent was then removed under diminished pressure, and the residue was purified by column chromatography.

#### 5.1.3. General Procedure II for the Synthesis of *O*-Peracylated 2-glycosyl-5-(pyridin-2-yl)-1,3,4-oxadiazoles

The corresponding *O*-peracylated 5-glycosyl-tetrazole (**2–5**) was dissolved in dry toluene (1 mL/100 mg substrate), and then 2-picolinic acid (2 eq.) and DCC (2 eq.) were added. The reaction mixture was stirred at boiling temperature until the TLC showed total consumption of the tetrazole. The insoluble materials were filtered off and washed with CH_2_Cl_2_, and the filtrate was evaporated under reduced pressure. The crude product was purified by column chromatography and crystallisation.

#### 5.1.4. General Procedure III for the Zemplén Deacylation

An *O*-acyl protected glycosyl-azole was dissolved in a 1:1 mixture of dry MeOH and dry CHCl_3_ (1 mL/25 mg substrate), and a few drops of a ~1 M solution of NaOMe in MeOH was added (pH = 8–9). The reaction mixture was left at r.t. until the TLC indicated total conversion of the starting material. The neutralization of the solution was carried out by the addition of a cation exchange resin (Amberlyst 15, H^+^ form). The resin was then filtered off, and the solution was evaporated under reduced pressure. The crude product was purified by column chromatography or crystallization.

#### 5.1.5. General Procedure IV for the Formation of the [(η^6^-p-cym)Ru^II^(N-N))Cl]PF_6_ Type Complexes Containing *O*-peracylated Glycosyl Azole Ligands

To a solution of ([(η^6^-*p*-cym)RuCl_2_]_2_) dimer (**Ru-dimer**) in CH_2_Cl_2_ (1 mL/10 mg dimer), the corresponding *O*-peracylated glycosyl azole (2–2.3 eq.) and TlPF_6_ (2 eq.) were added. Under stirring, 1 mL of methanol was added to this reaction mixture in order to accelerate the precipitation of the TlCl. The initial red solution turned to yellow, indicating the formation of the [(η^6^-*p*-cym)Ru^II^(N-N)Cl]PF_6_ type complex. The reaction mixture was then stirred at r.t. for an additional hour, and the total disappearance of the **Ru-dimer** was judged by TLC (9:1 CHCl_3_-MeOH). After completion of the complexation reaction, the precipitated TlCl was filtered off, and the solution was evaporated in vacuo. The crude complex was purified by column chromatography or crystallization.

#### 5.1.6. General Procedure V for the Formation of the [(η^6^-p-cym)Ru^II^(N-N)Cl]PF_6_ Type Complexes Containing Unprotected Glycosyl Azole Ligands

In a solution of ([(η^6^-*p*-cym)RuCl_2_]_2_) dimer (**Ru-dimer**) in CH_2_Cl_2_ (1 mL/10 mg dimer), the corresponding unprotected glycosyl azole (2 eq.) and TlPF_6_ (2 eq.) were suspended. Under stirring, 1 mL of methanol was added to this reaction mixture in order to dissolve the heterocyclic sugar derivative and accelerate the precipitation of the TlCl. The initial red solution turned to yellow, indicating the formation of the [(η^6^-*p*-cym)Ru^II^(N-N)Cl]PF_6_ type complex. The reaction mixture was then stirred at r.t. for an additional hour, and the total disappearance of the **Ru-dimer** was judged by TLC (9:1 CHCl_3_-MeOH). After completion of the complexation reaction, the precipitated TlCl was filtered off, and the solution was evaporated in vacuo. The crude complex was purified by crystallization.

#### 5.1.7. 1-(2′,3′,4′,6′-Tetra-*O*-acetyl-β-d-glucopyranosyl)-4-(pyridin-2-yl)-1,2,3-triazole (**L-1a**) 

Prepared from azide [28,29] **1** (100 mg, 0.27 mmol) and 2-ethynylpyridine (28 µL, 0.27 mmol) according to general procedure I. Purified by column chromatography (1:1 → 2:1 EtOAc–hexane) to give 121 mg white amorphous solid (95%). R_f_ = 0.18 (1:1 EtOAc–hexane); [α]_D_ = −55 (c 0.21, CHCl_3_). ^1^H-NMR (360 MHz, CDCl_3_) δ (ppm): 8.61 (1H, d, *J* = 4.8 Hz, Py-H-6), 8.42 (1H, s, Tria-H-5), 8.15 (1H, d, *J* = 7.8 Hz, Py-H-3), 7.78 (1H, dt, *J* = 7.8, 1.6 Hz, Py-H-4), 7.25 (1H, m, Py-H-5), 5.94 (1H, d, *J* = 8.8 Hz, H-1′), 5.51 (1H, pt, *J* = 9.5, 9.5 Hz, H-2′), 5.46 (1H, pt, *J* = 9.5, 9.5 Hz, H-3′), 5.28 (1H, pt, *J* = 9.5, 9.5 Hz, H-4′), 4.32 (1H, dd, *J* = 12.6, 4.8 Hz, H-6′a), 4.17 (1H, dd, *J* = 12.6, 1.7 Hz, H-6′b), 4.04 (1H, ddd, *J* = 9.5, 4.8, 1.7 Hz, H-5′), 2.10, 2.08, 2.05, 1.90 (4 × 3H, 4 s, 4 × CH_3_); ^13^C-NMR (90 MHz, CDCl_3_) δ (ppm): 170.6, 170.1, 169.4, 168.9 (4 × C=O), 149.8, 149.2 (Tria-C-4, Py-C-2), 149.7 (Py-C-6), 137.0 (Py-C-4), 123.3 (Py-C-5), 120.5 (Py-C-3), 120.7 (Tria-C-5), 86.0 (C-1′), 75.3 (C-5′), 72.8 (C-3′), 70.7 (C-2′), 67.8 (C-4′), 61.7 (C-6′), 20.8, 20.7 (2), 20.3 (4 × CH_3_). The ^1^H and ^13^C NMR data are in good agreement with the reported ones [63]. ESI-HRMS positive mode (*m*/*z*): calculated for C_21_H_25_N_4_O_9_^+^ [M + H]^+^ 477.1616; C_21_H_24_N_4_NaO_9_^+^ [M + Na]^+^ 499.1435. Found: [M + H]^+^ 477.1614; [M + Na]^+^ 499.1432.

#### 5.1.8. 1-(2′,3′,4′,6′-Tetra-*O*-acetyl-β-d-glucopyranosyl)-4-(quinolin-2-yl)-1,2,3-triazole (**L-1b**) 

Prepared from azide [28,29] **1** (1.00 g, 2.68 mmol) and 2-ethynylquinoline [62] (0.41 g, 2.68 mmol) according to general procedure I. Purified by column chromatography (1:1 EtOAc–hexane) to yield 1.20 g white amorphous solid (85%). R_f_ = 0.23 (1:1 EtOAc–hexane); [α]_D_ = −90 (c 0.20, CHCl_3_). ^1^H-NMR (360 MHz, CDCl_3_) δ (ppm): 8.63 (1H, s, Tria-H-5), 8.31 (1H, d, *J* = 8.6 Hz, Qu-H-3), 8.24 (1H, d, *J* = 8.6 Hz, Qu-H-4), 8.08 (1H, d, *J* = 8.5 Hz, Qu-H-5 or Qu-H-8), 7.82 (1H, d, *J* = 8.0 Hz, Qu-H-5 or Qu-H-8), 7.72 (1H, pt, *J* = 7.9, 7.6 Hz, Qu-H-6 or Qu-H-7), 7.53 (1H, pt, *J* = 7.5, 7.4 Hz, Qu-H-6 or Qu-H-7), 5.99 (1H, d, *J* = 9.2 Hz, H-1′), 5.59 (1H, pt, *J* = 9.4, 9.2 Hz, H-2′), 5.48 (1H, pt, *J* = 9.5, 9.4 Hz, H-3′), 5.31 (1H, pt, *J* = 9.7, 9.5 Hz, H-4′), 4.35 (1H, dd, *J* = 12.6, 4.8 Hz, H-6′a), 4.18 (1H, dd, *J* = 12.6, 2.1 Hz, H-6′b), 4.07 (1H, ddd, *J* = 9.7, 4.8, 2.1 Hz, H-5′), 2.11, 2.09, 2.05, 1.91 (4 × 3H, 4 s, 4 × CH_3_); ^13^C-NMR (90 MHz, CDCl_3_) δ (ppm): 170.6, 170.1, 169.4, 169.0 (4 × C=O), 149.8, 149.4, 148.2 (Tria-C-4, Qu-C-2, Qu-C-8a), 137.0 (Qu-C-4), 129.9 (Qu-C-6 or Qu-C-7), 129.3 (Qu-C-5 or Qu-C-8), 128.0 (Qu-C-4a), 127.8 (Qu-C-5 or Qu-C-8), 126.6 (Qu-C-6 or Qu-C-7), 121.4 (Tria-C-5), 118.7 (Qu-C-3), 86.0 (C-1′), 75.3 (C-5′), 72.9 (C-3′), 70.7 (C-2′), 67.8 (C-4′), 61.7 (C-6′), 20.8, 20.6 (2), 20.3 (4 × CH_3_). The ^1^H and ^13^C NMR data are in good agreement with the reported ones [64]. ESI-HRMS positive mode (*m*/*z*): calculated for C_25_H_27_N_4_O_9_^+^ [M + H]^+^ 527.1773; C_25_H_26_N_4_NaO_9_^+^ [M + Na]^+^ 549.1592. Found: [M + H]^+^ 527.1773; [M + Na]^+^ 549.1593.

#### 5.1.9. 1-(2′,3′,4′,6′-Tetra-*O*-benzoyl-β-d-glucopyranosyl)-4-(pyridin-2-yl)-1,2,3-triazole (**L-2a**)

The 1-(β-d-glucopyranosyl)-4-(pyridin-2-yl)-1,2,3-triazole (**L-3a**, 20.0 mg, 0.065 mmol) was suspended in dry pyridine (0.5 mL), and benzoyl chloride (36 µL, 0.310 mmol) was added. The reaction mixture was stirred at 60 °C until the TLC (3:2 EtOAc–hexane) showed complete disappearance of the starting material (1 h). The solvent was removed under diminished pressure. The residue was dissolved in CH_2_Cl_2_ (20 mL) and extracted with sat. aq. NaHCO_3_ (10 mL) and then with water (10 mL). The organic phase was dried over MgSO_4_, filtered, and evaporated. Purification by column chromatography (3:2 EtOAc–hexane) yielded 41 mg of white amorphous solid (87%). R_f_ = 0.30 (3:2 EtOAc–hexane); [α]_D_ = −75 (c 0.20, CHCl_3_). ^1^H-NMR (400 MHz, CDCl_3_) δ (ppm): 8.61 (2H, 1 signal, Tria-H-5, Py-H-6), 8.17–7.21 (23H, m, Ar, Py-H-3–Py-H-5), 6.34 (1H, d, *J* = 9.2 Hz, H-1′), 6.16 (1H, pt, *J* = 9.6, 9.5 Hz, H-3′) 6.07 (1H, pt, *J* = 9.5, 9.2 Hz, H-2′), 5.90 (1H, pt, *J* = 9.6, 9.6 Hz, H-4′), 4.69–4.49 (3H, m, H-5′, H-6′a, H-6′b); ^13^C-NMR (100 MHz, CDCl_3_) δ (ppm): 166.2, 165.8, 165.2, 164.7 (4 × C=O), 149.7, 149.0 (Tria-C-4, Py-C-2), 149.6 (Py-C-6), 137.1 (Py-C-4), 133.8, 133.7, 133.6, 133.5, 133.4, 130.2–128.2 (Ar), 123.3 (Py-C-5), 121.0 (Tria-C-5), 120.6 (Py-C-3), 86.4 (C-1′), 75.7 (C-5′), 73.2 (C-3′), 71.3 (C-2′), 69.0 (C-4′), 62.8 (C-6′). ESI-HRMS positive mode (*m*/*z*): calculated for C_41_H_32_N_4_NaO_9_^+^ [M + Na]^+^ 747.2061. Found: 747.2041.

#### 5.1.10. 1-(β-d-Glucopyranosyl)-4-(pyridin-2-yl)-1,2,3-triazole (**L-3a**) 

Prepared from compound **L-1a** (0.81 g, 1.70 mmol) according to general procedure III. Purified by column chromatography (7:2 CHCl_3_–MeOH) to give 0.40 g white amorphous solid (76%). R_f_ = 0.26 (7:2 CHCl_3_–MeOH); [α]_D_ = −12 (c 0.20, MeOH). ^1^H-NMR (400 MHz, CD_3_OD) δ (ppm): 8.63 (1H, s, Tria-H-5), 8.59 (1H, d, *J* = 4.3 Hz, Py-H-6), 8.09 (1H, d, *J* = 7.9 Hz, Py-H-3), 7.92 (1H, pt, *J* = 7.9, 7.8 Hz, Py-H-4), 7.38 (1H, m, Py-H-5), 5.70 (1H, d, *J* = 9.2 Hz, H-1′), 3.96 (1H, pt, *J* = 9.1, 9.0 Hz, H-2′), 3.90 (1H, dd, *J* = 12.2, 1.3 Hz, H-6′a), 3.74 (1H, dd, *J* = 12.2, 5.3 Hz, H-6′b), 3.64–3.58 (2H, m, H-3′ or H-4′, H-5′), 3.54 (1H, pt, *J* = 9.2, 9.1 Hz, H-3′ or H-4′); ^13^C-NMR (90 MHz, CD_3_OD) δ (ppm): 150.9, 148.6 (Tria-C-4, Py-C-2), 150.5 (Py-C-6), 138.9 (Py-C-4), 124.6 (Py-C-5), 123.4 (Tria-C-5), 121.7 (Py-C-3), 89.8 (C-1′), 81.2 (C-5′), 78.5 (C-3′ or C-4′), 74.1 (C-2′), 70.9 (C-3′ or C-4′), 62.4 (C-6′). The ^1^H and ^13^C NMR data are in good agreement with the reported ones [65]. ESI-HRMS positive mode (*m*/*z*): calculated for C_13_H_16_N_4_NaO_5_^+^ [M + Na]^+^ 331.1013; C_26_H_32_N_8_NaO_10_^+^ [2M + Na]^+^ 639.2134. Found: [M + Na]^+^ 331.1012; [2M + Na]^+^ 639.2135.

#### 5.1.11. 1-(β-d-Glucopyranosyl)-4-(quinolin-2-yl)-1,2,3-triazole (**L-3b**)

Prepared from compound **L-1b** (500 mg, 0.95 mmol) according to general procedure III. The crude product was recrystallized from MeOH to yield 290 mg of white amorphous solid (85%). R_f_ = 0.44 (7:2 CHCl_3_–MeOH); [α]_D_ = −4 (c 0.20, DMSO). ^1^H-NMR (400 MHz, DMSO-*d*_6_ + 1–2 drops of D_2_O) δ (ppm): 8.99 (1H, s, Tria-H-5), 8.51 (1H, d, *J* = 8.6 Hz, Qu-H-4), 8.25 (1H, d, *J* = 8.6 Hz, Qu-H-3), 8.06–8.01 (2H, m, Qu-H-5, Qu-H-8), 7.82 (1H, pt, *J* = 7.8, 7.4 Hz, Qu-H-6 or Qu-H-7), 7.63 (1H, pt, *J* = 7.6, 7.4 Hz, Qu-H-6 or Qu-H-7), 5.68 (1H, d, *J* = 9.2 Hz, H-1′), 3.90 (1H, pt, *J* = 9.2, 9.1 Hz, H-2′), 3.77–3.72 (1H, m, H-6′a), 3.56–3.49 (2H, m, H-5′, H-6′b), 3.46 (1H, pt, *J* = 9.1, 9.0 Hz, H-3′), 3.34 (1H, pt, *J* = 9.2, 9.0 Hz, H-4′); ^13^C-NMR (90 MHz, DMSO-*d*_6_) δ (ppm): 150.1, 147.5, 147.3 (Tria-C-4, Qu-C-2, Qu-C-8a), 137.3 (Qu-C-4), 130.1 (Qu-C-6 or Qu-C-7), 128.6, 128.1 (Qu-C-5, Qu-C-8), 127.3 (Qu-C-4a), 126.5 (Qu-C-6 or Qu-C-7) 123.3 (Tria-C-5), 118.3 (Qu-C-3), 87.8 (C-1′), 80.0 (C-5′), 76.8 (C-3′), 72.2 (C-2′), 69.5 (C-4′), 60.8 (C-6′). ESI-HRMS positive mode (*m*/*z*): calculated for C_17_H_19_N_4_O_5_^+^ [M + H]^+^ 359.1350; C_17_H_18_N_4_NaO_5_^+^ [M + Na]^+^ 381.1169; C_34_H_36_N_8_NaO_10_^+^ [2M + Na]^+^ 739.2447. Found: [M + H]^+^ 359.1349; [M + Na]^+^ 381.1168; [2M + Na]^+^ 739.2448.

#### 5.1.12. 2-(2′,3′,4′,6′-Tetra-*O*-benzoyl-β-d-glucopyranosyl)-5-(pyridin-2-yl)-1,3,4-oxadiazole (**L-4**)

Prepared from tetrazole [58,59] **2** (5.00 g, 7.71 mmol) and 2-picolinic acid (1.90 g, 14.43 mmol) according to general procedure II. Reaction time: 5 h. Purification by column chromatography (1:1 EtOAc–hexane) and crystallization from EtOH gave 1.96 g white solid (35%). R_f_ = 0.28 (1:1 EtOAc–hexane). [α]_D_ = −92 (c 0.20, CHCl_3_). ^1^H-NMR (400 MHz, CDCl_3_) δ (ppm): 8.81 (1H, d, *J* = 4.8 Hz, Py-H-6), 8.21 (1H, d, *J* = 7.8 Hz, Py-H-3), 8.03–7.81, 7.56–7.27 (22H, m, Ar, Py-H-4, Py-H-5), 6.10 (1H, pt, *J* = 9.4, 9.4 Hz, H-3′), 6.05 (1H, pt, *J* = 9.4, 9.4 Hz, H-2′), 5.86 (1H, pt, *J* = 9.4, 9.4 Hz, H-4′), 5.28 (1H, d, *J* = 9.4 Hz, H-1′), 4.67 (1H, dd, *J* = 12.4, 2.3 Hz, H-6′a), 4.54 (1H, dd, *J* = 12.4, 5.5 Hz, H-6′b), 4.37 (1H, ddd, *J* = 9.4, 5.5, 2.3 Hz, H-5′); ^13^C-NMR (90 MHz, CDCl_3_) δ (ppm): 166.2, 165.8, 165.2, 165.1, 164.9, 162.2 (4 × C=O, OD-C-2, OD-C-5), 150.5 (Py-C-6), 143.2 (Py-C-2), 137.3 (Py-C-4), 133.7, 133.6, 133.5, 133.2, 130.0–128.4 (Ar), 126.2 (Py-C-5), 123.5 (Py-C-3), 77.3 (C-5′), 73.8 (C-3′), 72.1 (C-1′), 70.6 (C-2′), 69.2 (C-4′), 63.3 (C-6′). ESI-HRMS positive mode (*m*/*z*): calculated for C_41_H_31_N_3_NaO_10_^+^ [M + Na]^+^ 748.1902. Found: 748.1907.

#### 5.1.13. 2-(2′,3′,4′,6′-Tetra-*O*-acetyl-β-d-glucopyranosyl)-5-(pyridin-2-yl)-1,3,4-oxadiazole (**L-5**)

To a solution of the 2-(β-d-glucopyranosyl)-5-(pyridin-2-yl)-1,3,4-oxadiazole (**L-10**, 20 mg, 0.065 mmol) in dry pyridine (0.5 mL), acetic anhydride (0.06 mL, 0.635 mmol) was added, and the mixture was stirred at 60 °C. After 1 h, the TLC (1:1 EtOAc–hexane) showed total consumption of **L-10**. The solvent was removed under reduced pressure, and the residue was purified by column chromatography (1:1 EtOAc–hexane). White amorphous solid, yield: 28 mg (90%). R_f_ = 0.21 (1:1 EtOAc–hexane); [α]_D_ = −61 (c 0.19, CHCl_3_). ^1^H-NMR (400 MHz, CDCl_3_) δ (ppm): 8.82 (1H, d, *J* = 4.4 Hz, Py-H-6), 8.26 (1H, d, *J* = 7.9 Hz, Py-H-3), 7.91 (1H, dt, *J* = 7.9, 1.1 Hz, Py-H-4), 7.49 (1H, m, Py-H-5), 5.56 (1H, pt, *J* = 9.8, 9.7 Hz, H-2′), 5.40 (1H, pt, *J* = 9.4, 9.3 Hz, H-3′), 5.24 (1H, pt, *J* = 9.8, 9.7 Hz, H-4′), 4.92 (1H, d, *J* = 10.1 Hz, H-1′), 4.30 (1H, dd, *J* = 12.6, 5.1 Hz, H-6′a), 4.18 (1H, dd, *J* = 12.6, 2.2 Hz, H-6′b), 3.91 (1H, ddd, *J* = 9.7, 5.1, 2.2 Hz, H-5′), 2.09, 2.07, 2.04, 1.94 (4 × 3H, 4 s, 4 × CH_3_); ^13^C-NMR (100 MHz, CDCl_3_) δ (ppm): 170.7, 170.3, 169.4, 169.3 (4 × C=O), 165.1, 162.1 (OD-C-2, OD-C-5), 150.6 (Py-C-6), 143.2 (Py-C-2), 137.4 (Py-C-4), 126.3 (Py-C-5), 123.6 (Py-C-3), 76.9 (C-5′), 73.5 (C-3′), 71.6 (C-1′), 69.7 (C-2′), 68.0 (C-4′), 62.0 (C-6′), 20.8, 20.7 (2), 20.5 (4 × CH_3_). ESI-HRMS positive mode (*m*/*z*): calculated for C_21_H_23_N_3_NaO_10_^+^ [M + Na]^+^ 500.1276. Found: 500.1275.

#### 5.1.14. 2-(2′,3′,4′-Tri-*O*-benzoyl-β-d-xylopyranosyl)-5-(pyridin-2-yl)-1,3,4-oxadiazole (**L-6**)

Prepared from tetrazole [60] **3** (3.00 g, 5.83 mmol) and 2-picolinic acid (1.42 g, 11.53 mmol) according to general procedure II. Reaction time: 5 h. Purification by column chromatography (2:1 EtOAc–hexane) and crystallization from EtOH yielded 2.00 g white solid (58%). R_f_ = 0.35 (4:1 EtOAc–hexane); [α]_D_ = −123 (c 0.20, CHCl_3_). ^1^H-NMR (360 MHz, CDCl_3_) δ (ppm): 8.80 (1H, d, *J* = 4.8 Hz, Py-H-6), 8.21 (1H, d, 7.9 Hz, Py-H-3), 7.99–7.83, 7.57–7.30 (17H, m, Ar, Py-H-4, Py-H-5), 6.05 (1H, pt, *J* = 9.2, 9.2 Hz, H-3′), 5.96 (1H, pt, *J* = 9.2, 9.2 Hz, H-2′), 5.57 (1H, ddd, *J* = 10.1, 9.2, 5.3 Hz, H-4′), 5.15 (1H, d, *J* = 9.2 Hz, H-1′), 4.62 (1H, dd, *J* = 11.4, 5.3 Hz, H-5′eq), 3.80 (1H, pt, *J* = 11.4, 10.1 Hz, H-5′ax); ^13^C-NMR (90 MHz, CDCl_3_) δ (ppm): 165.8, 165.6, 165.1 (2), 162.5 (3 × C=O, OD-C-2, OD-C-5), 150.6 (Py-C-6), 143.3 (Py-C-2), 137.3 (Py-C-4), 133.7, 133.6, 133.5, 130.0–128.5 (Ar), 126.2 (Py-C-5), 123.5 (Py-C-3), 72.9 (C-3′), 72.5 (C-1′), 70.4 (C-2′), 69.6 (C-4′), 67.5 (C-5′). ESI-HRMS positive mode (*m*/*z*): calculated for C_33_H_25_N_3_NaO_8_^+^ [M + Na]^+^ 614.1534. Found: 614.1535.

#### 5.1.15. 2-(2′,3′,4′,6′-Tetra-*O*-acetyl-β-d-galactopyranosyl)-5-(pyridin-2-yl)-1,3,4-oxadiazole (**L-7**)

Prepared from tetrazole [61] **4** (0.50 g, 1.25 mmol) and 2-picolinic acid (0.31 g, 2.50 mmol) according to general procedure II. Reaction time: 2 h. Purification by column chromatography (1:1 EtOAc–hexane) and crystallization from EtOH yielded 0.30 g white solid (50%). R_f_ = 0.17 (1:1 EtOAc–hexane); [α]_D_ = −41 (c 0.21, CHCl_3_). ^1^H-NMR (360 MHz, CDCl_3_) δ (ppm): 8.82 (1H, ddd, *J* = 4.8, 1.8, 0.9 Hz, Py-H-6), 8.27 (1H, dd, *J* = 7.9, 0.9 Hz, Py-H-3), 7.91 (1H, dt, *J* = 7.9, 1.8 Hz, Py-H-4), 7.50 (1H, ddd, *J* = 7.7, 4.8, 1.1 Hz, Py-H-5), 5.67 (1H, pt, *J* = 10.1, 10.0 Hz, H-2′), 5.56 (1H, d, *J* = 3.4 Hz, H-4′), 5.24 (1H, dd, *J* = 10.1, 3.4 Hz, H-3′), 4.89 (1H, d, *J* = 10.0 Hz, H-1′), 4.23–4.11 (3H, m, H-5′, H-6′a, H-6′b), 2.23, 2.06, 2.02, 1.96 (4 × 3H, 4 s, 4 × CH_3_); ^13^C-NMR (90 MHz, CDCl_3_) δ (ppm): 170.4, 170.3, 170.0, 169.4, 165.0, 162.3 (4 × C=O, OD-C-2, OD-C-5), 150.5 (Py-C-6), 143.2 (Py-C-2), 137.3 (Py-C-4), 126.2 (Py-C-5), 123.6 (Py-C-3), 75.6 (C-5′), 72.2 (C-1′), 71.4 (C-3′), 67.3 (C-4′), 66.9 (C-2′), 61.6 (C-6′), 20.8, 20.7, 20.6, 20.5 (4 × CH_3_). ESI-HRMS positive mode (*m*/*z*): calculated for C_21_H_24_N_3_O_10_^+^ [M + H]^+^ 478.1456; C_21_H_23_N_3_NaO_10_^+^ [M + Na]^+^ 500.1276; C_42_H_46_N_6_NaO_20_^+^ [2M + Na]^+^ 977.2659. Found: [M + H]^+^ 478.1454; [M + Na]^+^ 500.1274; [2M + Na]^+^ 977.2653.

#### 5.1.16. 2-(2′,3′,4′,6′-Tetra-*O*-benzoyl-β-d-galactopyranosyl)-5-(pyridin-2-yl)-1,3,4-oxadiazole (**L-8**)

The 2-(β-d-galactopyranosyl)-5-(pyridin-2-yl)-1,3,4-oxadiazole (**L-10**, 20.0 mg, 0.065 mmol) was suspended in dry pyridine (0.5 mL), and benzoyl chloride (36 µL, 0.310 mmol) was added. The reaction mixture was stirred at 60 °C until the TLC (3:2 EtOAc–hexane) showed complete disappearance of the starting material (1 h). The solvent was removed under diminished pressure. The residue was dissolved in CH_2_Cl_2_ (20 mL) and extracted with sat. aq. NaHCO_3_ (10 mL) and then with water (10 mL). The organic phase was dried over MgSO_4_, filtered, and evaporated. Purification by column chromatography (3:2 EtOAc–hexane) yielded 38 mg of white amorphous solid (81%). R_f_ = 0.27 (3:2 EtOAc–hexane); [α]_D_ = +9 (c 0.20, CHCl_3_). ^1^H-NMR (400 MHz, CDCl_3_) δ (ppm): 8.81 (1H, d, *J* = 4.8 Hz, Py-H-6), 8.21 (1H, d, *J* = 7.9 Hz, Py-H-3), 8.17–7.23 (22H, m, Ar, Py-H-4, Py-H-5), 6.32 (1H, pt, *J* = 10.1, 10.0 Hz, H-2′), 6.16 (1H, d, *J* = 3.3 Hz, H-4′), 5.84 (1H, dd, *J* = 10.1, 3.3 Hz, H-3′), 5.30 (1H, d, *J* = 10.0 Hz, H-1′), 4.68 (1H, dd, *J* = 11.1, 6.4 Hz, H-6′a), 4.58 (1H, pt, *J* = 6.4, 5.9 Hz, H-5′), 4.49 (1H, dd, *J* = 11.1, 5.9 Hz, H-6′b); ^13^C-NMR (100 MHz, CDCl_3_) δ (ppm): 166.2, 165.7, 165.6, 165.1, 165.0, 162.4 (4 × C=O, OD-C-2, OD-C-5), 150.5 (Py-C-6), 143.3 (Py-C-2), 137.3 (Py-C-4), 133.8, 133.6, 133.5, 133.4, 130.2–130.0, 129.9–128.5 (Ar), 126.2 (Py-C-5), 123.6 (Py-C-3), 76.1 (C-5′), 72.5, 72.3 (C-1′, C-3′), 68.4 (C-4′), 67.9 (C-2′), 62.3 (C-6′). ESI-HRMS positive mode (*m*/*z*): calculated for C_41_H_31_N_3_NaO_10_^+^ [M + Na]^+^ 748.1902. Found: 748.1901.

#### 5.1.17. 2-(l-Arabino-1′,2′,3′,4′-tetraacetoxybutyl)-5-(pyridin-2-yl)-1,3,4-oxadiazole (**L-9**)

Prepared from tetrazole [33] **5** (3.50 g, 9.77 mmol) and 2-picolinic acid (2.41 g, 19.58 mmol) according to general procedure II. Reaction time: 5 h. Purification by column chromatography (1:1 → 2:1 → 3:1 EtOAc–hexane) yielded 0.57 g of white amorphous solid (13%). R_f_ = 0.27 (4:1 EtOAc–hexane); [α]_D_ = +6 (c 0.20, CHCl_3_). ^1^H-NMR (400 MHz, CDCl_3_) δ (ppm): 8.79 (1H, d, *J* = 4.4 Hz, Py-H-6), 8.26 (1H, d, *J* = 7.9 Hz, Py-H-3), 7.90 (1H, dt, *J* = 7.8, 1.4 Hz, Py-H-4), 7.49 (1H, m, Py-H-5), 6.35 (1H, d, *J* = 2.2 Hz, H-1′), 5.69 (1H, dd, *J* = 9.4, 2.2 Hz, H-2′), 5.36 (1H, ddd, *J* = 9.4, 3.9, 2.3 Hz, H-3′), 4.35 (1H, dd, *J* = 12.6, 2.3 Hz, H-4′a), 4.23 (1H, dd, *J* = 12.6, 3.9 Hz, H-4′b), 2.22, 2.11, 2.10, 2.06 (4 × 3H, 4 s, CH_3_); ^13^C-NMR (100 MHz, CDCl_3_) δ (ppm): 170.6, 169.7, 169.6 (2) (4 × C=O), 164.5, 162.6 (OD-C-2, OD-C-5), 150.4 (Py-C-6), 143.1 (Py-C-2), 137.4 (Py-C-4), 126.3 (Py-C-5), 123.5 (Py-C-3), 68.7 (C-2′), 67.8 (C-3′), 64.4 (C-1′), 61.5 (C-4′), 20.8, 20.7, 20.5 (2) (4 × CH_3_). ESI-HRMS positive mode (*m*/*z*): calculated for C_19_H_22_N_3_O_9_^+^ [M + H]^+^ 436.1351; C_19_H_21_N_3_NaO_9_^+^ [M + Na]^+^ 458.1170. Found: [M + H]^+^ 436.1349; [M + Na]^+^ 458.1170.

#### 5.1.18. 2-(β-d-Glucopyranosyl)-5-(pyridin-2-yl)-1,3,4-oxadiazole (**L-10**)

Prepared from compound **L-4** (0.60 g, 0.83 mmol) according to general procedure III. Purified by column chromatography (9:1 CHCl_3_–MeOH) to give 0.22 g white amorphous solid (87%). R_f_ = 0.29 (8:2 CHCl_3_–MeOH); [α]_D_ = +17 (c 0.20, MeOH). ^1^H-NMR (360 MHz, CD_3_OD) δ (ppm): 8.75 (1H, ddd, *J* = 4.9, 1.7, 0.9 Hz, Py-H-6), 8.25 (1H, dd, *J* = 7.9, 1.1 Hz, Py-H-3), 8.07 (1H, dt, *J* = 7.9, 1.7 Hz, Py-H-4), 7.64 (1H, ddd, *J* = 7.9, 4.9, 1.1 Hz, Py-H-5), 4.68 (1H, d, *J* = 9.9 Hz, H-1′), 3.90 (1H, dd, *J* = 12.2, 1.9 Hz, H-6′a), 3.84 (1H, dd, *J* = 9.9, 8.7 Hz, H-2′), 3.70 (1H, *J* = 12.2, 5.4 Hz, H-6′b), 3.54 (1H, pt, *J* = 8.9, 8.6 Hz, H-3′), 3.54–3.50 (1H, m, H-5′), 3.46 (1H, pt, *J* = 9.3, 8.8 Hz, H-4′); ^13^C-NMR (90 MHz, CD_3_OD) δ (ppm): 166.4, 165.7 (OD-C-2, OD-C-5), 151.4 (Py-C-6), 144.0 (Py-C_-_2), 139.3 (Py-C-4), 127.9 (Py-C-5), 124.5 (Py-C-3), 82.9 (C-5′), 79.1 (C-3′), 74.7 (C-1′), 73.4 (C-2′), 71.3 (C-4′), 62.8 (C-6′). ESI-HRMS positive mode (*m*/*z*): calculated for C_13_H_15_N_3_NaO_6_^+^ [M + Na]^+^ 332.0853. Found: 332.0844.

#### 5.1.19. 2-(β-d-Xylopyranosyl)-5-(pyridin-2-yl)-1,3,4-oxadiazole (**L-11**)

Prepared from compound **L-6** (500 mg, 0.85 mmol) according to general procedure III. Purified by column chromatography (9:1 CHCl_3_–MeOH) to give 82 mg white amorphous solid (35%). R_f_ = 0.27 (9:1 CHCl_3_–MeOH); [α]_D_ = −44 (c 0.20, MeOH). ^1^H-NMR (360 MHz, CD_3_OD) δ (ppm): 8.74 (1H, ddd, *J* = 4.9, 1.6, 0.9 Hz, Py-H-6), 8.24 (1H, ddd, *J* = 7.9, 1.1, 0.9 Hz, Py-H-3), 8.06 (1H, dt, *J* = 7.9, 1.6 Hz, Py-H-4), 7.63 (1H, ddd, *J* = 7.7, 4.9, 1.1 Hz, Py-H-5), 4.59 (1H, d, *J* = 9.8 Hz, H-1′), 4.03 (1H, dd, *J* = 11.1, 5.4 Hz, H-5′eq), 3.84 (1H, pt, *J* = 9.8, 9.1 Hz, H-2′), 3.66 (1H, td, *J* = 10.1, 9.1, 5.4 Hz, H-4′), 3.47 (1H, pt, *J* = 9.1, 9.1 Hz, H-3′), 3.41 (1H, pt, *J* = 11.1, 10.1 Hz, H-5′ax); ^13^C-NMR (90 MHz, CD_3_OD) δ (ppm): 166.4, 165.7 (OD-C-2, OD-C-5), 151.4 (Py-C-6), 144.0 (Py-C-2), 139.3 (Py-C-4), 127.9 (Py-C-5), 124.5 (Py-C-3), 79.2 (C-3′), 75.4 (C-1′), 73.4 (C-2′), 71.7 (C-5′), 71.0 (C-4′). ESI-HRMS positive mode (*m*/*z*): calculated for C_12_H_13_N_3_NaO_5_^+^ [M + Na]^+^ 302.0747; C_24_H_26_N_6_NaO_10_^+^ [2M + Na]^+^ 581.1603. Found: [M + Na]^+^ 302.0747; [2M + Na]^+^ 581.1604.

#### 5.1.20. 2-(β-d-Galactopyranosyl)-5-(pyridin-2-yl)-1,3,4-oxadiazole (**L-12**)

Prepared from compound **L-7** (350 mg, 0.73 mmol) according to general procedure III. Purified by column chromatography (7:2 CHCl_3_–MeOH) to give 177 mg white amorphous solid (78%). R_f_ = 0.33 (7:2 CHCl_3_–MeOH); [α]_D_ = +25 (c 0.21, MeOH). ^1^H-NMR (400 MHz, CD_3_OD) δ (ppm): 8.77 (1H, d, *J* = 4.8 Hz, Py-H-6), 8.27 (1H, d, *J* = 7.9 Hz, Py-H-3), 8.08 (1H, dt, *J* = 7.9, 1.5 Hz, Py-H-4), 7.65 (1H, m, Py-H-5), 4.63 (1H, d, *J* = 9.8 Hz, H-1′), 4.22 (1H, pt, *J* = 9.8, 9.6 Hz, H-2′), 4.01 (1H, d, *J* = 3.2 Hz, H-4′), 3.84–3.73 (3H, m, H-5′, H-6′a, H-6′b), 3.67 (1H, dd, *J* = 9.6, 3.2 Hz, H-3′); ^13^C-NMR (90 MHz, CD_3_OD) δ (ppm): 166.5, 165.7 (OD-C-2, OD-C-5), 151.4 (Py-C-6), 144.1 (Py-C-2), 139.3 (Py-C-4), 127.9 (Py-C-5), 124.5 (Py-C-3), 81.7 (C-5′), 75.8 (C-3′), 75.1 (C-1′), 70.7 (C-4′), 70.2 (C-2′), 62.8 (C-6′). ESI-HRMS positive mode (*m*/*z*): calculated for C_13_H_16_N_3_O_6_^+^ [M + H]^+^ 310.1034; C_13_H_15_N_3_NaO_6_^+^ [M + Na]^+^ 332.0853; C_26_H_30_N_6_NaO_12_^+^ [2M + Na]^+^ 641.1814. Found: [M + H]^+^ 310.1035; [M + Na]^+^ 332.0852; [2M + Na]^+^ 641.1815.

#### 5.1.21. Complex **Ru-1a**

Prepared from complex **Ru-dimer** (50 mg, 0.082 mmol), compound **L-1a** (86 mg, 0.181 mmol, 2.2 eq.), and TlPF_6_ (57 mg, 0.163 mmol) according to general procedure IV. Purified by column chromatography (9:1 CHCl_3_–MeOH) to give 143 mg (98%) yellow powder. R_f_: 0.32 (9:1 CHCl_3_–MeOH). Diastereomeric ratio: 1:1. ^1^H-NMR (400 MHz, CDCl_3_) δ (ppm): 9.25, 9.22 (2 × 1H, 2 d, *J* = 5.5 Hz in each, 2 × Py-H-6), 8.92, 8.79 (2 × 1H, 2 s, 2 × Tria-H-5), 7.96 (2H, t, *J* = 7.6 Hz, 2 × Py-H-4), 7.89 (2H, d, *J* = 7.8 Hz, 2 × Py-H-3), 7.57–7.52 (2H, m, 2 × Py-H-5), 6.00, 5.99 (2 × 1H, 2 d, *J* = 9.4 Hz in each, 2 × H-1′), 5.95–5.83, 5.73–5.67 (10H, m, 2 × 4 × *p*-cym-CH_Ar_, 2 × H-2′), 5.47, 5.46 (2 × 1H, 2 pt, *J* = 9.4, 9.2 Hz in each, 2 × H-3′), 5.35, 5.33 (2 × 1H, 2 pt, *J* = 9.7, 9.7 Hz in each, 2 × H-4′), 4.38, 4.32 (2 × 1H, 2 dd, *J* = 12.8, 4.7 Hz in each, 2 × H-6′a), 4.26–4.15 (4H, m, 2 × H-5′, 2 × H-6′b), 2.79, 2.74 (2 × 1H, 2 hept, *J* = 6.9 Hz in each, 2 × *i*-Pr-CH), 2.22, 2.20 (2 × 3H, 2 s, 2 × C_6_H_4_–C*H_3_*), 2.10, 2.09, 2.05, 1.95, 1.93 (24H, singlets, 2 × 4 × COC*H_3_*), 1.17–1.12 (12H, m, 2 × 2 × *i*-Pr-C*H_3_*); ^13^C-NMR (90 MHz, CDCl_3_) δ (ppm): 170.8, 170.7, 170.0, 169.9, 169.6, 169.5, 169.4, 169.2 (2 × 4 × C=O), 155.5, 155.4 (2 × Py-C-6), 147.6, 147.5, 147.1, 146.7 (2 × Tria-C-4, 2 × Py-C-2), 140.3, 140.2 (2 × Py-C-4), 127.1, 126.9 (2 × Py-C-5), 125.4, 125.3 (2 × Tria-C-5), 122.9 (2) (2 × Py-C-3), 106.3, 105.5, 103.1, 101.8 (2 × 2 × *p*-cym-C_qAr_), 86.8, 86.7 (2 × C-1′), 86.4, 85.4, 85.3, 85.0, 84.8, 83.9, 83.9, 83.1 (2 × 4 × *p*-cym-CH_Ar_), 75.5, 75.3 (2 × C-5′), 73.1, 73.0 (2 × C-3′), 70.2, 69.8 (2 × C-2′), 67.6, 67.5 (2 × C-4′), 61.6, 61.5 (2 × C-6′), 31.1, 31.0 (2 × *i*-Pr-*C*H), 22.5, 22.2, 22.1, 21.7 (2 × 2 × *i*-Pr-*C*H_3_), 20.8–20.3 (2 × 4 × CO*C*H_3_), 18.7 (2) (2 × C_6_H_4_–*C*H_3_). ESI-HRMS positive mode (*m*/*z*): calculated for C_31_H_38_ClN_4_O_9_Ru^+^ [M − PF_6_]^+^ 747.1371. Found: 747.1370.

#### 5.1.22. Complex **Ru-1b**

Prepared from complex **Ru-dimer** (50 mg, 0.082 mmol), compound **L-1b** (95 mg, 0.180 mmol, 2.2 eq.), and TlPF_6_ (57 mg, 0.163 mmol) according to general procedure IV. Purified by column chromatography (9:1 CHCl_3_–MeOH) to give 145 mg (96%) yellow powder. R_f_: 0.55 (9:1 CHCl_3_–MeOH). Diastereomeric ratio: 2:1. ^1^H-NMR (400 MHz, CDCl_3_) δ (ppm): 9.24 (s, minor Tria-H-5), 9.03 (s, major Tria-H-5), 8.75 (d, *J* = 8.8 Hz, major Qu-H-8), 8.68 (d, *J* = 8.7 Hz, minor Qu-H-8), 8.39 (d, *J* = 8.6 Hz, major Qu-H-4), 8.37 (d, *J* = 8.9 Hz, minor Qu-H-4), 8.04–7.89, 7.75–7.70 (2 m, minor and major Qu-H-3, Qu-H-5–Qu-H-7), 6.11 (d, *J* = 9.4 Hz, minor H-1′), 6.08 (d, *J* = 9.2 Hz, major H-1′), 6.03 (pt, *J* = 9.2, 9.0 Hz, major H-2′), 5.97 (pt, *J* = 9.4, 9.0 Hz, minor H-2′), 5.96, 5.95 (2 d, *J* = 6.0 Hz in each, major *p*-cym-CH_Ar_) 5.88, 5.84 (2 d, *J* = 6.0 Hz in each, minor *p*-cym-CH_Ar_) 5.83–5.80 (m, minor and major *p*-cym-CH_Ar_), 5.78 (d, *J* = 6.0 Hz, minor *p*-cym-CH_Ar_), 5.67 (d, *J* = 5.9 Hz, major *p*-cym-CH_Ar_), 5.52 (pt, *J* = 9.2, 9.2 Hz, major H-3′), 5.49 (pt, *J* = 9.5, 9.4 Hz, minor H-3′), 5.39 (pt, *J* = 10.2, 9.7 Hz, minor H-4′), 5.34 (pt, *J* = 10.0, 9.7 Hz, major H-4′), 4.44 (dd, *J* = 12.7, 4.7 Hz, minor H-6′a), 4.33 (dd, *J* = 12.7, 5.1 Hz, major H-6′a), 4.32–4.17 (m, minor and major H-5′, H-6′b), 2.57 (hept, *J* = 6.9 Hz, major *i*-Pr-CH), 2.53 (hept, *J* = 6.9 Hz, minor *i*-Pr-CH), 2.15 (s, minor C_6_H_4_–C*H_3_*), 2.13, 2.09 (singlets, COC*H_3_*), 2.07 (s, major C_6_H_4_–C*H_3_*), 2.06, 2.02, 1.96 (singlets, COC*H_3_*), 1.07, 1.04 (2 d, *J* = 6.9 Hz in each, 2 × major *i*-Pr-C*H*_3_), 1.02, 1.00 (2 d, *J* = 6.9 Hz in each, 2 × minor *i*-Pr-C*H*_3_); ^13^C-NMR (90 MHz, CDCl_3_) δ (ppm): 170.9, 170.0, 169.6, 169.4 (minor 4 × C=O), 170.8, 170.1, 169.8, 169.7 (major 4 × C=O), 149.6, 148.4, 147.5 (major Tria-C-4, Qu-C-2, Qu-C-8a), 149.2, 148.4, 148.9 (minor Tria-C-4, Qu-C-2, Qu-C-8a), 141.3 (minor Qu-C-4), 141.0 (major Qu-C-4), 133.1, 129.6, 129.2, 129.1, 129.1 (minor Qu-C-4a, Qu-C-5–Qu-C-8), 132.7, 129.5, 129.1, 129.1, 129.0 (major Qu-C-4a, Qu-C-5–Qu-C-8), 127.6 (major Tria-C-5), 127.5 (minor Tria-C-5), 119.1 (major Qu-C-3), 118.8 (minor Qu-C-3), 106.3, 102.1 (minor *p*-cym-C_qAr_), 105.4, 102.7 (major *p*-cym-C_qAr_), 88.1, 86.7, 86.5, 86.4, 85.7, 85.1, 84.6, 84.5, 84.3, 84.0 (minor and major *p*-cym-CH_Ar_, minor and major C-1′), 75.6 (minor C-5′), 75.2 (major C-5′), 73.2 (minor C-3′), 73.0 (major C-3′), 70.2 (major C-2′), 69.8 (minor C-2′), 67.5 (major C-4′), 67.7 (minor C-4′), 61.6 (major C-6′), 61.5 (minor C-6′), 31.3 (minor *i*-Pr-*C*H), 31.1 (major *i*-Pr-*C*H), 22.7, 21.6 (major *i*-Pr-*C*H_3_), 22.4, 21.8 (minor *i*-Pr-*C*H_3_), 20.8, 20.7, 20.7, 20.6, 20.4 (2 × 4 × CO*C*H_3_), 18.7 (minor C_6_H_4_–*C*H_3_), 18.5 (major C_6_H_4_–*C*H_3_). ESI-HRMS positive mode (*m*/*z*): calculated for C_35_H_40_ClN_4_O_9_Ru^+^ [M − PF_6_]^+^ 797.1528. Found: 797.1531.

#### 5.1.23. Complex **Ru-2a**

Prepared from complex **Ru-dimer** (10.0 mg, 0.0163 mmol), compound **L-2a** (24.9 mg, 0.0344 mmol, 2.1 eq.), and TlPF_6_ (11.4 mg, 0.0326 mmol, 2 eq.) according to general procedure IV. Purified by column chromatography (95:5 CHCl_3_–MeOH) to give 34.6 mg (93%) orange powder. R_f_: 0.46 (95:5 CHCl_3_–MeOH). Diastereomeric ratio: 1:1. ^1^H-NMR (400 MHz, CDCl_3_) δ (ppm): 9.23, 9.20 (2 × 1H, 2 d, *J* = 5.6 Hz in each, 2 × Py-H-6), 9.02, 8.98 (2 × 1H, 2 s, 2 × Tria-H-5), 8.11–7.75, 7.62–7.24 (46H, m, 2 × 20 × Ar, 2 × Py-H-3–Py-H-5), 6.60, 6.10 (2 × 1H, 2 pt, *J* = 9.4, 9.3 Hz in each, 2 × H-2′), 6.50, 6.43 (2 × 1H, 2 d, *J* = 9.3 Hz in each, 2 × H-1′), 6.22, 6.15 (2 × 1H, 2 pt, *J* = 9.6, 9.4 Hz in each, 2 × H-3′), 5.97, 5.94 (2 × 1H, 2 pt, *J* = 9.7, 9.6 Hz in each, 2 × H-4′), 5.81, 5.65 (2), 5.57 (2), 5.53, 5.47, 5.44 (2 × 4H, 2 × 4 d, *J* = 6.1 Hz in each, 2 × 4 × *p*-cym-CH_Ar_), 4.78–4.52 (2 × 3H, m, 2 × H-5′, 2 × H-6′a, 2 × H-6′b), 2.50, 2.36 (2 × 1H, 2 hept, *J* = 6.9 Hz in each, 2 × *i*-Pr-CH), 2.08, 2.00 (2 × 3H, 2 s, 2 × C_6_H_4_–C*H_3_*), 0.94, 0.91, 0.79, 0.74 (2 × 2 × 3H, 2 × 2 d, *J* = 6.9 Hz in each 2 × 2 × *i*-Pr-C*H*_3_); ^13^C-NMR (100 MHz, CDCl_3_) δ (ppm): 166.3, 166.2, 165.6, 165.5, 165.2 (2), 164.9, 164.8 (2 × 4 × C=O), 155.7, 155.5 (2 × Py-C-6), 147.3, 147.2, 147.0, 146.6 (2 × Tria-C-4, 2 × Py-C-2), 140.1, 140.0 (2 × Py-C-4), 134.4, 134.2, 133.9, 133.8, 133.7 (2), 133.6, 133.5, 133.3, 130.3–128.0 (Ar), 127.2, 126.9 (2 × Py-C-5), 126.2, 123.8 (2 × Tria-C-5), 122.7 (2 × Py-C-3), 105.8, 105.3, 103.9, 102.6 (2 × 2 × *p*-cym-C_qAr_), 87.2, 86.7 (2 × C-1′), 86.4, 85.8, 85.2, 85.0, 84.0, 83.9, 83.2, 82.5 (2 × 4 × *p*-cym-CH_Ar_), 76.1, 75.6 (2 × C-5′), 73.6, 72.7 (2 × C-3′), 71.7, 70.4 (2 × C-2′), 68.7, 68.6 (2 × C-4′), 62.8, 62.7 (2 × C-6′), 31.0 (2) (2 × *i*-Pr-*C*H), 22.5, 22.3, 21.7, 21.4 (2 × 2 × *i*-Pr-*C*H_3_), 18.8, 18.6 (2 × C_6_H_4_–*C*H_3_). ESI-HRMS positive mode (*m*/*z*): calculated for C_51_H_46_ClN_4_O_9_Ru^+^ [M − PF_6_]^+^ 995.2002. Found: 995.1994.

#### 5.1.24. Complex **Ru-3a**

Prepared from complex **Ru-dimer** (50.0 mg, 0.082 mmol), compound **L-3a** (50.4 mg, 0.163 mmol, 2 eq.), and TlPF_6_ (57.0 mg, 0.163 mmol) according to general procedure V. Yield: 107 mg (90%). An analytically pure sample was obtained by recrystallization from *i*PrOH to give 20 mg orange powder. Diastereomeric ratio: 1:1. ^1^H-NMR (400 MHz, CD_3_OD) δ (ppm): 9.42 (2H, d, *J* = 5.4 Hz, 2 × Py-H-6), 9.22, 9.21 (2 × 1H, 2 s, 2 × Tria-H-5), 8.19 (2H, pt, *J* = 7.8, 7.7 Hz, 2 × Py-H-4), 8.10 (2H, d, *J* = 7.8 Hz, 2 × Py-H-3), 7.69–7.66 (2H, m, 2 × Py-H-5), 6.12–6.05 (4H, m, 2 × *p*-cym-CH_Ar_), 5.90–5.84 (4H, m, 2 × *p*-cym-CH_Ar_), 5.88 (2H, d, 2 × H-1′), 3.95–3.89 (4H, m, 2 × H-2′, 2 × H-6′a), 3.77 (2H, dd, *J* = 12.1, 5.3 Hz, 2 × H-6′b), 3.69 (2H, ddd, *J* = 9.5, 5.3, 1.9 Hz, 2 × H-5′), 3.64 (2H, pt, *J* = 9.5, 8.9 Hz, 2 × H-3′), 3.56 (2H, pt, *J* = 9.3, 9.1 Hz, 2 × H-4′), 2.79–2.71 (2H, hept, *J* = 6.7 Hz, 2 × *i*-Pr-CH), 2.22 (6H, s, 2 × C_6_H_4_–C*H_3_*), 1.18–1.09 (12H, m, 2 × 2 × *i*-Pr-C*H_3_*); ^13^C-NMR (100 MHz, CD_3_OD) δ (ppm): 156.8 (2) (2 × Py-C-6), 149.6 (2), 148.2, 148.1 (2 × Py-C-2, 2 × Tria-C-4), 141.5 (2) (2 × Py-C-4), 127.6 (2) (2 × Py-C-5), 125.5, 125.2 (2 × Tria-C-5), 123.6 (2) (2 × Py-C-3), 106.8, 106.6, 104.1, 103.8 (2 × 2 × *p*-cym-C_qAr_), 91.3, 91.2 (2 × C-1′), 87.3, 87.2, 86.3, 86.1, 85.6, 85.5, 84.8, 84.6 (2 × 4 × *p*-cym-CH_Ar_), 81.7, 81.6 (2 × C-5′), 78.2, 78.1 (2 × C-3′), 74.5, 74.3 (2 × C-2′), 70.8, 70.7 (2 × C-4′), 62.3, 62.2 (2 × C-6′), 32.3, 32.2 (2 × *i*-Pr-*C*H), 22.6 (2), 22.0, 21.9 (2 × 2 × *i*-Pr-*C*H_3_), 18.8, 18.7 (2 × C_6_H_4_–*C*H_3_). ESI-HRMS positive mode (*m*/*z*): calculated for C_23_H_30_ClN_4_O_5_Ru^+^ [M − PF_6_]^+^ 579.0946. Found: 579.0946.

#### 5.1.25. Complex **Ru-3b**

Prepared from complex **Ru-dimer** (50.0 mg, 0.082 mmol), compound **L-3b** (58.8 mg, 0.164 mmol, 2 eq.), and TlPF_6_ (57 mg, 0.163 mmol) according to general procedure V. Yield: 121 mg (96%). An analytically pure sample was obtained by recrystallization from *i*PrOH to give 34 mg orange powder. Diastereomeric ratio: 1:1. ^1^H-NMR (400 MHz, CD_3_OD) δ (ppm): 9.42, 9.41 (2 × 1H, 2 s, 2 × Tria-H-5), 8.80 (2H, d, *J* = 8.8 Hz, 2 × Qu-H-8), 8.70, 8.68 (2 × 1H, 2 d, *J* = 8.5 Hz in each, 2 × Qu-H-4), 8.15–8.09, 7.88–7.84 (2 × 4H, m, Qu-H-3, Qu-H-5–Qu-H-7), 6.13–5.96 (2 × 4H, m, 2 × *p*-cym-CH_Ar_), 5.95, 5.93 (2 × 1H, 2 d, *J* = 9.1 Hz in each, 2 × H-1′), 4.00, 3.99 (2 × 1H, 2 pt, *J* = 9.2, 9.1 Hz in each, 2 × H-2′), 3.97, 3.95 (2 × 1H, 2 dd, *J* = 12.1, 2.1 Hz in each, 2 × H-6′a), 3.80 (2H, dd, *J* = 12.1, 5.3 Hz, 2 × H-6′b), 3.74, 3.71 (2 × 1H, ddd, *J* = 9.3, 5.3, 2.1 Hz in each, 2 × H-5′), 3.68, 3.67 (2 × 1H, 2 pt, *J* = 9.0, 8.9 Hz in each, 2 × H-3′), 3.60, 3.59 (2 × 1H, 2 pt, *J* = 9.3, 9.2 Hz in each, 2 × H-4′), 2.48, 2.46 (2 × 1H, 2 hept, *J* = 6.9 Hz in each, 2 × *i*-Pr-CH), 2.20 (6H, s, 2 × C_6_H_4_–C*H_3_*), 1.00, 0.98, 0.92 (2) (2 × 2 × 3H, 4 d, *J* = 6.9 Hz in each, 2 × 2 × *i*-Pr-C*H_3_*); ^13^C NMR (90 MHz, CD_3_OD) δ (ppm): 151.0 (2), 149.7 (2), 149.1 (2), (2 × Tria-C-4, 2 × Qu-C-2, 2 × Qu-C-8a), 142.6 (2 × Qu-C-4), 134.0, 130.7, 130.6 (3), 130.5, 130.1 (2 × Qu-C-4a, 2 × Qu-C-5–Qu-C-8), 127.1, 126.9 (2 × Tria-C-5), 119.5, 119.4 (2 × Qu-C-3), 106.5, 106.3, 104.9, 104.7 (2 × 2 × *p*-cym-C_qAr_), 91.4, 91.2 (2 × C-1′), 88.6, 88.5, 86.5, 86.3, 86.2, 85.8, 84.6, 84.3 (2 × 4 × *p*-cym-CH_Ar_), 81.8, 81.7 (2 × C-5′), 78.2, 78.1 (2 × C-3′), 74.4, 74.3 (2 × C-2′), 70.7 (2) (2 × C-4′), 62.3, 62.20 (2 × C-6′), 32.3, 32.2 (2 × *i*-Pr-*C*H), 22.7, 22.6, 21.6, 21.5 (2 × 2 × *i*-Pr-*C*H_3_), 18.8, 18.7 (2 × C_6_H_4_–*C*H_3_). ESI-HRMS positive mode (*m*/*z*): calculated for C_27_H_32_ClN_4_O_5_Ru^+^ [M − PF_6_]^+^: 629.1103. Found: 629.1103.

#### 5.1.26. Complex **Ru-4**

Prepared from complex **Ru-dimer** (10.0 mg, 0.016 mmol), compound **L-4** (24.9 mg, 0.034 mmol, 2.1 eq.), and TlPF_6_ (11.4 mg, 0.033 mmol) according to general procedure IV. After filtration and removal of the solvent, the residue was dissolved in CHCl_3_ (3 mL), and Et_2_O (6 mL) was added. The precipitated product was filtered off to give 28.4 mg (76%) yellow powder. The product could also be obtained by column chromatographic purification (9:1 CHCl_3_–MeOH), albeit in lower yield (52%). R_f_: 0.38 (9:1 CHCl_3_–MeOH). Diastereomeric ratio: 2:1. ^1^H-NMR (400 MHz, CDCl_3_) δ (ppm): 9.50 (s, minor Py-H-6), 9.26 (s, major Py-H-6), 8.18–7.70, 7.60–7.28 (m, minor and major Ar, Py-H-3–Py-H-5), 6.19 (pt, *J* = 9.8, 9.7 Hz, minor H-3′), 6.16 (pt, *J* = 9.8, 9.5 Hz, major H-2′), 6.08 (pt, *J* = 9.5, 9.5 Hz, major H-3′), 5.88 (pt, *J* = 9.7, 9.5 Hz, major H-4′), 5.86 (pt, *J* = 9.7, 9.6 Hz, minor H-4′), 5.90–5.84 (m, 2 × minor *p*-cym-CH_Ar_), 5.76–5.72 (m, major *p*-cym-CH_Ar_, minor H-2′), 5.68–5.65 (m, 2 × minor *p*-cym-CH_Ar_), 5.53–5.51 (m, major *p*-cym-CH_Ar_), 5.46 (d, *J* = 9.8 Hz, major H-1′), 5.35 (d, *J* = 9.8 Hz, minor H-1′), 5.31–5.29 (m, 2 × major *p*-cym-CH_Ar_), 4.74–4.68 (m, minor and major H-6′a), 4.57–4.52 (m, minor and major H-6′b), 4.50–4.42 (m, minor and major H-5′), 2.78 (hept, *J* = 6.8 Hz, major *i*-Pr-CH), 2.72 (hept, *J* = 6.8 Hz, minor *i*-Pr-CH), 2.02 (s, minor C_6_H_4_–C*H_3_*), 1.95 (s, major C_6_H_4_–C*H_3_*), 1.16, 1.15, 1.12, 1.08 (4 d, *J* = 6.8 Hz in each, minor and major 2 × *i*-Pr-C*H*_3_); ^13^C-NMR (90 MHz, CDCl_3_) δ (ppm): 166.2, 165.8, 165.7, 165.7, 165.3, 165.2, 165.2, 164.9, 164.8, 164.7 (minor and major C=O, OD-C-2, OD-C-5), 158.3 (minor Py-C-6), 156.6 (major Py-C-6), 140.3 (minor Py-C-4), 140.2 (major Py-C-2), 140.1 (major Py-C-4), 139.1 (minor Py-C-2), 134.6, 134.0, 133.9, 133.8, 133.7, 133.5, 133.4, 131.1, 130.6, 130.2–128.5, 127.8 (minor and major Ar, Py-C-5), 125.3 (minor Py-C-3), 125.2 (major Py-C-3), 107.0, 102.3 (minor *p*-cym-C_qAr_), 104.8, 102.1 (major *p*-cym-C_qAr_), 88.7, 84.6, 83.2 (2) (major *p*-cym-CH_Ar_), 85.5, 85.2, 83.8, 83.6 (minor *p*-cym-CH_Ar_), 77.8 (minor C-5′), 77.4 (major C-5′), 73.8, 71.5, 69.8 (major C-1′–C-3′), 72.8, 71.9, 71.4 (minor C-1′–C-3′), 68.8 (major C-4′), 68.7 (minor C-4′), 63.0 (minor C-6′), 62.6 (major C-6′), 31.3 (minor *i*-Pr-*C*H), 31.1 (major *i*-Pr-*C*H), 23.1, 21.3 (major *i*-Pr-*C*H_3_), 22.2, 22.1 (minor *i*-Pr-*C*H_3_), 18.8 (minor C_6_H_4_–*C*H_3_), 18.1 (major C_6_H_4_–*C*H_3_). ESI-HRMS positive mode (*m*/*z*): calculated for C_51_H_45_ClN_3_O_10_Ru^+^ [M − PF_6_]^+^ 996.1843; C_53_H_52_N_3_O_12_Ru^+^ [M − PF_6_ − Cl + OMe + MeOH]^+^ 1024.2604. Found: [M − PF_6_]^+^ 996.1861; [M − PF_6_ − Cl + OMe + MeOH]^+^ 1024.2598.

#### 5.1.27. Complex **Ru-5**

Prepared from complex **Ru-dimer** (10.0 mg, 0.0163 mmol), compound **L-5** (17.9 mg, 0.0375 mmol, 2.3 eq.), and TlPF_6_ (11.4 mg, 0.0326 mmol) according to general procedure IV. Purified by column chromatography (9:1 CHCl_3_–MeOH) to give 21.7 mg (74%) yellow powder. R_f_: 0.54 (9:1 CHCl_3_–MeOH). Diastereomeric ratio: 4:3. ^1^H-NMR (400 MHz, CDCl_3_) δ (ppm): 9.45 (d, *J* = 3.9 Hz, minor Py-H-6), 9.30 (d, *J* = 4.4 Hz, major Py-H-6), 8.23–8.08 (m, minor and major Py-H-3, Py-H-4), 7.88 (m, minor Py-H-5), 7.80 (m, major Py-H-5), 6.01–5.96, 5.82–5.69 (m, minor and major *p*-cym-CH_Ar_), 5.75 (pt, *J* = 9.7, 9.6 Hz, major H-2′), 5.44, (pt, *J* = 9.3, 9.2 Hz, minor H-2′), 5.40 (pt, *J* = 9.4, 9.0 Hz, minor and major H-3′), 5.24 (pt, *J* = 9.8, 9.2 Hz, minor H-4′), 5.23 (pt, *J* = 9.8, 9.8 Hz, major H-4′), 5.03 (d, *J* = 10.0 Hz, major H-1′), 5.00 (d, *J* = 8.7 Hz, minor H-1′), 4.30 (dd, *J* = 12.8, 4.8 Hz, minor and major H-6′a), 4.17 (dd, *J* = 12.8, 2.4 Hz, minor and major H-6′b), 3.99 (ddd, *J* = 10.2, 4.8, 2.4 Hz, minor and major H-5′), 3.03 (hept, *J* = 6.8 Hz, major *i*-Pr-CH), 2.91 (hept, *J* = 6.8 Hz, minor *i*-Pr-CH), 2.23, 2.17, 2.10–2.02 (singlets, minor and major C_6_H_4_–C*H_3_*, COC*H_3_*), 1.36 (d, *J* = 6.8 Hz, major 2 × *i*-Pr-C*H*_3_), 1.30, 1.25 (2 d, *J* = 6.8 Hz in each, minor 2 × *i*-Pr-C*H*_3_); ^13^C-NMR (90 MHz, CDCl_3_) δ (ppm): 170.7, 170.6, 170.1, 170.0, 169.8, 169.6, 169.4 (2 × 4 × C=O), 164.9, 164.6 (major OD-C-2, OD-C-5), 164.9, 164.7 (minor OD-C-2, OD-C-5), 157.6 (minor Py-C-6), 156.4 (major Py-C-6), 140.5 (minor Py-C-4), 140.3 (major Py-C-4), 140.3, 139.5 (minor and major Py-C-2), 130.8 (minor Py-C-5), 130.0 (major Py-C-5), 125.5 (minor Py-C-3), 125.4 (major Py-C-3), 106.4 (minor *p*-cym-C_qAr_), 105.0 (major *p*-cym-C_qAr_), 102.7 (minor *p*-cym-C_qAr_), 102.4 (major *p*-cym-C_qAr_), 88.2, 86.0, 85.6, 83.5, 83.2, 83.1 (minor and major *p*-cym-CH_Ar_), 77.2 (minor C-5′), 77.0 (major C-5′), 73.7, 70.9 (major C-1′ and C-3′), 73.0, 71.2, 70.0 (minor C-1′–C-3′), 68.8 (major C-2′), 67.7 (minor C-4′), 67.6 (major C-4′), 61.9 (minor C-6′), 61.7 (major C-6′), 31.3 (minor *i*-Pr-*C*H), 31.2 (major *i*-Pr-*C*H), 22.9, 21.7 (major *i*-Pr-*C*H_3_), 22.4, 22.0 (minor *i*-Pr-*C*H_3_), 20.8, 20.7, 20.6, 20.6, 20.5 (2 × 4 × CO*C*H_3_), 18.8 (minor C_6_H_4_–*C*H_3_), 18.3 (major C_6_H_4_–*C*H_3_). ESI-HRMS positive mode (*m*/*z*): calculated for C_31_H_37_ClN_3_O_10_Ru^+^ [M − PF_6_]^+^ 748.1211. Found: 748.1212.

#### 5.1.28. Complex **Ru-6**

Prepared from complex **Ru-dimer** (50 mg, 0.082 mmol), compound **L-6** (97 mg, 0.164 mmol, 2 eq.), and TlPF_6_ (57 mg, 0.163 mmol) according to general procedure IV. Purified by column chromatography (9:1 CHCl_3_–MeOH) to give 140 mg (85%) yellow powder. R_f_: 0.67 (9:1 CHCl_3_–MeOH). Diastereomeric ratio: 3:2. ^1^H-NMR (400 MHz, CDCl_3_) δ (ppm): 9.46 (d, *J* = 4.1 Hz, minor Py-H-6), 9.26 (d, *J* = 4.2 Hz, major Py-H-6), 8.18–7.73, 7.60–7.32 (m, minor and major Ar, Py-H-3–Py-H-5), 6.11 (pt, *J* = 9.1, 9.1, major H-2′), 6.06–6.02 (m, major and minor H-3′), 5.86 (d, *J* = 5.4 Hz, minor *p*-cym-CH_Ar_), 5.80–5.76 (m, minor and major *p*-cym-CH_Ar_), 5.74 (pt, *J* = 9.3, 9.1 Hz, minor H-2′), 5.65, 5.63 (2 d, *J* = 6.1 Hz in each, minor *p*-cym-CH_Ar_), 5.61–5.53 (m, major and minor H-4′), 5.53 (d, *J* = 5.4 Hz, major *p*-cym-CH_Ar_), 5.37–5.35 (m, major *p*-cym-CH_Ar_), 5.28 (d, *J* = 9.1 Hz, major H-1′), 5.24 (d, *J* = 9.1 Hz, minor H-1′), 4.64 (dd, *J* = 11.8, 5.1 Hz, minor H-5′eq), 4.60 (dd, *J* = 11.8, 5.4 Hz, major H-5′eq), 3.87 (pt, *J* = 10.9, 10.1, minor and major H-5′ax), 2.80 (hept, *J* = 6.9 Hz, major *i*-Pr-CH), 2.75 (hept, *J* = 6.9 Hz, minor *i*-Pr-CH), 2.04 (s, minor C_6_H_4_–C*H_3_*), 1.99 (s, major C_6_H_4_–C*H_3_*), 1.19–1.11 (m, minor and major 2 × *i*-Pr-C*H*_3_); ^13^C-NMR (90 MHz, CDCl_3_) δ (ppm): 165.7, 165.6 (2), 165.5, 165.4, 165.3, 165.2, 164.9, 164.7, 164.6 (minor and major C=O, OD-C-2, OD-C-5), 157.9 (minor Py-C-6), 157.0 (major Py-C-6), 140.4 (minor Py-C-4), 140.3 (major Py-C-4), 140.0 (major Py-C-2), 139.3 (minor Py-C-2), 134.4, 133.9, 133.8 (2), 133.7, 133.5, 130.8–128.0 (minor and major Ar, Py-C-5), 125.2 (minor Py-C-3), 125.1 (major Py-C-3), 106.3, 104.9, 102.4, 102.1 (minor and major *p*-cym-C_qAr_), 88.4, 86.0, 85.1, 84.3, 83.5, 83.4, 83.3, 83.1 (minor and major *p*-cym-CH_Ar_), 73.1, 72.1, 71.9, 71.8, 70.5, 69.7, 69.5, 69.0 (minor and major C-1′–C-4′), 67.8, 67.4 (minor and major C-5′), 31.2, 31.0 (minor and major *i*-Pr-*C*H), 23.1, 22.4, 21.8, 21.1 (minor and major *i*-Pr-*C*H_3_), 18.6, 18.1 (minor and major C_6_H_4_–*C*H_3_). ESI-HRMS positive mode (*m*/*z*): calculated for C_43_H_39_ClN_3_O_8_Ru^+^ [M − PF_6_]^+^ 862.1473; C_45_H_46_N_3_O_10_Ru^+^ [M − PF_6_ − Cl + OMe + MeOH]^+^ 890.2234. Found: [M − PF_6_]^+^ 862.1470; [M − PF_6_ − Cl + OMe + MeOH]^+^ 890.2231.

#### 5.1.29. Complex **Ru-7**

Prepared from complex **Ru-dimer** (50 mg, 0.082 mmol), compound **L-7** (78 mg, 0.163 mmol, 2 eq.), and TlPF_6_ (57 mg, 0.163 mmol) according to general procedure IV. Purified by column chromatography (9:1 CHCl_3_–MeOH) to give 108 mg (74%) yellow powder. R_f_: 0.54 (9:1 CHCl_3_–MeOH). Diastereomeric ratio: 2:1. ^1^H-NMR (400 MHz, CDCl_3_) δ (ppm): 9.46 (d, *J* = 4.4 Hz, minor Py-H-6), 9.34 (d, *J* = 4.4 Hz, major Py-H-6), 8.24–8.10 (m, minor and major Py-H-4, Py-H-3), 7.87 (m, minor Py-H-5), 7.80 (m, major Py-H-5), 6.01–5.95 (m, minor and major *p*-cym-CH_Ar_), 5.85 (pt, *J* = 10.2, 10.0 Hz major H-2′), 5.82–5.71 (m, minor and major *p*-cym-CH_Ar_), 5.57–5.55 (m, minor and major H-4′), 5.47 (pt, *J* = 10.0, 10.0 Hz minor H-2′), 5.29 (dd, *J* = 10.0, 3.2 Hz, minor H-3′), 5.25 (dd, *J* = 10.0, 3.2 Hz, major H-3′), 5.01 (d, *J* = 10.2 Hz, major H-1′), 4.98 (d, *J* = 10.0 Hz, minor H-1′), 4.23–4.10 (m, minor and major H-5′, H-6′a, H-6′b), 3.01 (hept, *J* = 6.8 Hz, major *i*-Pr-CH), 2.89 (hept, *J* = 6.8 Hz, minor *i*-Pr-CH), 2.24–2.23, 2.17, 2.07–2.04 (singlets, minor and major C_6_H_4_–C*H_3_*, COC*H_3_*), 1.34 (d, *J* = 6.8 Hz, 2 × major *i*-Pr-C*H*_3_), 1.28, 1.24 (2 d, *J* = 6.8 Hz in each, 2 × minor *i*-Pr-C*H*_3_); ^13^C-NMR (90 MHz, CDCl_3_) δ (ppm): 170.3, 170.2, 170.1, 170.0, 169.8, 169.7 (2 × 4 × C=O), 164.8, 164.7, 164.5 (2) (minor and major OD-C-2, OD-C-5), 157.4 (minor Py-C-6), 156.5 (major Py-C-6), 140.3 (minor Py-C-4), 140.1 (major Py-C-4), 139.9 (major Py-C-2), 139.2 (minor Py-C-2), 130.5 (minor Py-C-5), 129.9 (major Py-C-5), 125.3 (minor Py-C-3), 125.2 (major Py-C-3), 106.2, 102.6 (minor *p*-cym-C_qAr_), 105.1, 102.2 (major *p*-cym-C_qAr_), 87.6, 85.0, 83.1, 82.9 (major *p*-cym-CH_Ar_), 85.7, 85.2, 83.3, 83.0 (minor *p*-cym-CH_Ar_), 75.6 (minor C-5′), 75.4 (major C-5′), 71.4, 71.0, 67.0 (major C-1′, C-3′, C-4′), 71.3, 70.7, 67.1, 66.9 (minor C-1′–C-4′), 65.8 (major C-2′), 61.4 (minor C-6′), 61.3 (major C-6′), 31.1 (minor *i*-Pr-*C*H), 31.0 (major *i*-Pr-*C*H), 22.6, 21.4 (major *i*-Pr-*C*H_3_), 22.2, 21.8 (minor *i*-Pr-*C*H_3_), 20.6, 20.6, 20.5, 20.5, 20.4, 20.4 (2 × 4 × CO*C*H_3_), 18.5 (minor C_6_H_4_–*C*H_3_), 18.1 (major C_6_H_4_–*C*H_3_). ESI-HRMS positive mode (*m*/*z*): calculated for C_33_H_44_N_3_O_12_Ru^+^ [M − PF_6_ − Cl + OMe + MeOH]^+^ 776.1973. Found: 776.1973.

#### 5.1.30. Complex **Ru-8**

Prepared from complex **Ru-dimer** (10.0 mg, 0.0163 mmol), compound **L-8** (24.9 mg, 0.0343 mmol, 2.1 eq.), and TlPF_6_ (11.4 mg, 0.0326 mmol) according to general procedure IV. Purified by column chromatography (95:5 CHCl_3_–MeOH) to give 30.3 mg (81%) yellow powder. R_f_: 0.49 (95:5 CHCl_3_–MeOH). Diastereomeric ratio: 5:4. ^1^H-NMR (400 MHz, CDCl_3_) δ (ppm): 9.49 (d, *J* = 4.7 Hz, minor Py-H-6), 9.26 (d, *J* = 5.1 Hz, major Py-H-6), 8.21–7.25 (m, minor and major Ar, Py-H-3–Py-H-5), 6.43 (pt, *J* = 10.1, 10.0 Hz, major H-2′), 6.20 (d, *J* = 3.3 Hz, major H-4′), 6.16 (d, *J* = 2.6 Hz, minor H-4′), 6.02–5.94 (m, minor H-2′ and H-3′), 5.84–5.75 (m, minor and major *p*-cym-CH_Ar_, major H-3′), 5.67, 5.64 (2 d, *J* = 6.1 Hz in each, minor *p*-cym-CH_Ar_) 5.52 (d, *J* = 6.1 Hz, major *p*-cym-CH_Ar_), 5.47 (d, *J* = 10.1 Hz, major H-1′), 5.37 (d, *J* = 8.7 Hz, minor H-1′), 5.32, 5.27 (2 d, *J* = 6.1 Hz in each, major *p*-cym-CH_Ar_), 4.72–4.62 (m, minor and major H-5′, H-6′a), 4.53 (dd, *J* = 10.3, 4.1 Hz, minor H-6′b), 4.44 (dd, *J* = 10.3, 4.0 Hz, major H-6′b), 2.80 (hept, *J* = 6.9 Hz, major *i*-Pr-CH), 2.73 (hept, *J* = 6.9 Hz, minor *i*-Pr-CH), 2.03 (s, minor C_6_H_4_–C*H_3_*), 1.94 (s, major C_6_H_4_–C*H_3_*), 1.17, 1.15 (2 d, *J* = 6.9 Hz in each, 2 × major *i*-Pr-C*H*_3_), 1.14, 1.09 (2 d, *J* = 6.9 Hz in each, 2 × minor *i*-Pr-C*H*_3_); ^13^C-NMR (100 MHz, CDCl_3_) δ (ppm): 166.2, 166.1, 165.9, 165.7, 165.5 (2), 165.4, 165.3, 164.9, 164.8 (2), 164.7 (2 × 4 × C=O, 2 × OD-C-2, 2 × OD-C-5), 157.9 (minor Py-C-6), 156.3 (major Py-C-6), 140.4 (major Py-C-2), 140.3 (minor Py-C-4), 140.2 (major Py-C-4), 139.3 (minor Py-C-2), 134.5, 134.2, 133.9, 133.8 (2), 133.6, 133.5 (2), 130.9–128.1 (minor and major Ar, Py-C-5), 125.3 (minor Py-C-3), 125.2 (major Py-C-3), 106.8, 104.7, 102.3, 102.0 (minor and major *p*-cym-C_qAr_), 88.8, 85.4 (2), 84.5, 83.6, 83.5, 83.0 (2) (minor and major *p*-cym-CH_Ar_), 76.7 (minor C-5′), 76.3 (major C-5′), 72.5 (major C-3′), 72.2 (minor C-1′), 71.7 (major C-1′), 71.3, 68.7 (minor C-2′, C-3′), 68.3 (major C-4′), 68.2 (minor C-4′), 66.8 (major C-2′), 62.4 (minor C-6′), 62.1 (major C-6′), 31.3 (minor *i*-Pr-*C*H), 31.1 (major *i*-Pr-*C*H), 23.2, 21.3 (major *i*-Pr-*C*H_3_), 22.2, 22.0 (minor *i*-Pr-*C*H_3_), 18.7 (minor C_6_H_4_–*C*H_3_), 18.0 (major C_6_H_4_–*C*H_3_). ESI-HRMS positive mode (*m*/*z*): calculated for C_51_H_45_ClN_3_O_10_Ru^+^ [M − PF_6_]^+^ 996.1843. Found: 996.1842.

#### 5.1.31. Complex **Ru-9**

Prepared from complex **Ru-dimer** (50 mg, 0.082 mmol), compound **L-9** (78 mg, 0.179 mmol, 2.2 eq.), and TlPF_6_ (57 mg, 0.163 mmol) according to general procedure IV. Purified by column chromatography (9:1 CHCl_3_–MeOH) to give 130 mg (94%) yellow powder. R_f_: 0.54 (9:1 CHCl_3_–MeOH). Diastereomeric ratio: 1:1. ^1^H-NMR (400 MHz, CDCl_3_) δ (ppm): 9.33 (2H, d, *J* = 4.2 Hz, 2 × Py-H-6), 8.15 (2H, t, *J* = 7.4 Hz, 2 × Py-H-4), 8.07 (2H, d, *J* = 7.4 Hz, 2 × Py-H-3), 7.79 (2H, m, 2 × Py-H-5), 6.52, 6.46 (2 × 1H, 2 d, *J* = 2.3 Hz in each, 2 × H-1′), 6.01–5.95 (4H, m, 2 × *p*-cym-CH_Ar_), 5.84–5.71 (6H, m, 2 × *p*-cym-CH_Ar_, 2 × H-2′), 5.34, 5.30 (2 × 1H, 2 ddd, *J* = 8.9, 4.1, 2.9 Hz in each, 2 × H-3′), 4.36, 4.35 (2 × 1H, 2 dd, *J* = 12.5, 2.9 Hz in each, 2 × H-4′a), 4.21, 4.20 (2 × 1H, 2 dd, *J* = 12.5, 4.1 in each, 2 × H-4′b), 2.98, 2.97 (2 × 1H, 2 hept, *J* = 6.9 Hz in each, 2 × *i*-Pr-CH), 2.29, 2.24, 2.21, 2.18, 2.13, 2.12, 2.11, 2.10, 2.08, 2.08 (30H, singlets, 2 × *i*-Pr-C*H_3_*, 2 × 4 × COC*H_3_*), 1.33–1.29 (12H, m, 2 × 2 × *i*-Pr-C*H_3_*); ^13^C-NMR (90 MHz, CDCl_3_) δ (ppm): 170.7, 170.6, 169.9, 169.8, 169.6, 169.5, 169.4, 169.1 (2 × 4 × C=O), 165.2, 165.1, 164.5 (2) (2 × OD-C-2, 2 × OD-C-5), 156.9, 156.8 (2 × Py-C-6), 140.4 (2) (2 × Py-C-4), 139.8, 139.7 (2 × Py-C-2), 130.3 (2) (2 × Py-C-5), 125.4, 125.3 (2 × Py-C-3), 105.9, 105.7, 102.4, 102.3 (2 × 2 × *p*-cym-C_qAr_), 87.2, 86.6, 85.4, 85.1, 83.5 (2), 83.4, 83.3 (2 × 4 × *p*-cym-CH_Ar_), 68.9, 68.7 (2 × C-2′), 68.0, 67.8 (2 × C-3′), 64.3, 63.7 (2 × C-1′), 61.5 (2) (2 × C-4′), 31.3 (2) (2 × *i*-Pr-*C*H), 22.8, 22.6, 21.9, 21.8 (2 × 2 × *i*-Pr-*C*H_3_), 21.0, 20.9 (2), 20.8, 20.7, 20.6, 20.5 (2) (2 × 4 × CO*C*H_3_), 18.5, 18.4 (2 × C_6_H_4_–*C*H_3_). ESI-HRMS positive mode (*m*/*z*): calculated for C_29_H_35_ClN_3_O_9_Ru^+^ [M − PF_6_]^+^ 706.1105; C_31_H_42_N_3_O_11_Ru^+^ [M − PF_6_ − Cl + OMe + MeOH]^+^ 734.1866. Found: [M − PF_6_]^+^ 706.1106; [M − PF_6_ − Cl + OMe + MeOH]^+^ 734.1868.

#### 5.1.32. Complex **Ru-10**

Prepared from complex **Ru-dimer** (39 mg, 0.064 mmol), compound **L-10** (39 mg, 0.126 mmol, 2 eq.), and TlPF_6_ (44 mg, 0.126 mmol) according to general procedure V. The crude product was triturated with Et_2_O to give 80 mg (87%) orange powder. Diastereomeric ratio: 1:1. ^1^H-NMR (400 MHz, CD_3_OD) δ (ppm): 9.54 (2H, d, *J* = 5.5 Hz, 2 × Py-H-6), 8.40–8.36 (4H, m, 2 × Py-H-3, 2 × Py-H-4), 7.98–7.93 (2H, m, 2 × Py-H-5), 6.19–6.13 (2 × 2H, m, 2 × *p*-cym-CH_Ar_), 5.94–5.92 (2 × 2H, m, 2 × *p*-cym-CH_Ar_), 4.85, 4.83 (2 × 1H, 2 d, *J* = 9.8 Hz in each, 2 × H-1′), 3.92, 3.91 (2 × 1H, 2 dd, *J* = 12.1, 6.1 Hz in each, 2 × H-6′a), 3.83, 3.81 (2 × 1H, 2 pt, *J* = 9.5, 9.3 Hz in each, 2 × H-2′), 3.71, 3.70 (2 × 1H, 2 dd, *J* = 12.1, <1 Hz in each, 2 × H-6′b), 3.59–3.54 (4H, m, 2 × H-3′, 2 × H-5′), 3.45, 3.45 (2 × 1H, 2 pt, *J* = 9.8, 9.5 Hz in each, 2 × H-4′), 2.91, 2.90 (2 × 1H, 2 hept, *J* = 6.9 Hz in each, 2 × *i*-Pr-CH), 2.23 (6H, s, 2 × C_6_H_4_–C*H_3_*), 1.28–1.23 (12H, m, 2 × 2 × *i*-Pr-C*H_3_*); ^13^C-NMR (90 MHz, CD_3_OD) δ (ppm): 168.5 (2), 166.0 (2) (2 × OD-C-2, 2 × OD-C-5), 158.3, 158.3 (2 × Py-C-6), 142.0 (2) (2 × Py-C-4), 141.6, 141.5 (2 × Py-C-2), 131.1 (2) (2 × Py-C-5), 126.6, 126.5 (2 × Py-C-3), 107.3, 107.2, 103.5, 103.4 (2 × 2 × *p*-cym-C_qAr_), 87.4, 87.2, 85.9, 85.8, 85.1, 85.0, 84.9, 84.8 (2 × 4 × *p*-cym-CH_Ar_), 83.2, 83.1 (2 × C-5′), 78.8, 78.7 (2 × C-3′), 74.6 (2) (2 × C-1′), 73.3, 73.2 (2 × C-2′), 71.1 (2) (2 × C-4′), 62.6 (2) (2 × C-6′), 32.4 (2) (2 × *i*-Pr-*C*H), 22.8, 22.7, 22.1, 22.0 (2 × 2 × *i*-Pr-*C*H_3_), 18.8 (2) (2 × C_6_H_4_–*C*H_3_). ESI-HRMS positive mode (*m*/*z*): calculated for C_23_H_29_ClN_3_O_6_Ru^+^ [M − PF_6_]^+^ 580.0786; C_25_H_36_N_3_O_8_Ru^+^ [M − PF_6_ − Cl + OMe + MeOH]^+^ 608.1548. Found: [M − PF_6_]^+^ 580.0787; [M − PF_6_ − Cl + OMe + MeOH]^+^ 608.1545.

#### 5.1.33. Complex **Ru-11**

Prepared from complex **Ru-dimer** (10.0 mg, 0.016 mmol), compound **L-11** (9.1 mg, 0.033 mmol, 2 eq.), and TlPF_6_ (11.4 mg, 0.033 mmol) according to general procedure V. Purified by recrystallization from an *i*PrOH–Et_2_O solvent mixture (2 mL and 5 mL, respectively) to give 9.5 mg (42%) yellow powder. Diastereomeric ratio: 1:1. ^1^H-NMR (400 MHz, CD_3_OD) δ (ppm): 9.53 (2H, d, *J* = 5.4 Hz, 2 × Py-H-6), 8.38–8.34 (4H, m, 2 × Py-H-3, 2 × Py-H-4), 7.95–7.92 (2H, m, 2 × Py-H-5), 6.18–6.12 (2 × 2H, m, 2 × *p*-cym-CH_Ar_), 5.94–5.90 (2 × 2H, m, 2 × *p*-cym-CH_Ar_), 4.77, 4.76 (2 × 1H, 2 d, *J* = 9.6 Hz in each, 2 × H-1′), 4.07, 4.06 (2 × 1H, 2 dd, *J* = 11.1, 5.3 Hz in each, 2 × H-5′eq), 3.81, 3.80 (2 × 1H, 2 pt, *J* = 9.6, 9.0 Hz in each, 2 × H-2′), 3.68, 3.65 (2 × 1H, 2 ddd, *J* = 10.5, 8.9, 5.3 Hz in each, 2 × H-4′), 3.51 (2H, pt, *J* = 9.0, 8.9 Hz, 2 × H-3′), 3.47, 3.46 (2 × 1H, 2 pt, *J* = 11.1, 10.5 Hz in each, 2 × H-5′ax), 2.91 (2H, 2 hept, *J* = 6.8 Hz, 2 × *i*-Pr-CH), 2.22 (6H, s, 2 × C_6_H_4_–C*H_3_*), 1.28–1.23 (12H, m, 2 × 2 × *i*-Pr-C*H_3_*); ^13^C-NMR (100 MHz, CD_3_OD) δ (ppm): 168.8, 168.6, 166.1, 166.0 (2 × OD-C-2, 2 × OD-C-5), 158.2 (2) (2 × Py-C-6), 142.0 (2) (2 × Py-C-4), 141.6, 141.5 (2 × Py-C-2), 131.1 (2) (2 × Py-C-5), 126.6, 126.5 (2 × Py-C-3), 107.4 (2), 103.5, 103.4 (2 × 2 × *p*-cym-C_qAr_), 87.4, 87.3, 86.0, 85.9, 85.0 (2), 84.9, 84.8 (2 × 4 × *p*-cym-CH_Ar_), 79.0, 78.9 (2 × C-3′), 75.4, 75.3 (2 × C-1′), 73.5, 73.3, (2 × C-2′), 71.8, 71.7 (2 × C-5′), 70.8 (2) (2 × C-4′), 32.4 (2) (2 × *i*-Pr-*C*H), 22.7 (2), 22.0 (2) (2 × 2 × *i*-Pr-*C*H_3_), 18.7 (2) (2 × C_6_H_4_–*C*H_3_). ESI-HRMS positive mode (*m*/*z*): calculated for C_22_H_27_ClN_3_O_5_Ru^+^ [M − PF_6_]^+^ 550.0680; C_24_H_34_N_3_O_7_Ru^+^ [M − PF_6_ − Cl + OMe + MeOH]^+^ 578.1442. Found: [M − PF_6_]^+^ 550.0672; [M − PF_6_ − Cl + OMe + MeOH]^+^ 578.1432.

#### 5.1.34. Complex **Ru-12**

Prepared from complex **Ru-dimer** (10.0 mg, 0.016 mmol), compound **L-12** (10.1 mg, 0.033 mmol, 2 eq.), and TlPF_6_ (11.4 mg, 0.033 mmol) according to general procedure V. Purified by recrystallization from an *i*PrOH–Et_2_O solvent mixture (2 mL and 5 mL, respectively) to give 11.9 mg (50%) yellow powder. Diastereomeric ratio: 1:1. ^1^H-NMR (360 MHz, CD_3_OD) δ (ppm): 9.55 (2H, d, *J* = 5.6 Hz, 2 × Py-H-6), 8.40–8.35 (4H, m, 2 × Py-H-3, 2 × Py-H-4), 7.96–7.93 (2H, m, 2 × Py-H-5), 6.19–6.13 (2 × 2H, m, 2 × *p*-cym-CH_Ar_), 5.94–5.92 (2 × 2H, m, 2 × *p*-cym-CH_Ar_), 4.78, 4.76 (2 × 1H, 2 d, *J* = 9.8 Hz in each, 2 × H-1′), 4.18, 4.15 (2 × 1H, 2 pt, *J* = 9.8, 9.4 in each, 2 × H-2′), 4.01 (2H, d, *J* = 3.2 Hz, 2 × H-4′), 3.84–3.73 (6H, m, 2 × H-5′, 2 × H-6′a, 2 × H-6′b), 3.69, 3.68 (2 × 1H, 2 dd, *J* = 9.4, 3.2 in each, 2 × H-3′), 2.91 (2H, 2 hept, *J* = 6.9 Hz, 2 × *i*-Pr-CH), 2.23 (6H, s, 2 × C_6_H_4_–C*H_3_*), 1.27–1.23 (12H, m, 2 × 2 × *i*-Pr-C*H_3_*); ^13^C-NMR (90 MHz, CD_3_OD) δ (ppm): 168.7, 168.6, 166.0, 165.9 (2 × OD-C-2, 2 × OD-C-5), 158.1 (2) (2 × Py-C-6), 141.9 (2) (2 × Py-C-4), 141.6, 141.5 (2 × Py-C-2), 131.1 (2) (2 × Py-C-5), 126.5, 126.4 (2 × Py-C-3), 107.4, 107.3, 103.5, 103.4 (2 × 2 × *p*-cym-C_qAr_), 87.2, 87.1, 86.0, 85.8, 85.0 (2), 84.8, 84.7 (2 × 4 × *p*-cym-CH_Ar_), 82.0, 81.9 (2 × C-5′), 75.6, 75.5, 75.1, 75.0 (2 × C-1′, 2 × C-3′), 70.6, 70.5, 70.2, 70.0 (2 × C-2′, 2 × C-4′), 62.7 (2) (2 × C-6′), 32.4 (2) (2 × *i*-Pr-*C*H), 22.7, 22.6, 22.1, 22.0 (2 × 2 × *i*-Pr-*C*H_3_), 18.7 (2) (2 × C_6_H_4_–*C*H_3_). ESI-HRMS positive mode (*m*/*z*): calculated for C_23_H_29_ClN_3_O_6_Ru^+^ [M − PF_6_]^+^ 580.0786; C_25_H_36_N_3_O_8_Ru^+^ [M − PF_6_ − Cl + OMe + MeOH]^+^ 608.1548. Found: [M − PF_6_]^+^ 580.0792; [M − PF_6_ − Cl + OMe + MeOH]^+^ 608.1553.

#### 5.1.35. Complex **Ru-13**

Prepared from complex **Ru-dimer** (20.0 mg, 0.033 mmol), 1-phenyl-4-(pyridine-2-yl)-1,2,3-triazole [34] (**L-13**, 14.5 mg, 0.065 mmol, 2 eq.), and TlPF_6_ (22.8 mg, 0.065 mmol) according to general procedure V. Purified by recrystallization from a CHCl_3_–Et_2_O solvent mixture (3 mL and 6 mL, respectively) to give 36.8 mg (88%) yellow powder. Racemic mixture. ^1^H-NMR (400 MHz, acetone-*d*_6_) δ (ppm): 9.62 (1H, s, Tria-H-5), 9.57 (1H, ddd, *J* = 5.6, 1.4, 0.9 Hz, Py-H-6), 8.29 (1H, dt, *J* = 7.6, 1.4 Hz, Py-H-4), 8.25 (1H, ddd, *J* = 7.9, 1.9, 0.9 Hz, Py-H-3), 8.12–8.09 (2H, m, Ph), 7.79–7.68 (4H, m, Ph, Py-H-5), 6.27, 6.24, 6.04, 5.99 (4 × 1H, 4 d, *J* = 6.2 Hz in each, 4 × *p*-cym-CH_Ar_), 2.91 (1H, hept, *J* = 6.9 Hz, *i*-Pr-CH), 2.29 (3H, s, C_6_H_4_–C*H_3_*), 1.23, 1.19 (2 × 3H, 2 d, *J* = 6.9 Hz in each, 2 × *i*-Pr-C*H*_3_); ^13^C-NMR (90 MHz, acetone-*d*_6_) δ (ppm): 156.6 (Py-C-6), 149.2, 148.2 (Tria-C-4, Py-C-2), 141.1 (Py-C-4), 131.6, 131.2, 127.3, 124.0, 123.3, 121.9 (Ph, Py-C-3, Py-C-5, Tria-C-5), 106.2, 103.4 (2 × *p*-cym-C_qAr_), 87.4, 85.8, 85.1, 84.5 (4 × *p*-cym-CH_Ar_), 31.9 (*i*-Pr-*C*H), 22.6, 21.9 (2 × *i*-Pr-*C*H_3_), 18.7 (C_6_H_4_–*C*H_3_). ESI-HRMS positive mode (*m*/*z*): calculated for C_23_H_24_ClN_4_Ru^+^ [M − PF_6_]^+^: 493.0730. Found: 493.0709.

#### 5.1.36. Complex **Ru-14**

Prepared from complex **Ru-dimer** (20.0 mg, 0.033 mmol), 2-phenyl-5-(pyridine-2-yl)-1,3,4-oxadiazole [35,36] (**L-14**, 14.6 mg, 0.065 mmol, 2 eq.), and TlPF_6_ (22.8 mg, 0.065 mmol) according to general procedure V. Purified by recrystallization from a CHCl_3_–Et_2_O solvent mixture (3 mL and 6 mL, respectively) to give 34.9 mg (84%) yellow powder. Racemic mixture. ^1^H-NMR (360 MHz, acetone-*d*_6_) δ (ppm): 9.69 (1H, ddd, *J* = 5.6, 1.3, 0.8 Hz, Py-H-6), 8.54 (1H, ddd, *J* = 7.8, 1.6, 0.8 Hz, Py-H-3), 8.48 (1H, dt, *J* = 7.8, 1.3 Hz, Py-H-4), 8.33–8.29 (2H, m, Ph), 8.01 (1H, ddd, *J* = 7.8, 5.6, 1.6 Hz, Py-H-5), 7.85–7.72 (3H, m, Ph), 6.30–6.27, 6.07–6.04 (2 × 2H, 2 m, 4 × *p*-cym-CH_Ar_), 3.03 (1H, hept, *J* = 6.9 Hz, *i*-Pr-CH), 2.31 (3H, s, C_6_H_4_–C*H_3_*), 1.32, 1.30 (2 × 3H, 2 d, *J* = 6.9 Hz in each, 2 × *i*-Pr-C*H*_3_); ^13^C-NMR (90 MHz, acetone-*d*_6_) δ (ppm): 168.2, 164.7 (OD-C-2, OD-C-5), 157.8 (Py-C-6), 141.6 (Py-C-4), 141.5 (Py-C-2), 134.9, 130.7, 130.3, 128.5, 125.9, 122.7 (Ph, Py-C-3, Py-C-5), 106.7, 102.9 (2 × *p*-cym-C_qAr_), 86.9, 85.6, 84.7, 84.5 (4 × *p*-cym-CH_Ar_), 32.0 (*i*-Pr-*C*H), 22.7, 22.0 (2 × *i*-Pr-*C*H_3_), 18.8 (C_6_H_4_–CH_3_). ESI-HRMS positive mode (*m*/*z*): calculated for C_23_H_23_ClN_3_ORu^+^ [M − PF_6_]^+^: 494.0556. Found: 494.0553.

### 5.2. Determination of the Distribution Coefficients (logD)

Prior to the experiments, *n*-octanol was saturated with aqueous PBS solution (pH = 7.40) and vice versa. The corresponding complex (approximately 0.2–0.3 mg) was dissolved in a mixture of 2.50 mL of pre-saturated *n*-octanol and 2.50 mL of pre-saturated PBS buffer, and the mixture was vigorously stirred for 3 days. According to the NMR stability measurements, this time was necessary to reach equilibrium between the various ionic complex species. Due to the lipophilic/hydrophilic character of the complexes, those with benzoyl protection or the non-sugar derivative could mostly be found in the *n*-octanol, while the acetyl and non-protected complexes could be found in the aqueous PBS phase. The appropriate separated solution was centrifuged (ScanSpeed 406G instrument, 4000 RPM for 5 min), and the absorption of the “stock solution” was measured (VWR UV-1600PC Spectrophotometer, 270–420 nm). Then 2.00 mL of stock solution was stirred vigorously with 16.00 mL of pre-saturated, clean *n*-octanol or PBS solution. After 1 day, the phases were separated and centrifuged, and the absorption of the solution was measured again. From the absorption difference of the stock solutions, the distribution coefficient (D) was calculated according to the previously described formulae [66].

### 5.3. Visualization

All structures were drawn using Chemdraw Professional 17.0.

### 5.4. Cell Culture Materials and the Source of the Platinum Compounds

Carboplatin, oxaliplatin, cisplatin, rucaparib, Trolox, and MitoTempo were from Sigma-Aldrich (St. Louis, MO, USA). All other materials for cellular experiments were from Sigma-Aldrich, unless otherwise stated.

### 5.5. Cell Culture

Cells were cultured under standard cell culture conditions, 37 °C, 5% CO_2_, humidified atmosphere. A2780 cells were cultured in RMPI 1640 medium supplemented with 10% fetal calf serum, 2 mM glutamine, and 1% penicillin–streptomycin (Sigma-Aldrich). ID8 cells were cultured in high-glucose DMEM (4.5 g/L glucose) supplemented with 4% fetal calf serum, 2 mM glutamine, 1% penicillin–streptomycin (Sigma-Aldrich), and 1% ITS supplement (Sigma-Aldrich I3146). U251 cells were maintained in MEM (Sigma-Aldrich), 10% fetal bovine serum (Sigma-Aldrich), 1% penicillin/streptomycin (Invitrogene), and 2 mM glutamine. MCF7 cells were maintained in MEM (Sigma-Aldrich), 10% fetal bovine serum (Sigma-Aldrich), 1% penicillin/streptomycin (Invitrogen, Waltham, MA, USA), and 2 mM glutamine. Capan2 cells were maintained in MEM (Sigma-Aldrich), 10% fetal bovine serum (Sigma-Aldrich), 1% penicillin/streptomycin (Invitrogen), and 2 mM glutamine. *Human primary dermal fibroblasts* were cultured in low-glucose DMEM (1 g/L glucose) supplemented with 20% fetal calf serum, 2 mM glutamine, and 1% penicillin–streptomycin (Sigma-Aldrich).

### 5.6. Methylthiazolyldiphenyl-Tetrazolium Bromide (MTT) Reduction Assay

The MTT reduction assay assesses the activity of mitochondrial complex I, and it was used to assess rapid toxicity. The MTT reduction assay was performed similarly to [67]. Cells were seeded in 96-well plates. The next day, cells were treated with the compounds for 4 h in the concentrations indicated in a cell incubator. At the end of treatment, MTT was added in 0.5 mg/mL final concentration, and cells were incubated at 37 °C in a cell incubator. Then, culture media was removed, the reduced MTT dye was resolved in dimethyl sulfoxide (DMSO), and plates were measured in a plate photometer (Thermo Scientific Multiscan GO spectrophotometer, Waltham, MA, USA) at 540 nm. On each plate, wells were designed to contain untreated/vehicle-treated cells. In calculations, the readings for these wells were considered as 1, and all readings were expressed relative to these values.

### 5.7. Sulforhodamine B (SRB) Proliferation Assay

The SRB accumulation assay assesses protein content in a sample, and it was used to assess cell proliferation. The SRB accumulation assay was performed similarly to [68]. Cells were seeded in 96-well plates. The next day, cells were treated with the compounds for 48 h in the concentrations indicated in a cell incubator. At the end of treatment, cells were fixed with 10% trichloroacetic acid (TCA). Fixed cells were stained with SRB (0.4% *m*/*v* dissolved in 1% acetic acid) for 60 min. Fixed cells were washed in 1% acetic acid three times; acetic acid was removed, and cells were left to dry. Protein-bound SRB was released by adding 100 µL of 10 mM Tris base. Plates were measured in a plate photometer (Thermo Scientific Multiscan GO spectrophotometer, Waltham, MA, USA) at 540 nm. On each plate, wells were designed to contain untreated cells. In calculations, the readings for these wells were considered as 1, and all readings were expressed relative to these values.

### 5.8. Annexin V–FITC, PI Double Staining

Annexin V–PI double staining was applied to assess apoptotic and necrotic cell death similar to [37,38,69]. A2780 cells were treated with the indicated compounds at the concentration corresponding to their IC_50_ value for 2, 4, and 48 h. The 4 and 48 h time points correspond to the time points for the MTT and SRB assays, while the 2 h time point reflects the optimum time point for the detection of apoptotic or necrotic cell death [37,38,69]. Quadrants were set on the basis of the FITC and PI values observed for the vehicle-treated cells.

### 5.9. Western Blotting

The preparation of protein extracts, the separation of protein extracts in SDS-polyacrylamide gel electrophoresis, and the subsequent Western blotting were performed as described in [70] using the antibodies in Table 4. Enhanced chemiluminescence was developed using ChemiDoc Imager, (Bio-Rad, Hercules, CA, USA). Densitometry was performed using Image Lab Touch Software, Bio-Rad, Hercules, CA, USA).

### 5.10. Statistical Analysis

Statistical analysis was performed using Graphpad Prism version 8.0.1. Values were tested for normal distribution using the D’Agostino and Pearson normality test. When necessary, values were log-normalized or were normalized using the Box–Cox normalization method [71], as indicated in the figure captions. The level of significance of the subsequent statistical test and post hoc test is indicated in the figure captions. Nonlinear regression was performed using the built-in “[Inhibitor] vs. response—variable slope (four parameters), least square fit” utility of Graphpad that yielded IC_50_ and Hill slope values.

## Figures and Tables

**Table 1:** Synthesis of 1-(β-d-glucopyranosyl)-4-hetaryl-1,2,3-triazoles.

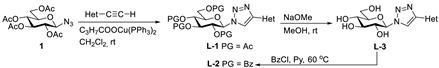				
Het		Yields (%)		
		**L-1**	**L-2**	**L-3**
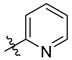	**a**	95	87	76
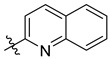	**b**	85	-	85

**Table 2:** Synthesis of [(η^6^-*p*-cym)Ru^II^(N-N)Cl]PF_6_ complexes containing the monosaccharide-based 5-(pyridin-2-yl)-1,3,4-oxadiazoles.

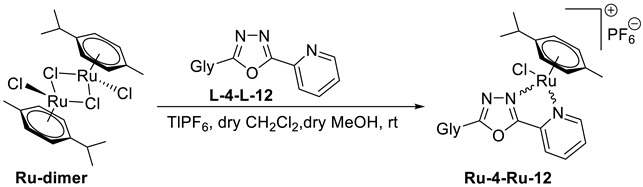								
Yield (%) (Diastereomeric Ratio)								
Gly			Gly			Gly		
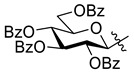	**Ru-4**	76 (2:1)	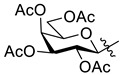	**Ru-7**	74 (2:1)	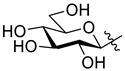	**Ru-10**	87 (1:1)
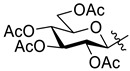	**Ru-5**	74 (4:3)	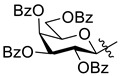	**Ru-8**	81 (5:4)	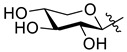	**Ru-11**	42 (1:1)
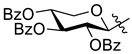	**Ru-6**	85 (3:2)	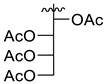	**Ru-9**	94 (1:1)	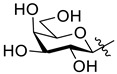	**Ru-12**	50 (1:1)

**Table 3:** The lipophilicity parameters (logD) and kinetic properties of the biologically active ruthenium complexes identified in the study and the reference platinum compounds (IC_50_ (µM)).

Compound	logD	A2780		ID8		Fibroblast		U251		MCF7		Capan2	
		IC_50_	Hill Slope	IC_50_	Hill Slope	IC_50_	Hill Slope	IC_50_	Hill Slope	IC_50_	Hill Slope	IC_50_	Hill Slope
**Ru-2a**	2.85	0.9	1.95	1.53	2.50	N/A	0.78	2.82	1.75	1.29	1.85	2.18	2.43
**Ru-4**	2.41	6.19	2.62	5.66	3.04	N/A	N/A	4.17	2.46	3.74	2.01	3.27	1.81
**Ru-6**	2.04	8.54	3.74	6.76	3.59	24.63	3.52	6.61	2.13	6.69	2.13	4.97	1.66
**Ru-8**	2.44	4.27	3.50	7.94	3.31	N/A	N/A	4.49	2.50	3.75	2.06	3.82	1.66
**Ru-13**	0.44	11.97	1.57										
**Ru-14**	1.63	513.5	0.77										
Cisplatin		1.21	1.20	10.81	0.99	11.90	1.53						
Carboplatin		27.98	1.02	N/A	0.62	N/A	N/A						
Oxaliplatin		0.12	1.27	4.59	1.58	0.83	0.37						

N/A—could not be calculated/had no effect.

**Table 4:** The list of antibodies used in the current study.

Antibody	Company	Catalog Number	Dilution
4-Hydroxynonenal (4HNE)	Abcam	ab46545	1:1000
Anti-mouse IgG, HRP-linked	Sigma-Aldrich	A9044	1:2000
Anti-rabbit IgG, HRP-linked	Cell Signaling Technology	7074	1:2000
Anti-β-actin−Peroxidase	Sigma-Aldrich	A3854	1:20,000

## Data Availability

Primary biological data for this manuscript are available at https://figshare.com/s/83d6f7cf80102ce05c2c (accessed on 16 September 2021). (DOI:10.6084/m9.figshare.13024601).

## Supplementary Materials

The following are available online at https://www.mdpi.com/article/10.3390/ijms221910454/s1, Table S1. Selected ^1^H-NMR data of **Ru-dimer**, the 1-(β-d-glucopyranosyl)-4-hetaryl-1,2,3-triazoles (**L-1–L-3**), and their half-sandwich Ru(II) complexes (**Ru-1–Ru-3**); Table S2. Changes in the chemical shifts of selected ^1^H-NMR resonances as a result of the complex formation for **Ru-1–Ru-3**; Table S3. Selected ^13^C-NMR data of **Ru-dimer**, the 1-(β-d-glucopyranosyl)-4-hetaryl-1,2,3-triazoles (**L-1–L-3**) and their half-sandwich Ru(II) complexes (**Ru-1–Ru-3**); Table S4. Changes in the chemical shifts of selected ^13^C-NMR resonances as a result of the complex formation for **Ru-1–Ru-3**; Table S5. Selected ^1^H-NMR data of **Ru-dimer**, the monosaccharide-based 5-(pyridine-2-yl)-1,3,4-oxadiazoles (**L-4–L-12**), and their half-sandwich Ru(II) complexes (**Ru-4–Ru-12**); Table S6. Changes in the chemical shifts of selected ^1^H-NMR resonances as a result of the complex formation for **Ru-4–Ru-12**; Table S7. Selected ^13^C-NMR data of **Ru-dimer**, the monosaccharide-based 5-(pyridine-2-yl)-1,3,4-oxadiazoles (**L-4–L-12**), and their half-sandwich Ru(II) complexes (**Ru-4–Ru-12**); Table S8. Changes in the chemical shifts of selected ^13^C-NMR resonances as a result of the complex formation for **Ru-4–Ru-12**, along with copies of ^1^H- and ^13^C-NMR spectra; Figure S1. A representative example for the stability of the complexes in aqueous medium; Table S9. Distribution coefficient of the synthesized complexes (logD).
