# Supplementary material for: Ruthenium Half-Sandwich Type Complexes with Bidentate Monosaccharide Ligands Show Antineoplastic Activity in Ovarian Cancer Cell Models through Reactive Oxygen Species Production
Source: Int J Mol Sci. 2021 Sep 28;22(19):10454. doi: 10.3390/ijms221910454 (PMC8508960; doi:10.3390/ijms221910454)
Supplement: Supplementary file 1 [file ijms-22-10454-s001.zip › ijms-1238143-supplementary.pdf]

SUPPORTING INFORMATION

Ruthenium half-sandwich type complexes with bidentate monosaccharide ligands show antineoplastic activity in ovarian cancer cell models through reactive oxygen species production

István Kacsir[#1,2], Adrienn Sipos[#3], Gyula Ujlaki[3], Péter Buglyó[4], László Somsák[1], Péter Bai*[3,5,6], Éva Bokor*[1]

[1]Department of Organic Chemistry, University of Debrecen, PO Box 400, H-4002 Debrecen, Hungary;

[2]Doctoral School of Chemistry, University of Debrecen, PO Box 400, H-4002 Debrecen, Hungary;

[3]Department of Medical Chemistry, Faculty of Medicine, University of Debrecen, H-4032 Debrecen, Hungary;

[4]Department of Inorganic & Analytical Chemistry, Faculty of Sciences and Technology, University of Debrecen, H-4032 Debrecen, Hungary;

[5]NKFIH-DE Lendület Laboratory of Cellular Metabolism, H-4032 Debrecen, Hungary;

[6]Research Center for Molecular Medicine, Faculty of Medicine, University of Debrecen, H-4032 Debrecen, Hungary

[#]equal contribution

*corresponding authors

Correspondence should be sent to:

Éva Bokor, PhD, University of Debrecen, Department of Organic Chemistry, , PO Box 400, H-4002 Debrecen, Hungary, Tel. +36 52 512 900 ext 22474; e-mail: bokor.eva@science.unideb.hu

Péter Bai, PhD, DSc University of Debrecen, Department of Medical Chemistry, H-4032 Debrecen, Egyetem tér 1., Hungary, Tel. +36 52 412 345; Fax. +36 52 412 566, e-mail: baip@med.unideb.hu

Table of contents:

1. **Selected NMR data for the synthesized compounds..2**
2. **Copies of ^{1}H and ^{13}C NMR spectra...7**
3. **A representative example for the stability of the complexes in aqueous medium.....37**
4. **Table S9. Distribution coefficient of the synthesized complexes (logD) 38**

1. Selected NMR data for the synthesized compounds

Table S1. Selected ^{1}H NMR data of **Ru-dimer**, the 1-(β-D-glucopyranosyl)-4-hetaryl-1,2,3-triazoles (**L-1–L-3**) and their half-sandwich Ru(II) complexes (**Ru-1–Ru-3**)									
	Tria-H-5	Py-H-6	Py-H-4 or Qu-H-4	H-1’	H-2’	*p*-cym-CH_{Ar}	*i*-Pr-CH	C_6H_4-CH_3	*i*-Pr-CH_3
**Ru-dimer** ($CDCl_3$)	-	-	-	-	-	5.48; 5.34	2.93	2.16	1.28
**Ru-dimer** (CD_3OD)	-	-	-	-	-	5.88; 5.66	3.31	2.20	1.30
**Ru-dimer** (DMSO-d_6)	-	-	-	-	-	5.81; 5.77	2.82	2.08	1.18
**L-1a** ($CDCl_3$)	8.42	8.61	7.78	5.94	5.51	-	-	-	-
**L-1b** ($CDCl_3$)	8.63	-	8.24	5.99	5.59	-	-	-	-
**L-2a** ($CDCl_3$)	8.61	8.61	7.86-7.76*	6.34	6.07	-	-	-	-
**L-3a** (CD_3OD)	8.63	8.59	7.92	5.70	3.96	-	-	-	-
**L-3b** (DMSO-d_6)	8.99	-	8.51	5.68	3.90	-	-	-	-
**Ru-1a** ($CDCl_3$)	8.92; 8.79	9.25; 9.22	7.96	6.00; 5.99	5.95-5.88*	5.95-5.83, 5.73-5.67*	2.79; 2.74	2.22; 2.20	1.17-1.12*
**Ru-1b** ($CDCl_3$)	9.03; 9.24	-	8.39; 8.37	6.08; 6.11	6.03; 5.97	5.96-5.67*	2.57; 2.53	2.07; 2.15	1.07, 1.04; 1.02, 1.00
**Ru-2a** ($CDCl_3$)	9.02; 8.98	9.23; 9.20	7.82-7.75*	6.50; 6.43	6.60; 6.10	5.81-5.44*	2.50; 2.36	2.08; 2.00	0.94, 0.91; 0.79, 0.74
**Ru-3a** (CD_3OD)	9.22; 9.21	9.42	8.19	5.88; 5.88	3.95-3.89*	6.12-6.05, 5.90-5.84*	2.79-2.71*	2.22	1.18-1.09*
**Ru-3b** (DMSO-d_6)	9.42; 9.41	-	8.70; 8.68	5.95; 5.93	4.00; 3.99	6.13-5.96*	2.48; 2.46	2.20	1.00, 0.98; 0.92, 0.92
*Overlapping signals.									

Table S2. Changes of the chemical shifts of selected ^{1}H NMR resonances as a result of the complex formation for **Ru-1–Ru-3** ($\Delta = \delta_{complex} – \delta_{ligand\ or\ \mathbf{Ru\text{-}dimer}}$).								
**Triazolok**	Δ(Tria-H-5)	Δ(Py-H-6)	Δ(Py-H-4) or Δ(Qu-H-4)	Δ(H-1’)	Δ(H-2’)	*i*-Pr-CH	C_6H_4-CH_3	*i*-Pr-CH_3
**Ru-1a** ($CDCl_3$)	+0.50 +0.37	+0.64 +0.61	+0.18	+0.06 +0.05	*	-0.14 -0.19	+0.06 +0.04	*
**Ru-1b** ($CDCl_3$)	+0.40 +0.61	-	+0.15 +0.13	+0.09 +0.12	+0.44 +0.38	-0.36 -0.40	-0.09 -0.01	-0.21, -0.24 -0.26, -0.28
**Ru-2a** ($CDCl_3$)	+0.41 +0.37	+0.62 +0.59	*	+0.16 +0.09	+0.53 +0.03	-0.43 -0.57	-0.08 -0.16	-0.34, -0.37 -0.49, -0.54
**Ru-3a** (CD_3OD)	+0.59 +0.58	+0.83	+0.27	+0.18 +0.18	*	*	+0.02	*
**Ru-3b** (DMSO-d_6)	+0.43 +0.42	-	+0.19 +0.17	+0.27 +0.25	+0.10 +0.09	-0.34; -0.36	+0.12	-0.18, -0.20 -0.26, -0.26
*Due to signal overlaps these resonances could not be assigned precisely.								

Table S3. Selected ^{13}C NMR data of **Ru-dimer**, the 1-(β-D-glucopyranosyl)-4-hetaryl-1,2,3-triazoles (**L-1–L-3**) and their half-sandwich Ru(II) complexes (**Ru-1–Ru-3**)

	Tria-C-5	Py-C-6	Py-C-5	Py-C-4 or Qu-C-4	Py-C-3 or Qu-C-3	C-1'	C-2'	*p*-cym-C_{qAr}	*p*-cym-CH_{Ar}	*i*-Pr-*C*H	C_6H_4-$\mathit{C}H_3$
**Ru-dimer** ($CDCl_3$)	-	-	-	-	-	-	-	101.2; 96.8	81.3; 80.5	30.6	19.0
**Ru-dimer** (CD_3OD)	-	-	-	-	-	-	-	102.7; 98.5	80.4; 79.3	32.6	18.9
**Ru-dimer** (DMSO-d_6)	-	-	-	-	-	-	-	106.4; 100.1	86.4; 85.5	30.0	17.9
**L-1a** ($CDCl_3$)	120.7	149.7	123.3	137.0	120.5	86.0	70.7	-	-	-	-
**L-1b** ($CDCl_3$)	121.4	-	-	137.0	118.7	86.0	70.7	-	-	-	-
**L-2a** ($CDCl_3$)	121.0	149.6	123.3	137.1	120.6	86.4	71.3	-	-	-	-
**L-3a** (CD_3OD)	123.4	150.5	124.6	138.9	121.7	89.8	74.1	-	-	-	-
**L-3b** (DMSO-d_6)	123.3	-	-	137.3	118.3	87.8	72.2	-	-	-	-
**Ru-1a** ($CDCl_3$)	125.4; 125.3	155.5; 155.4	127.1; 126.9	140.3; 140.2	122.9	86.8; 86.7	70.2; 69.8	106.3, 101.8; 105.5, 103.1	86.4-83.1*	31.1; 31.0	18.7
**Ru-1b** ($CDCl_3$)	127.6; 127.5	-	-	141.0; 141.3	119.1; 118.8	88.1-84.0*	70.2; 69.8	105.4, 102.7; 106.3, 102.1	88.1-84.0*	31.1; 31.3	18.5; 18.7
**Ru-2a** ($CDCl_3$)	126.2; 123.8	155.7; 155.5	127.2; 126.9	140.1; 140.0	122.7	87.2; 86.7	71.7; 70.4	105.8, 102.6; 105.3, 103.9	86.4-82.5*	31.0	18.8; 18.6
**Ru-3a** (CD_3OD)	125.5; 125.2	156.8	127.6	141.5	123.6	91.3; 91.2	74.5; 74.3	106.8, 103.8; 106.6, 104.1	87.3-84.6*	32.3; 32.2	18.8; 18.7
**Ru-3b** (DMSO-d_6)	127.1; 126.9	-	-	142.6	119.5; 119.4	91.4; 91.2	74.4; 74.3	106.5, 104.7; 106.3, 104.9	88.6-84.3*	32.3; 32.2	18.8; 18.7

*Signals were not assigned precisely.

Table S4. Changes of the chemical shifts of selected ^{13}C NMR resonances as a result of the complex formation for **Ru-1–Ru-3** ($\Delta = \delta_{complex} – \delta_{ligand\ or\ \mathbf{Ru\text{-}dimer}}$)

	Δ(Tria-C-5)	Δ(Py-C-6)	Δ(Py-C-5)	Δ(Py-C-4) or Δ(Qu-C-4)	Δ(Py-C-3) or Δ(Qu-C-3)	Δ(C-1')	Δ(C-2')	Δ (*p*-cym-C_{qAr})	Δ(*i*-Pr-CH)	Δ(C_6H_4-CH_3)
**Ru-1a** ($CDCl_3$)	+4.7 +4.6	+5.8 +5.7	+3.8 +3.6	+3.3 +3.2	+2.4	+0.8 +0.7	-0.5 -0.9	+5.1, +5.0 +4.3, +4.6	+0.5 +0.4	-0.3
**Ru-1b** ($CDCl_3$)	+6.2 +6.1	-	-	+4.0 +4.3	+0.4 +0.1	*	-0.5 -0.9	+4.2, +5.9 +5.1, +5.3	+0.5 +0.7	-0.5 -0.3
**Ru-2a** ($CDCl_3$)	+5.2 +2.8	+6.1 +5.9	+3.9 +3.6	+3.0 +2.9	+2.1	+0.8 +0.3	+0.4 +0.1	+4.6, +5.8 +4.1, +7.1	+0.4	-0.2 -0.4
**Ru-3a** (CD_3OD)	+2.1 +1.8	+6.3	+3.0	+2.6	+1.9	+1.5 +1.4	+0.4 +0.2	+4.1, +5.3 +3.9, +5.6	-0.3 -0.4	-0.1 -0.2
**Ru-3b** (DMSO-d_6)	+3.8 +3.6	-	-	+5.3	+1.2 +1.1	+3.6 +3.4	+0.2 +0.1	+0.1; +4.6 -0.1; +4.8	+2.3; +2.2	+0.9 +0.8

*Due to uncertain signal assignment the data could not be calculated precisely.

**Table S5.** Selected ^{1}H NMR data of **Ru-dimer**, the monosaccharide-based 5-(pyridine-2-yl)-1,3,4-oxadiazoles (**L-4–L-12**) and their half-sandwich Ru(II) complexes (**Ru-4–Ru-12**)							
	Py-H-6	Py-H-5	H-1'	H-2'	*p*-cym-CH_{Ar}	*i*-Pr-CH	C_6H_4-CH_3
**Ru-dimer** ($CDCl_3$)	-	-	-	-	5.48; 5.34	2.93	2.16
**Ru-dimer** (CD_3OD)	-	-	-	-	5.88; 5.66	3.31	2.20
**L-4** ($CDCl_3$)	8.81	7.48	5.28	6.05	-	-	-
**L-5** ($CDCl_3$)	8.82	7.49	4.92	5.56	-	-	-
**L-6** ($CDCl_3$)	8.80	7.47	5.15	5.96	-	-	-
**L-7** ($CDCl_3$)	8.82	7.50	4.89	5.67	-	-	-
**L-8** ($CDCl_3$)	8.81	7.47	5.30	6.32	-	-	-
**L-9** ($CDCl_3$)	8.79	7.49	6.35	5.69	-	-	-
**L-10** (CD_3OD)	8.75	7.64	4.68	3.84	-	-	-
**L-11** (CD_3OD)	8.74	7.63	4.59	3.84	-	-	-
**L-12** (CD_3OD)	8.77	7.65	4.63	4.22	-	-	-
**Ru-4** ($CDCl_3$)	9.26; 9.50	7.77; 7.91	5.46; 5.35	6.16; 5.72	5.90-5.51*	2.78; 2.72	1.95; 2.02
**Ru-5** ($CDCl_3$)	9.30; 9.45	7.80; 7.88	5.03; 5.00	5.75; 5.44	6.01-5.96, 5.82-5.69*	3.03; 2.91	2.23-2.02*
**Ru-6** ($CDCl_3$)	9.26; 9.46	7.77; 7.89	5.28; 5.24	6.11; 5.74	5.86-5.35*	2.80; 2.75	1.99; 2.04
**Ru-7** ($CDCl_3$)	9.34; 9.46	7.80; 7.87	5.01; 4.98	5.85; 5.47	6.01-5.95, 5.82-5.71*	3.01; 2.89	2.24-2.04*
**Ru-8** ($CDCl_3$)	9.26; 9.49	7.80; 7.93	5.47; 5.37	6.43; 6.02-5.94*	5.84-5.27*	2.80; 2.73	1.94; 2.03
**Ru-9** ($CDCl_3$)	9.33	7.79	6.52; 6.46	5.84-5.71*	6.01-5.95, 5.84-5.71*	2.98; 2.97	2.29-2.08*
**Ru-10** (CD_3OD)	9.54	7.95; 7.94	4.85; 4.83	3.83; 3.81	6.19-6.13, 5.94-5.92*	2.91; 2.90	2.23
**Ru-11** (CD_3OD)	9.53	7.95-7.92*	4.77; 4.76	3.81; 3.80	6.18-6.12, 5.94-5.90*	2.91	2.22
**Ru-12** (CD_3OD)	9.55	7.95; 7.94	4.78; 4.76	4.18; 4.15	6.19-6.13, 5.94-5.92*	2.91	2.23
*Overlapping signals.							

**Table S6.** Changes of the chemical shifts of selected ^{1}H NMR resonances as a result of the complex formation for **Ru-4–Ru-12** ($\Delta = \delta_{complex}–\delta_{ligand\ or\ \mathbf{Ru\text{-}dimer}}$)						
	Δ(Py-H-6)	Δ(Py-H-5)	Δ(H-1')	Δ(H-2')	Δ(*i*-Pr-CH)	Δ(C_6H_4-CH_3)
**Ru-4** ($CDCl_3$)	+0.45<br>+0.69	+0.29<br>+0.43	+0.18<br>+0.07	+0.11<br>-0.33	-0.15<br>-0.21	-0.21<br>-0.14
**Ru-5** ($CDCl_3$)	+0.48<br>+0.63	+0.31<br>+0.39	+0.11<br>+0.08	+0.19<br>-0.12	+0.10<br>-0.02	*
**Ru-6** ($CDCl_3$)	+0.46<br>+0.66	+0.30<br>+0.42	+0.13<br>+0.09	+0.15<br>-0.22	-0.13<br>-0.18	-0.17<br>-0.12
**Ru-7** ($CDCl_3$)	+0.52<br>+0.64	+0.30<br>+0.37	+0.12<br>+0.09	+0.18<br>-0.20	+0.08<br>-0.04	*
**Ru-8** ($CDCl_3$)	+0.45<br>+0.68	+0.33<br>+0.46	+0.17<br>+0.07	+0.11<br>*	-0.13<br>-0.20	-0.22<br>-0.13
**Ru-9** ($CDCl_3$)	+0.54	+0.30	+0.17<br>+0.11	*	+0.05<br>+0.04	*
**Ru-10** (CD_3OD)	+0.79	+0.31<br>+0.30	+0.17<br>+0.15	-0.01<br>-0.03	-0.40<br>-0.41	+0.03
**Ru-11** (CD_3OD)	+0.79	*	+0.18<br>+0.17	-0.03<br>-0.04	-0.40	+0.02
**Ru-12** (CD_3OD)	+0.78	+0.30<br>+0.29	+0.15<br>+0.13	-0.04<br>-0.07	-0.40	+0.03
*Due to signal overlaps these resonances could not be assigned precisely.						

Table S7. Selected ^{13}C NMR data of **Ru-dimer**, the monosaccharide-based 5-(pyridine-2-yl)-1,3,4-oxadiazoles (**L-4–L-12**) and their half-sandwich Ru(II) complexes (**Ru-4–Ru-12**)

	Py-C-6	Py-C-5	Py-C-4	Py-C-3	Py-C-2	*p*-cym-C_{qAr}	*p*-cym-CH_{Ar}	*i*-Pr-*C*H	C_6H_4-*C*H_3
**Ru-dimer** ($CDCl_3$)	-	-	-	-	-	101.2; 96.8	81.3; 80.5	30.6	19.0
**Ru-dimer** (CD_3OD)	-	-	-	-	-	102.7; 98.5	80.4; 79.3	32.6	18.9
**L-4** ($CDCl_3$)	150.5	126.2	137.3	123.5	143.2	-	-	-	-
**L-5** ($CDCl_3$)	150.6	126.3	137.4	123.6	143.2	-	-	-	-
**L-6** ($CDCl_3$)	150.6	126.2	137.3	123.5	143.3	-	-	-	-
**L-7** ($CDCl_3$)	150.5	126.2	137.3	123.6	143.2	-	-	-	-
**L-8** ($CDCl_3$)	150.5	126.2	137.3	123.6	143.3	-	-	-	-
**L-9** ($CDCl_3$)	150.4	126.3	137.4	123.5	143.1	-	-	-	-
**L-10** (CD_3OD)	151.4	127.9	139.3	124.5	144.0	-	-	-	-
**L-11** (CD_3OD)	151.4	127.9	139.3	124.5	144.0	-	-	-	-
**L-12** (CD_3OD)	151.4	127.9	139.3	124.5	144.1	-	-	-	-
**Ru-4** ($CDCl_3$)	156.6; 158.3	131.2-128.5*	140.1; 140.3	125.2; 125.3	140.2; 139.1	104.8, 102.1; 107.0, 102.3	88.7-83.2; 85.5-83.6*	31.1; 31.3	18.1; 18.8
**Ru-5** ($CDCl_3$)	156.4; 157.6	130.0; 130.8	140.3; 140.5	125.4; 125.5	140.3; 139.5	105.0, 102.4; 106.4, 102.7	88.2-83.1*	31.2; 31.3	18.3; 18.8
**Ru-6** ($CDCl_3$)	157.0; 157.9	130.8-128.5*	140.3; 140.4	125.1, 125.2	140.0; 139.3	104.9, 102.1; 106.3, 102.4	88.4-83.1*	31.0; 31.2	18.1; 18.6
**Ru-7** ($CDCl_3$)	156.5; 157.4	129.9; 130.5	140.1; 140.3	125.2; 125.3	139.9; 139.2	105.1, 102.2; 106.2, 102.6	87.6-82.9; 85.7-83.0*	31.0; 31.1	18.1; 18.5
**Ru-8** ($CDCl_3$)	156.3; 157.9	130.9-128.5*	140.2; 140.3	125.2; 125.3	140.2; 139.3	104.7, 102.0; 106.8, 102.3	88.8-83.0*	31.1; 31.3	18.0; 18.7
**Ru-9** ($CDCl_3$)	156.9; 156.8	130.3	140.4	125.4; 125.3	139.8; 139.7	105.9, 102.4; 105.7, 102.3	87.2-83.3*	31.3	18.5; 18.4
**Ru-10** (CD_3OD)	158.3	131.1	142.0	126.6; 126.5	141.6; 141.5	107.3, 103.5 107.2, 103.4	87.4-84.8*	32.4	18.8
**Ru-11** (CD_3OD)	158.2	131.1	142.0	126.6; 126.5	141.6; 141.5	107.4, 103.5 107.4, 103.4	87.4-84.8*	32.4	18.7
**Ru-12** (CD_3OD)	158.1	131.1	141.9	126.5; 126.4	141.6; 141.5	107.4, 103.5 107.3, 103.4	87.2-84.7*	32.4	18.7

*Signals were not assigned precisely.

Table S8. Changes of the chemical shifts of selected ^{13}C NMR resonances as a result of the complex formation for **Ru-4–Ru-12** ($\Delta = \delta_{complex} - \delta_{ligand\ or\ \mathbf{Ru\text{-}dimer}}$)

	Δ(Py-C-6)	Δ(Py-C-5)	Δ(Py-C-4)	Δ(Py-C-3)	Δ(Py-C-2)	Δ(*p*-cym-C_{qAr})	*i*-Pr-*C*H	C_6H_4-*C*H_3
**Ru-4** ($CDCl_3$)	+6.1<br>+7.8	*	+2.8<br>+3.0	+1.7<br>+1.8	-3.0<br>-4.1	+3.6, +5.3<br>+5.8, +5.5	+0.5<br>+0.7	-0.9<br>-0.2
**Ru-5** ($CDCl_3$)	+5.8<br>+7.0	+3.7<br>+4.5	+2.9<br>+3.1	+1.8<br>+1.9	-2.9<br>-3.7	+3.8, +5.6<br>+5.2, +5.9	+0.6<br>+0.7	-0.7<br>-0.2
**Ru-6** ($CDCl_3$)	+6.4<br>+7.3	*	+3.0<br>+3.1	+1.6<br>+1.7	-3.3<br>-4.0	+3,7, +5.3<br>+5.1, +5.6	+0.4<br>+0.6	-0.9<br>-0.4
**Ru-7** ($CDCl_3$)	+6.0<br>+6.9	+3.7<br>+4.3	+2.8<br>+3.0	+1.6<br>+1.7	-3.3<br>-4.0	+3.9, +5.4<br>+5.0, +5.8	+0.4<br>+0.5	-0.9<br>-0.5
**Ru-8** ($CDCl_3$)	+5.8<br>+7.4	*	+2.9<br>+3.0	+1.6<br>+1.7	-3.1<br>-4.0	+3.5, +5.2<br>+5.6, +5.5	+0.5<br>+0.7	-1.0<br>-0.3
**Ru-9** ($CDCl_3$)	+6.5<br>+6.4	+4.0	+3.0	+1.9<br>+1.8	-3.3<br>-3.4	+4.7, +5.6<br>+4.5, +5.5	+0.7	-0.5<br>-0.6
**Ru-10** (CD_3OD)	+6.9	+3.2	+2.7	+2.1<br>+2.0	-2.4<br>-2.5	+4.6, +5.0<br>+4.5, +4.9	-0.2	-0.1
**Ru-11** (CD_3OD)	+6.8	+3.2	+2.7	+2.1<br>+2.0	-2.4<br>-2.5	+4.7, +5.0<br>+4.7, +4.9	-0.2	-0.2
**Ru-12** (CD_3OD)	+6.7	+3.2	+2.6	+2.0<br>+1.9	-2.5<br>-2.6	+4.7, +5.0<br>+4.6, +4.9	-0.2	-0.2

*Due to uncertain signal assignment the data could not be calculated precisely.

2. Copies of ^{1}H and ^{13}C NMR spectra

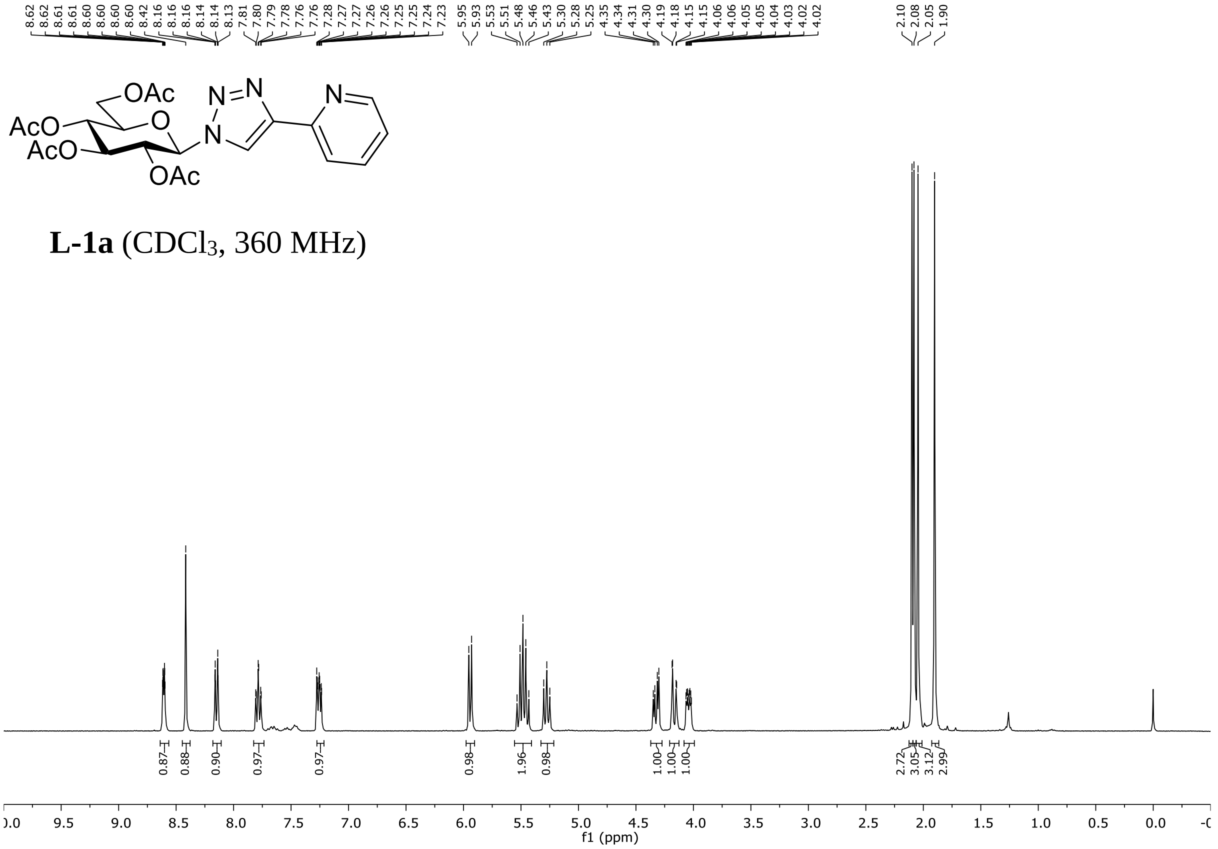

L-1a ($CDCl_3$, 360 MHz)

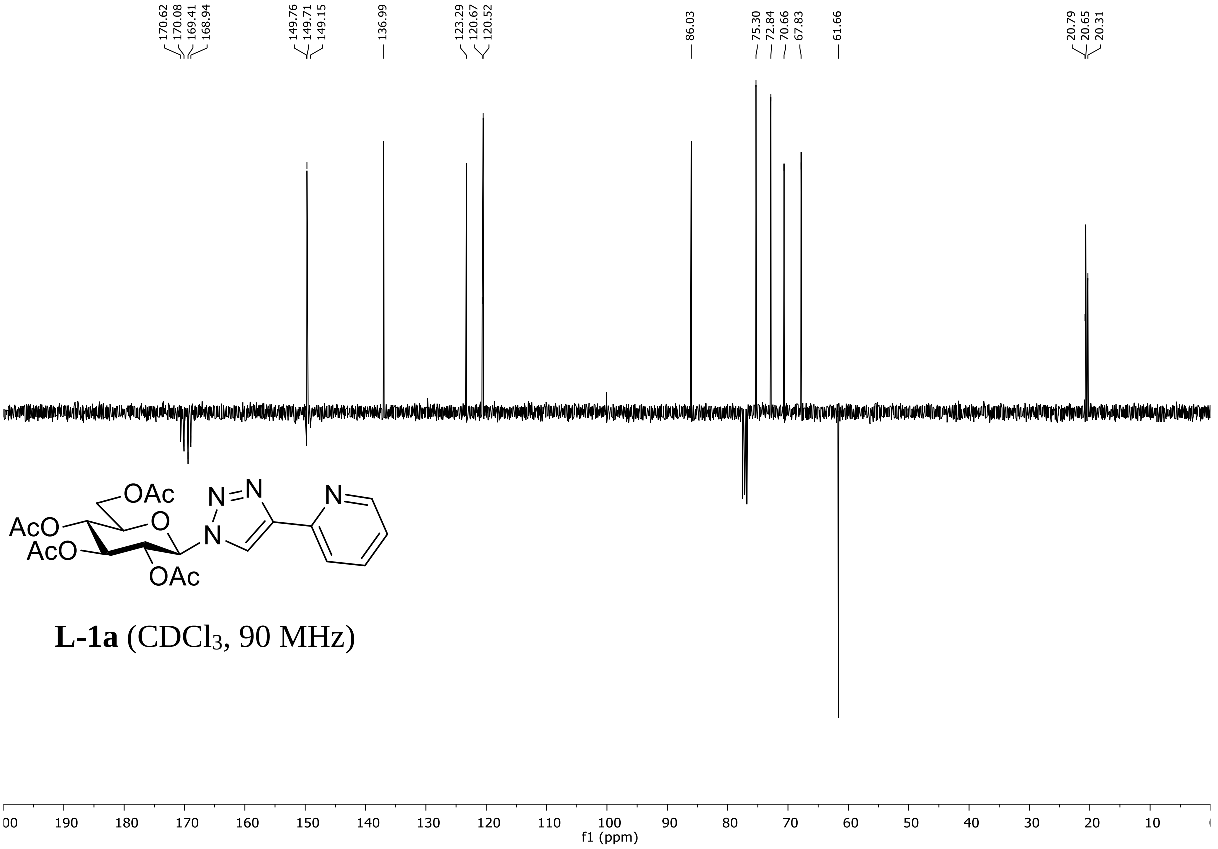

L-1a ($CDCl_3$, 90 MHz)

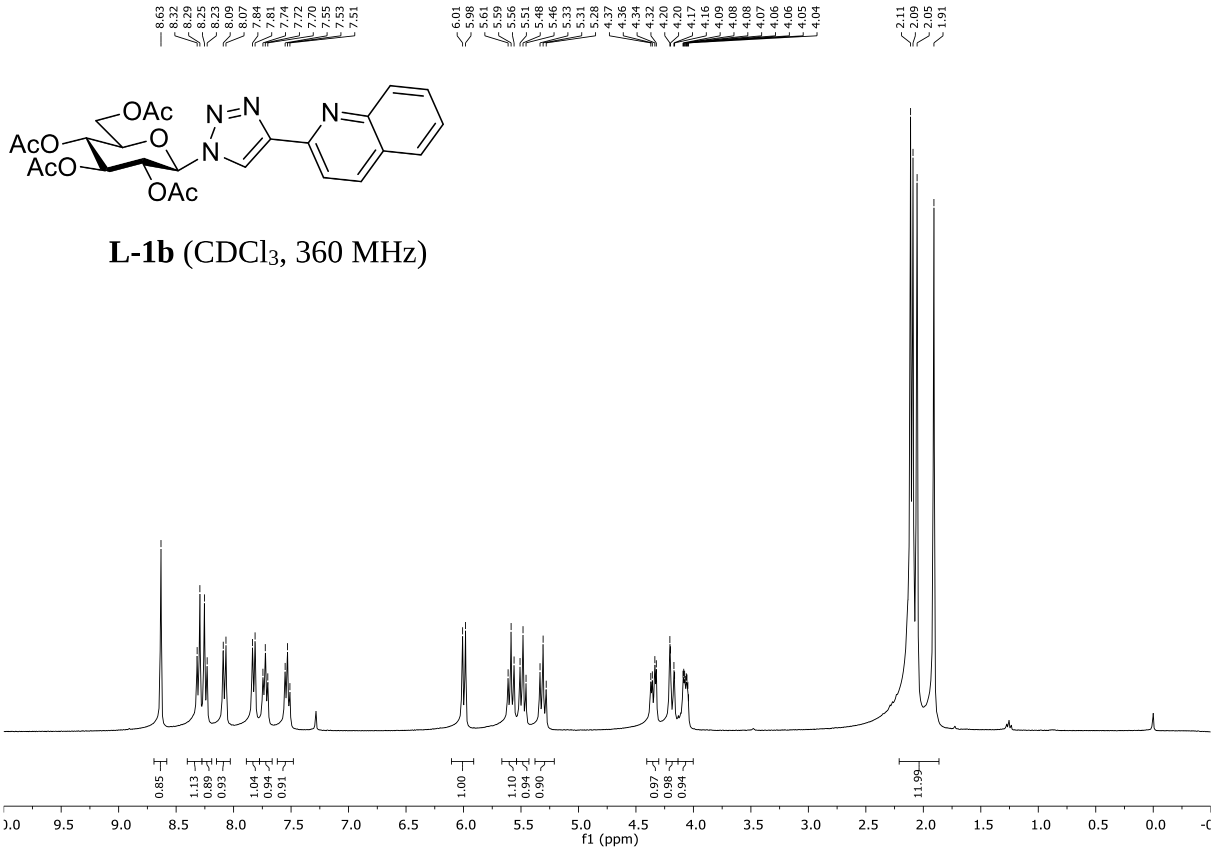

L-1b ($CDCl_3$, 360 MHz)

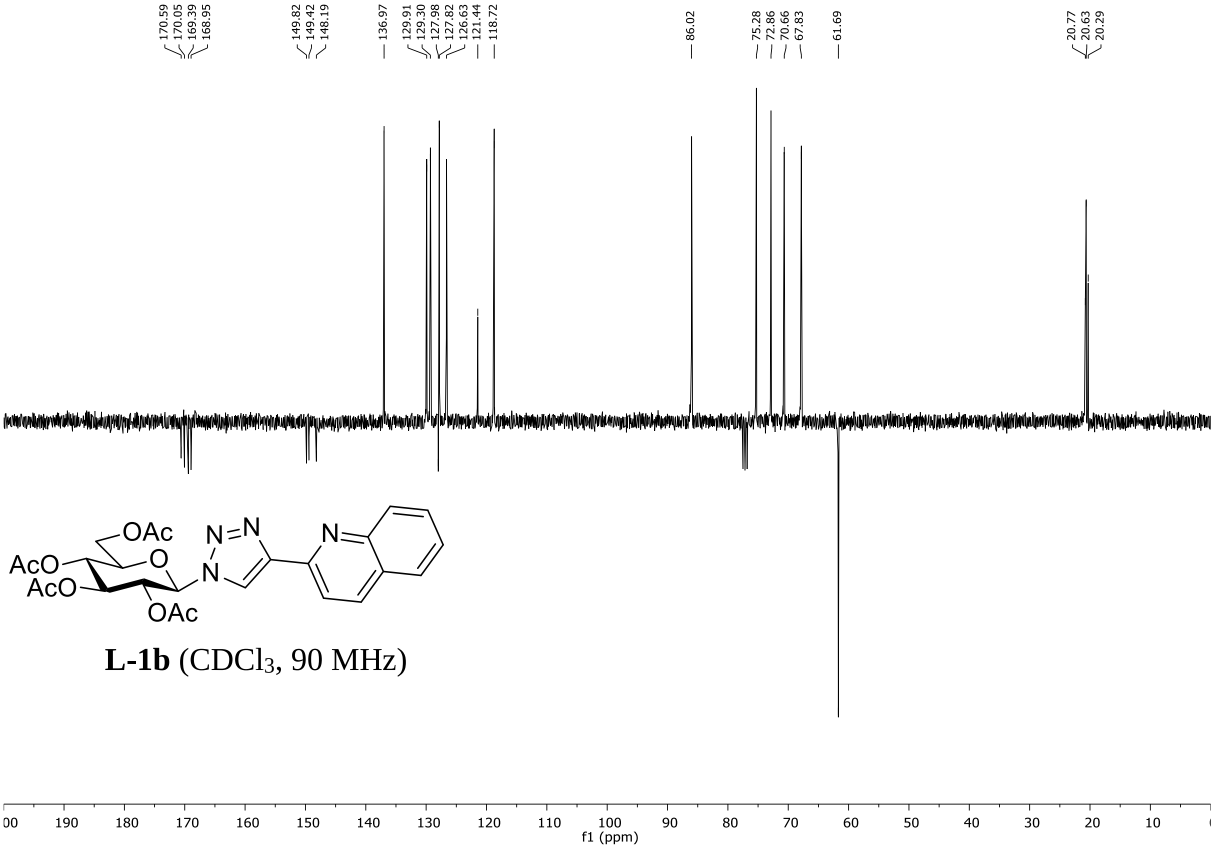

L-1b ($CDCl_3$, 90 MHz)

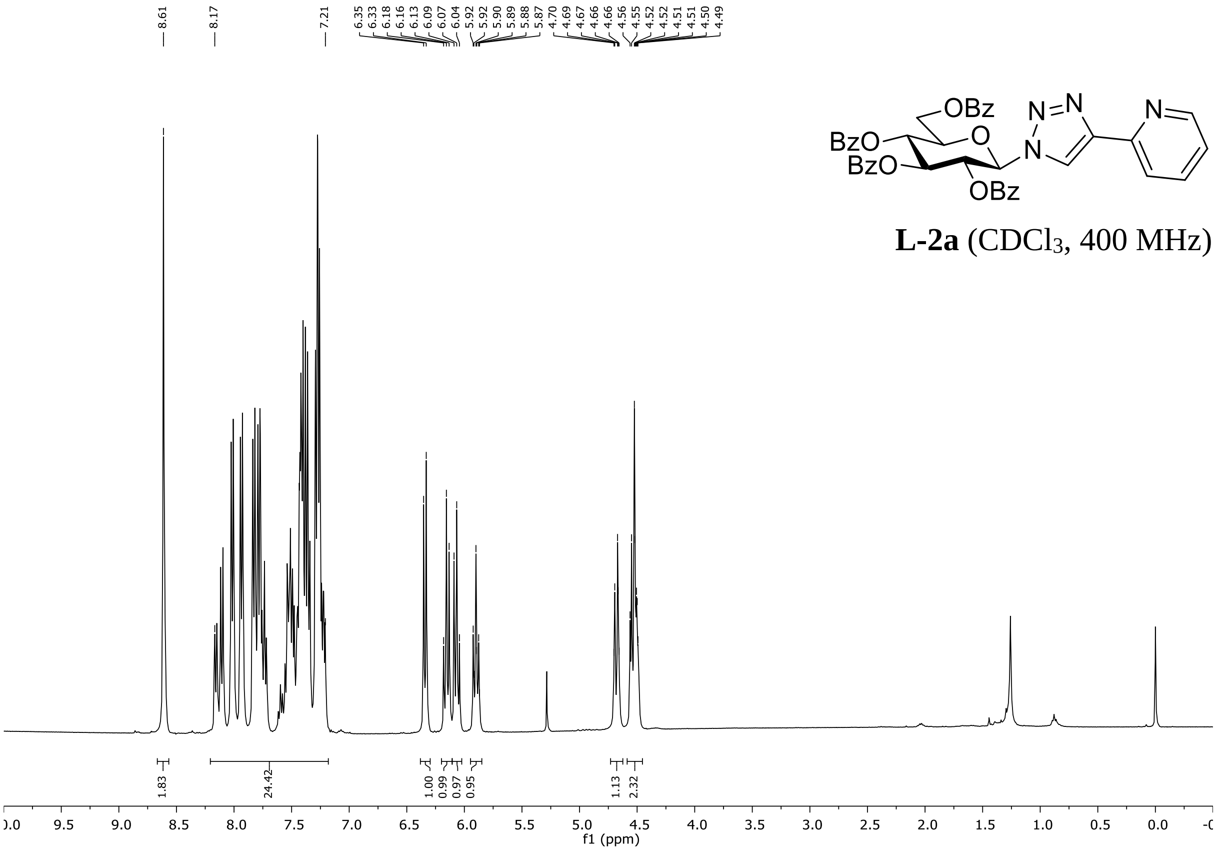

L-2a ($CDCl_3$, 400 MHz)

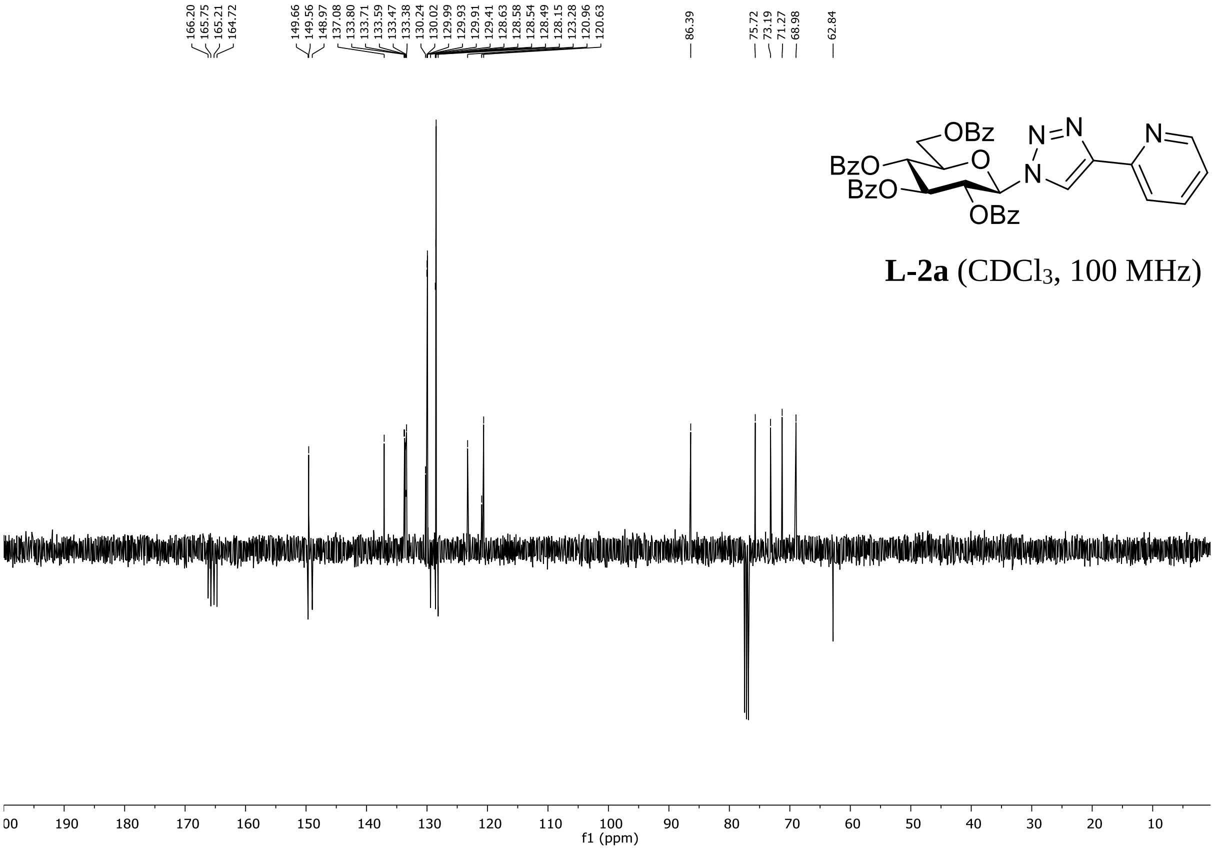

L-2a ($CDCl_3$, 100 MHz)

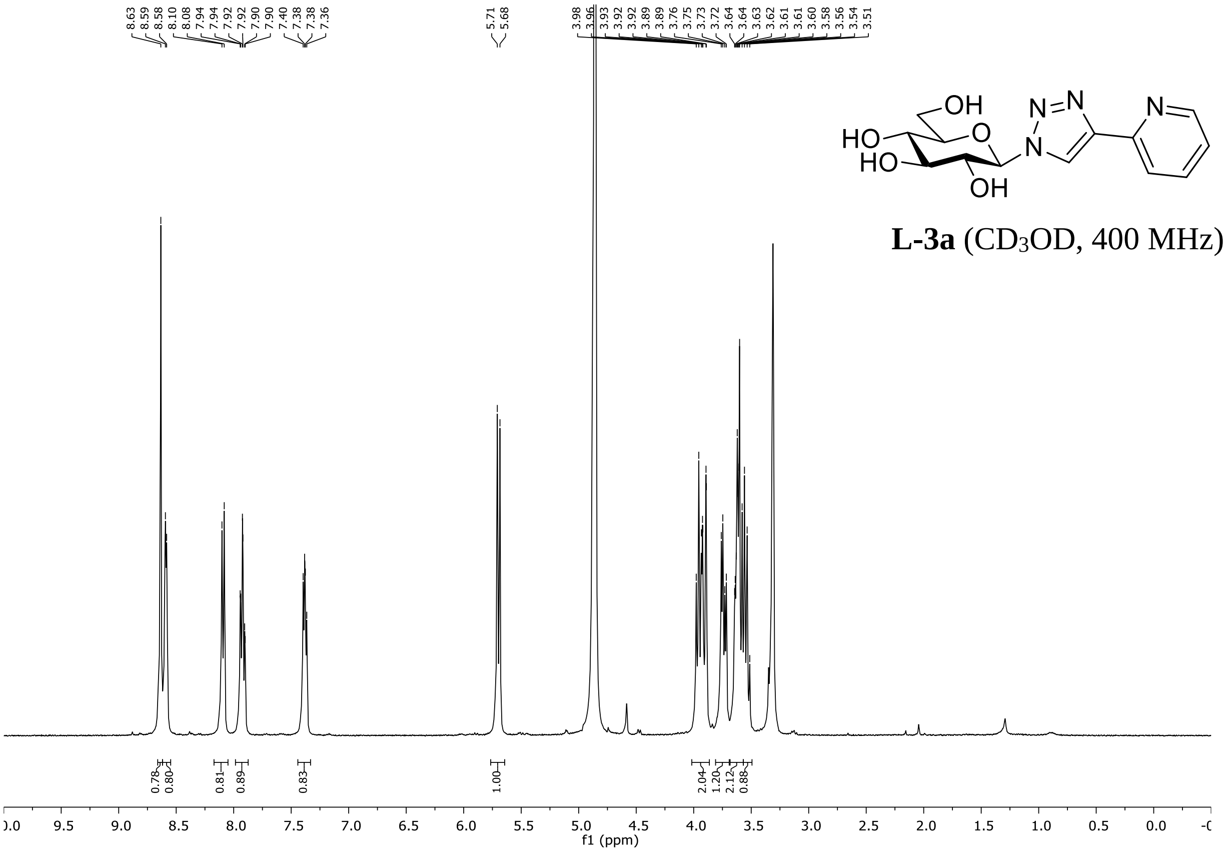

L-3a (CD_3OD, 400 MHz)

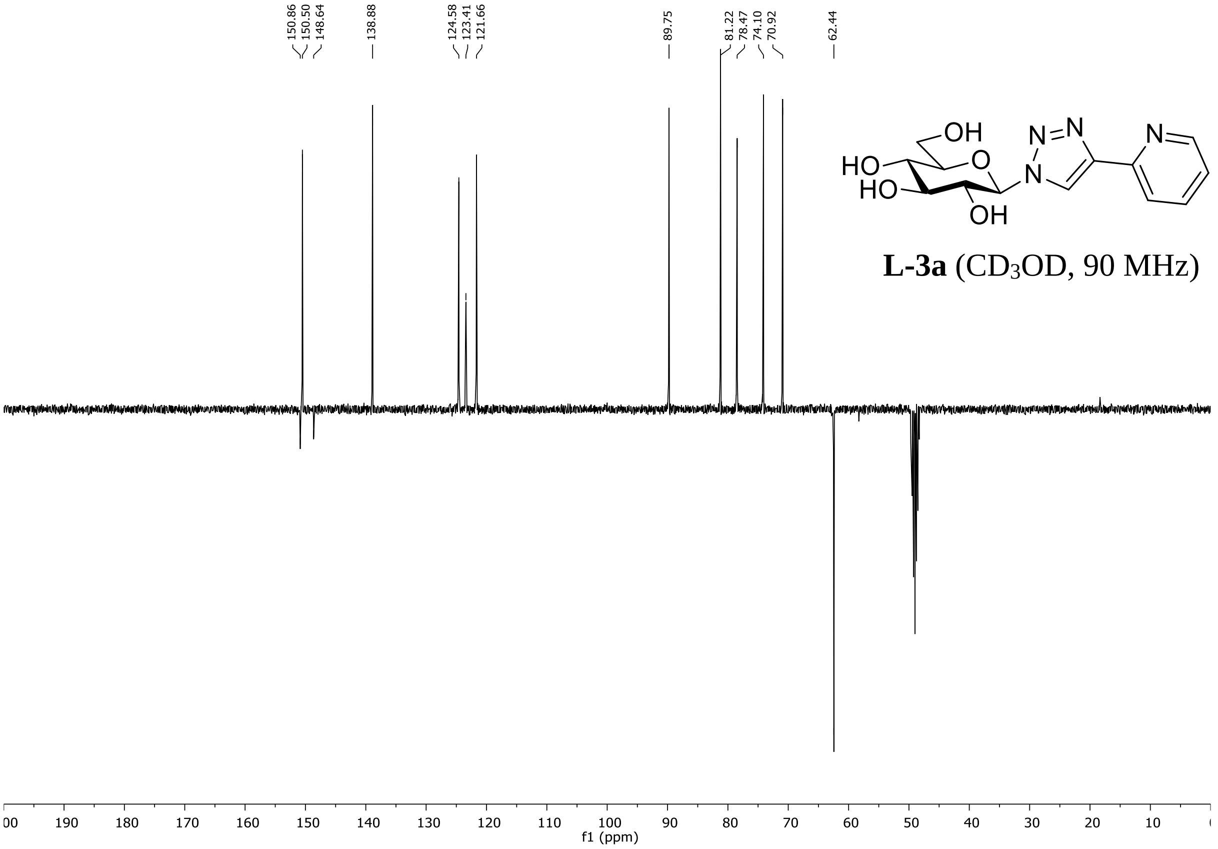

L-3a (CD_3OD, 90 MHz)

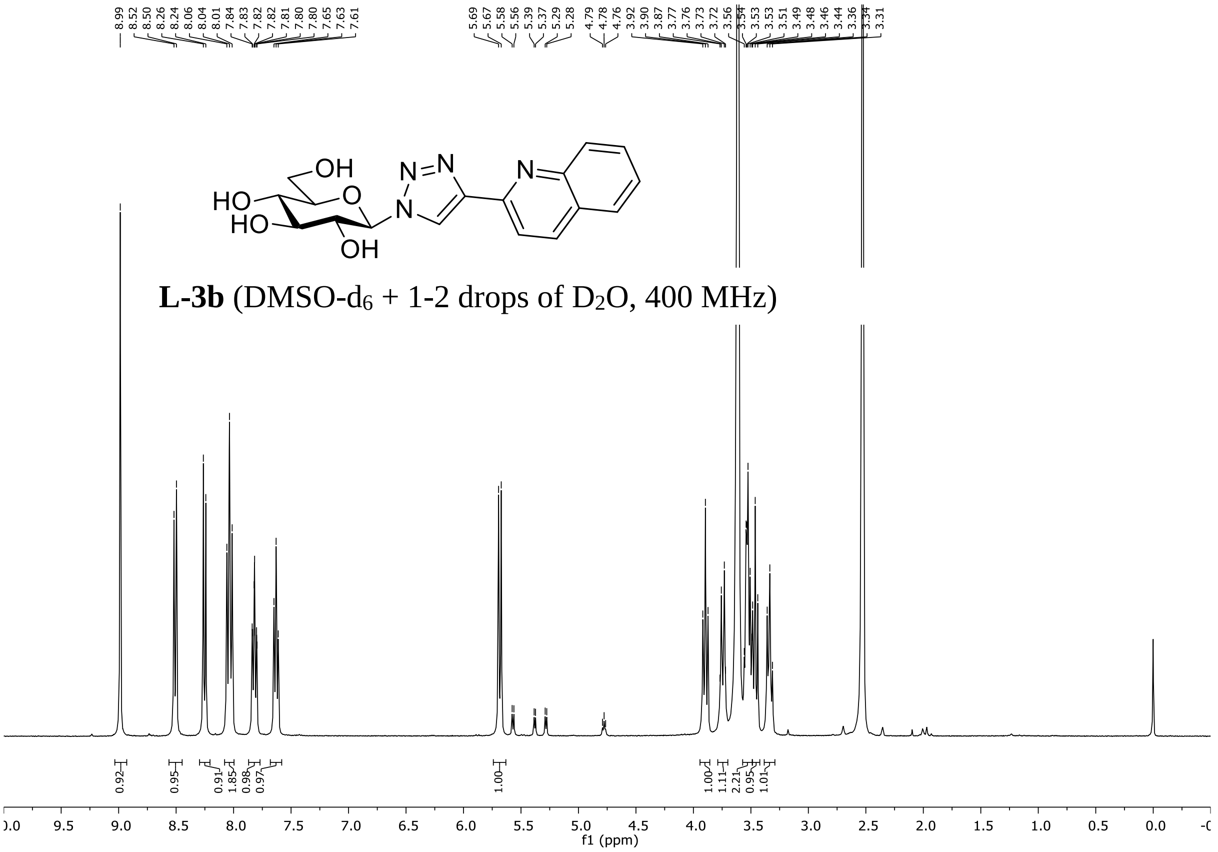

L-3b (DMSO-d_6 + 1-2 drops of D_2O, 400 MHz)

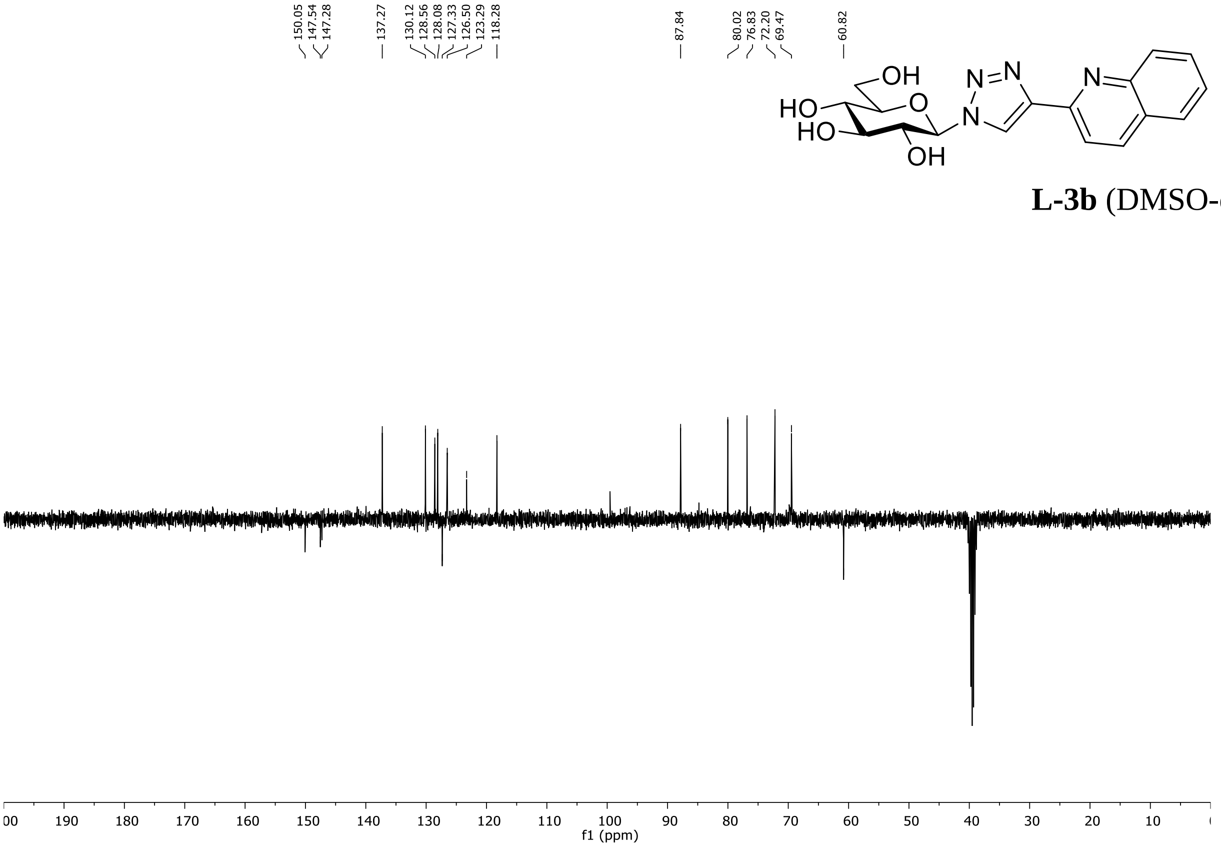

L-3b (DMSO-d_6, 90 MHz)

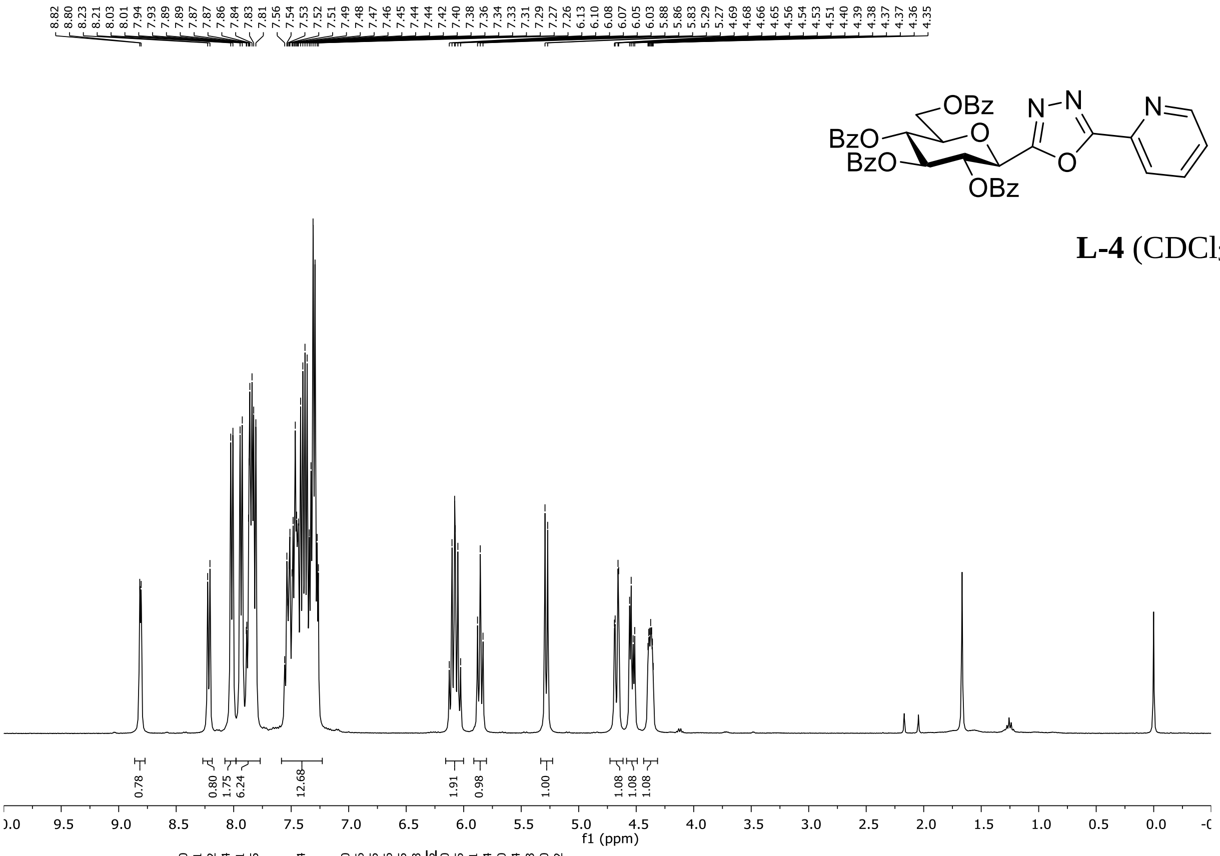

L-4 (CDCl$_3$, 400 MHz)

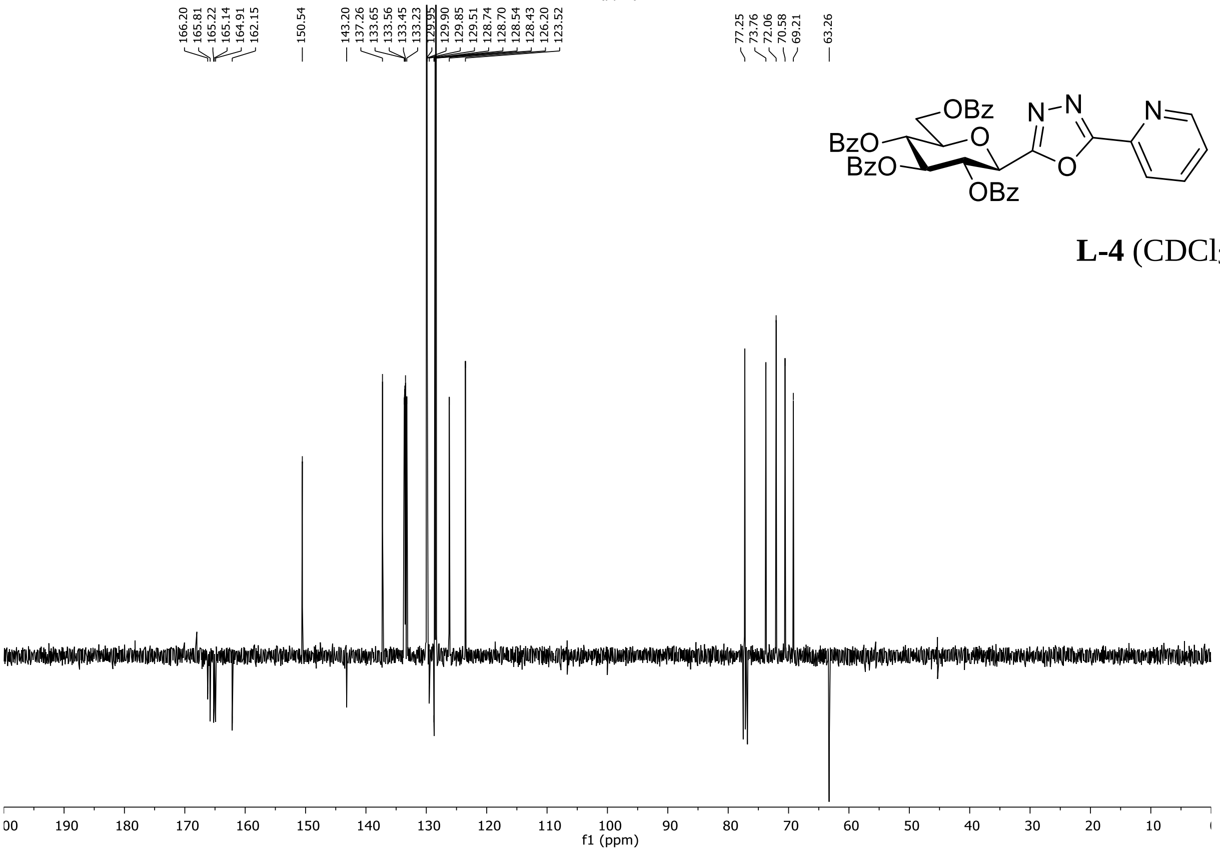

L-4 (CDCl$_3$, 90 MHz)

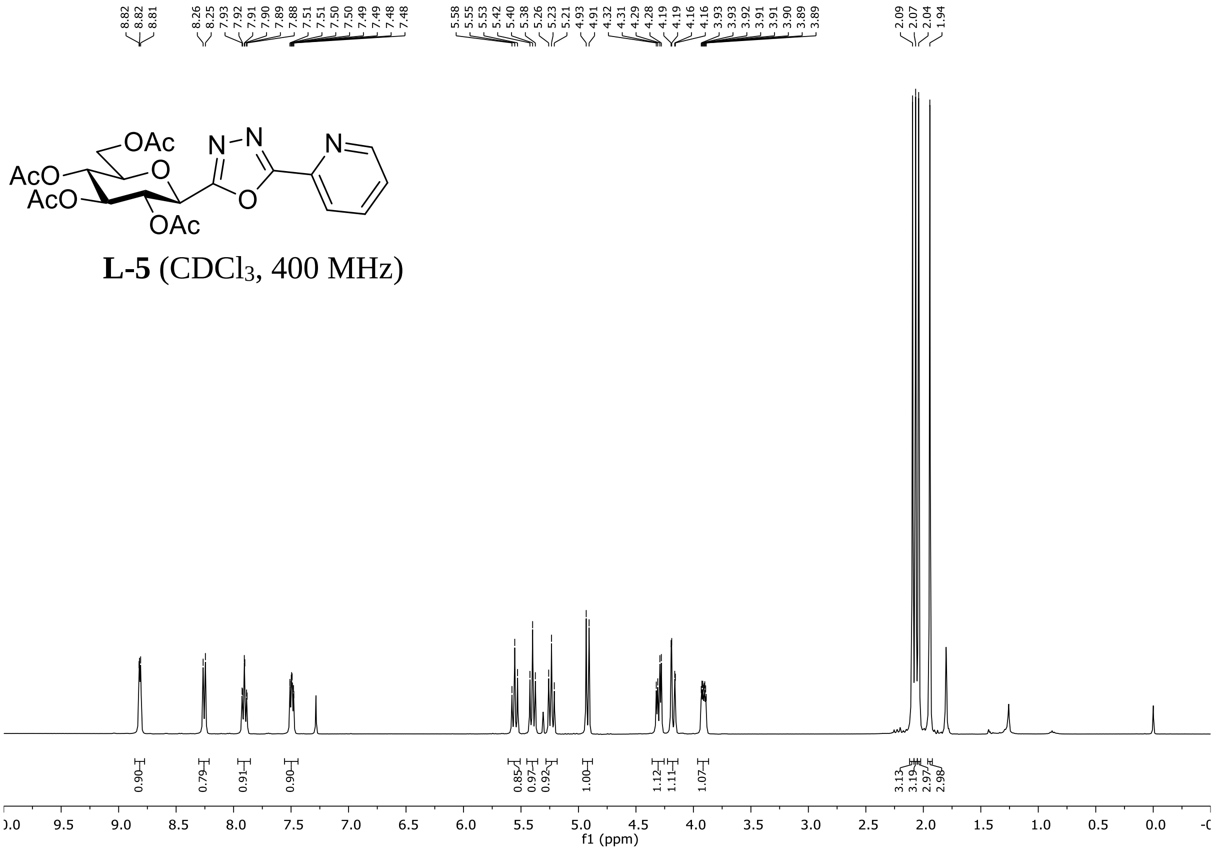

8.82 8.82 8.81 8.26 8.25 7.93 7.92 7.91 7.90 7.89 7.88 7.51 7.51 7.50 7.50 7.49 7.49 7.48 7.48
5.58 5.55 5.53 5.42 5.40 5.38 5.26 5.23 5.21 4.93 4.91 4.32 4.31 4.29 4.28 4.19 4.19 4.16 4.16 3.93 3.93 3.92 3.91 3.91 3.90 3.89 3.89
2.09 2.07 2.04 1.94
AcO
AcO
OAc
OAc
O
N
N
O
N
L-5 (CDCl3, 400 MHz)
0.90 0.79 0.91 0.90 0.85 0.97 0.92 1.00 1.12 1.11 1.07 3.13 3.19 2.97 2.98
f1 (ppm)

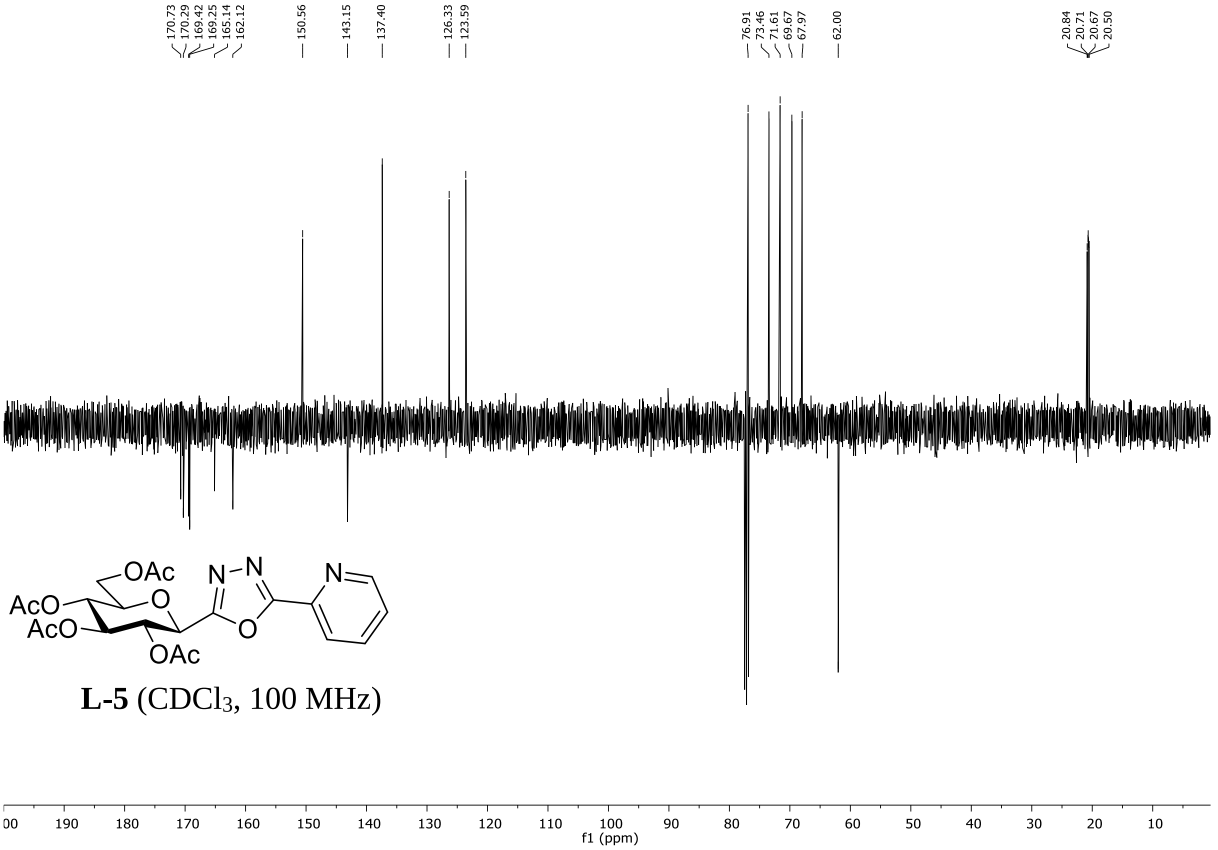

170.73 170.29 169.42 169.25 165.14 162.12 150.56 143.15 137.40 126.33 123.59
76.91 73.46 71.61 69.67 67.97 62.00
20.84 20.71 20.67 20.50
AcO
AcO
OAc
OAc
O
N
N
O
N
L-5 (CDCl3, 100 MHz)
f1 (ppm)

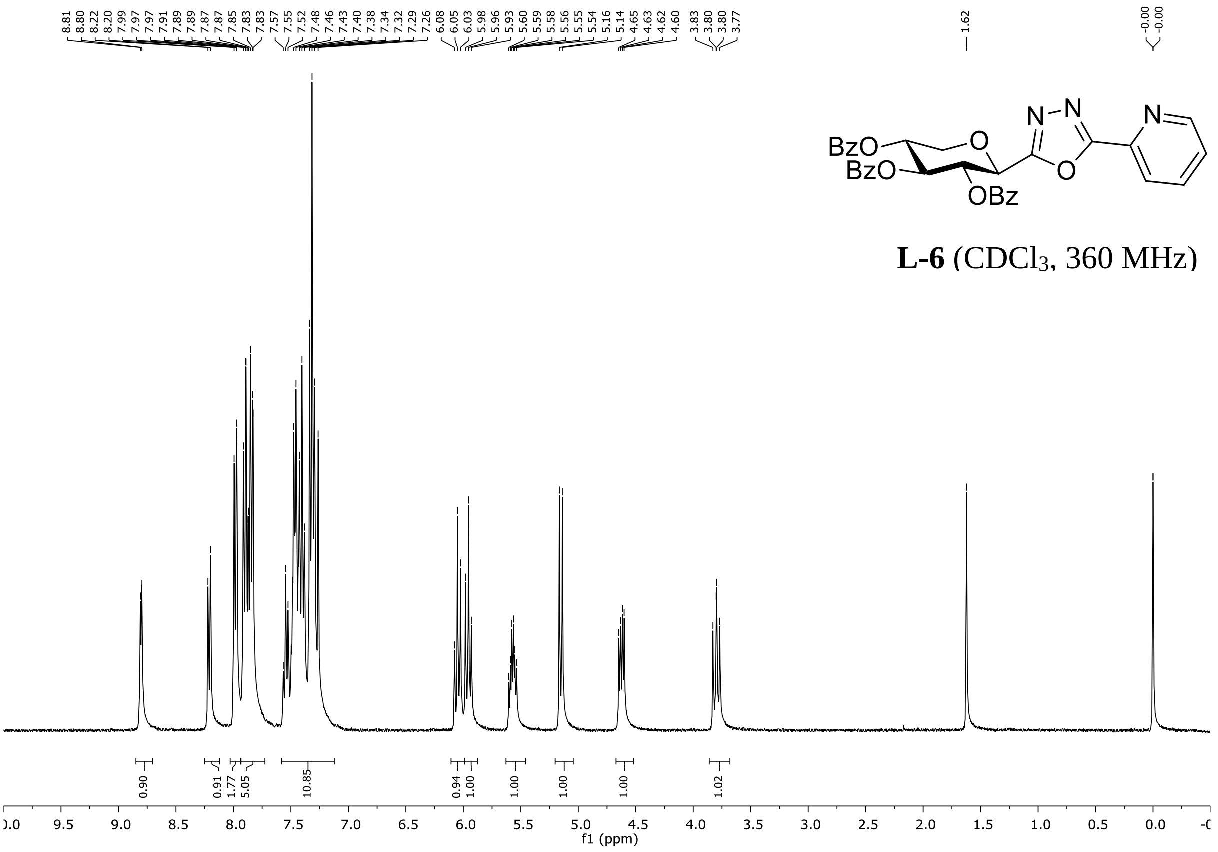

L-6 (CDCl$_3$, 360 MHz)

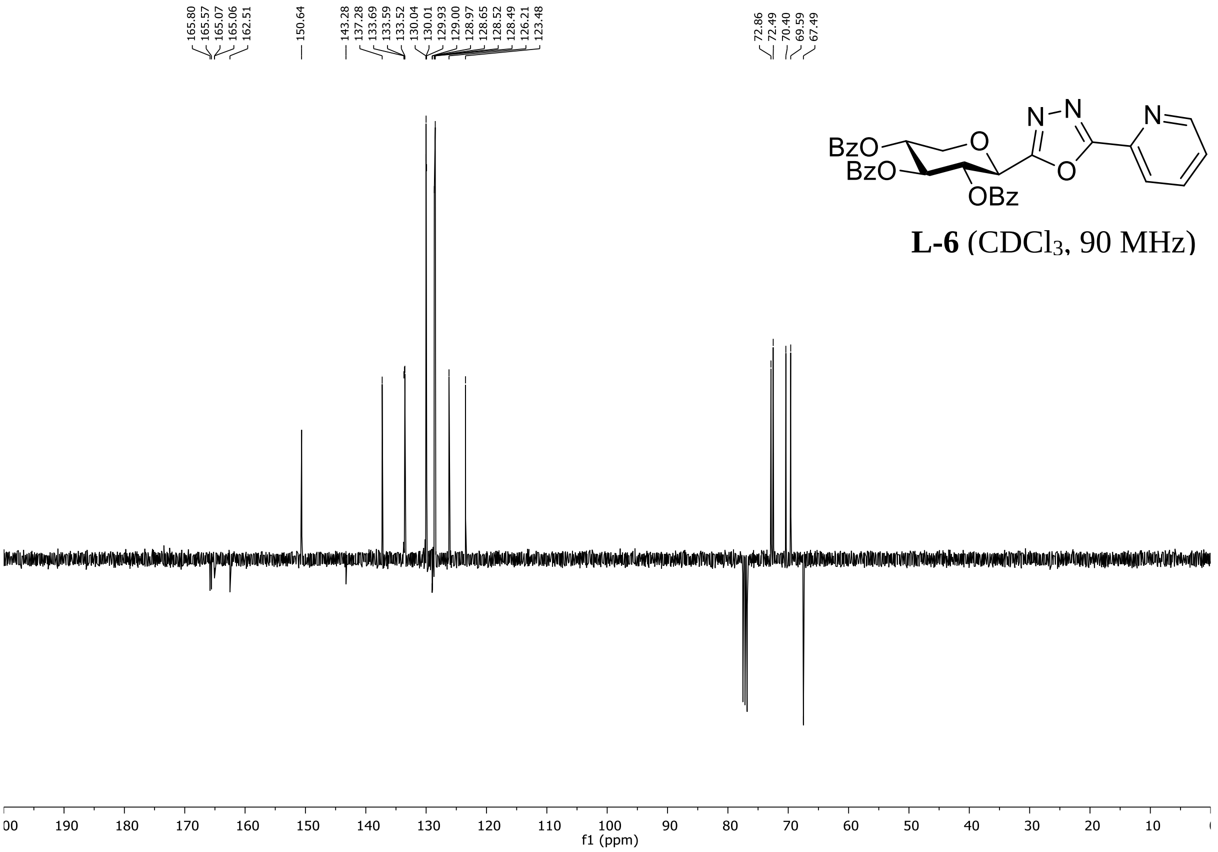

L-6 (CDCl$_3$, 90 MHz)

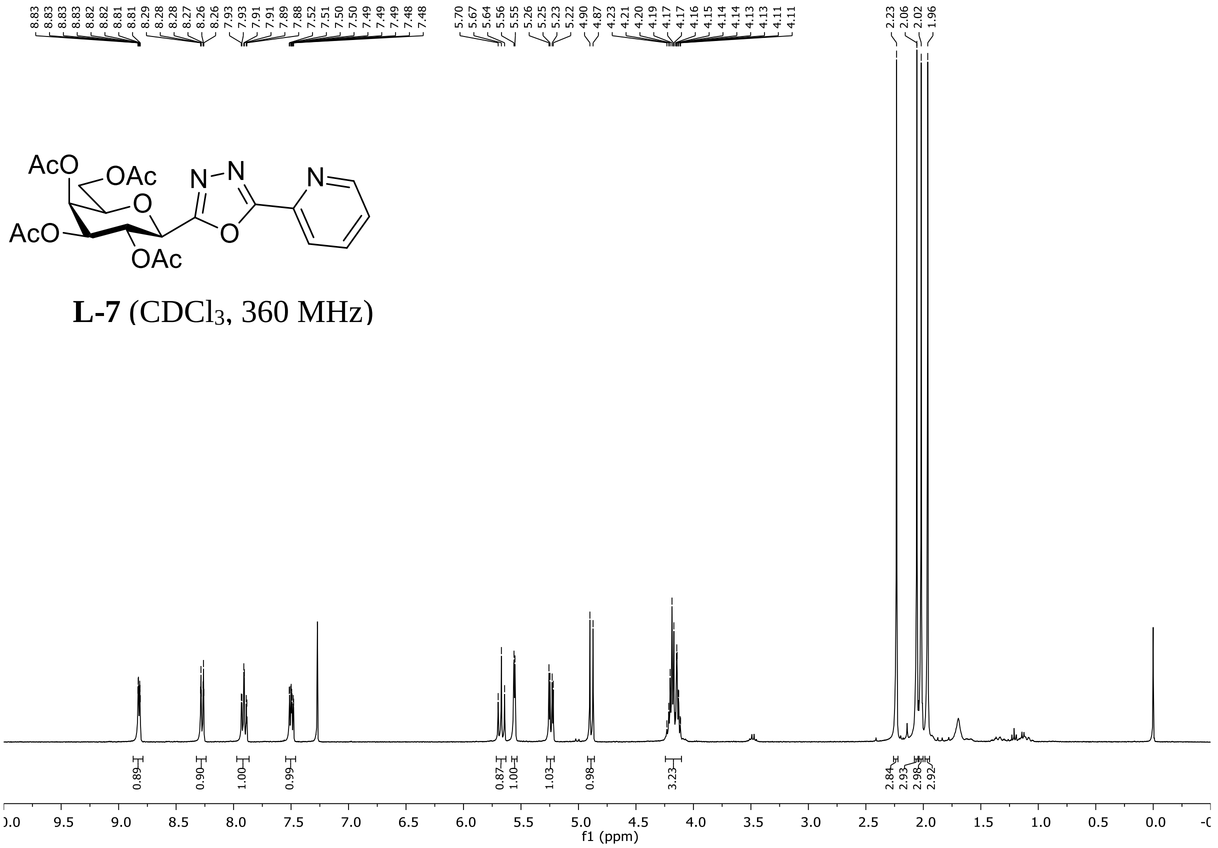

L-7 (CDCl$_3$, 360 MHz)

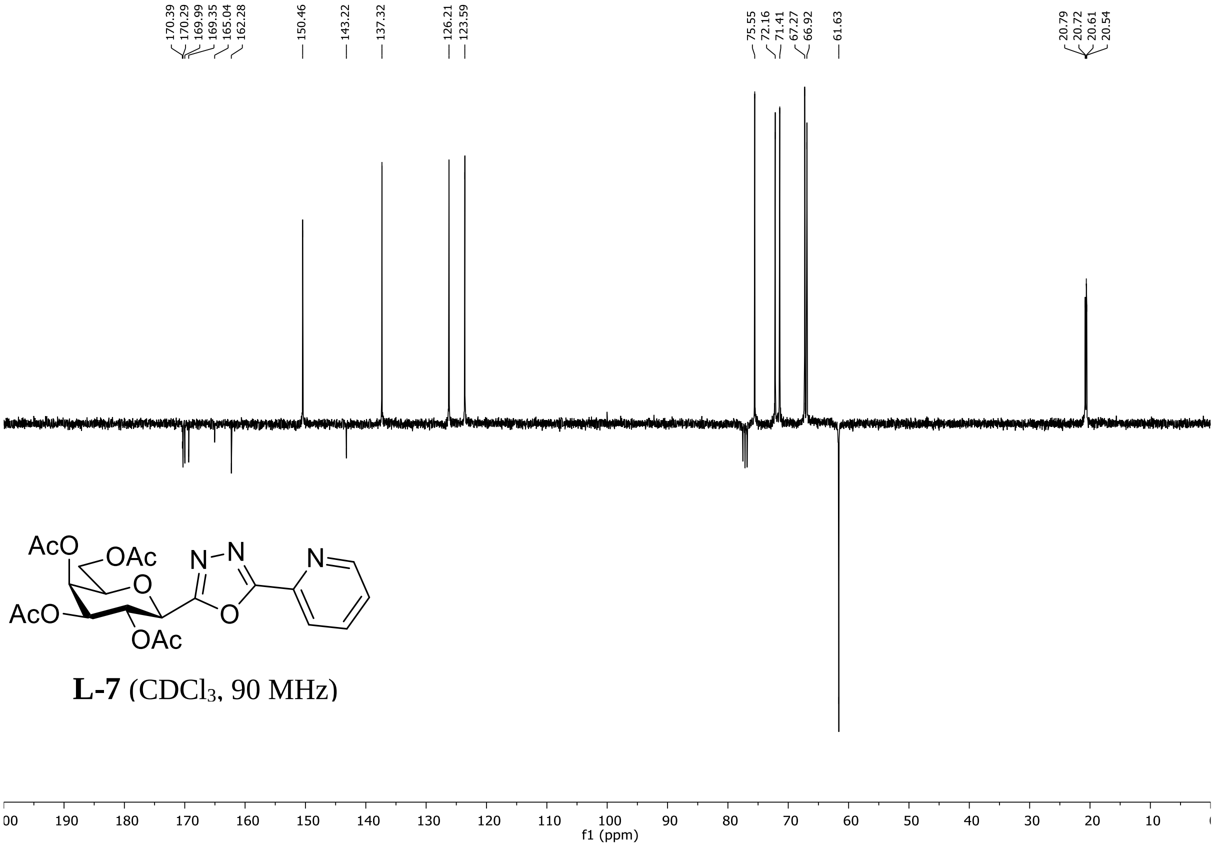

L-7 (CDCl$_3$, 90 MHz)

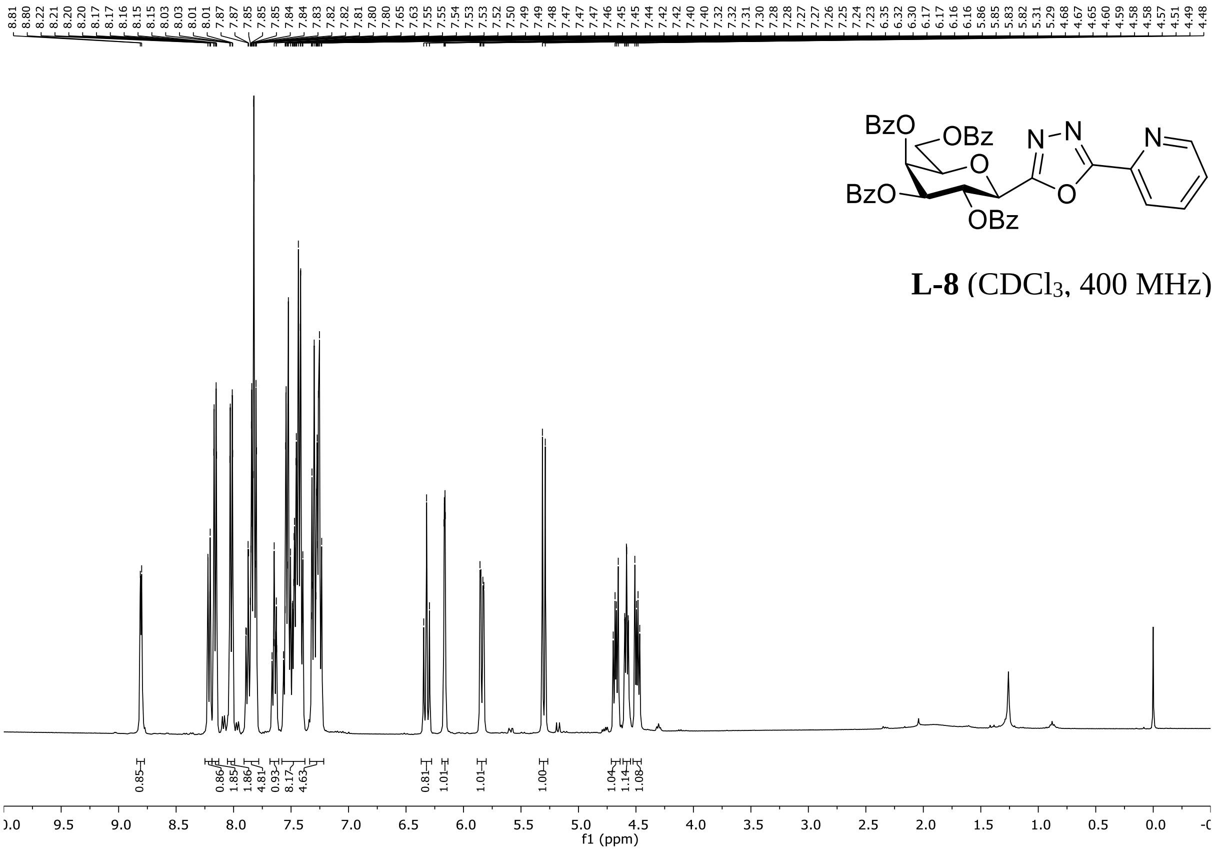

L-8 ($CDCl_3$, 400 MHz)

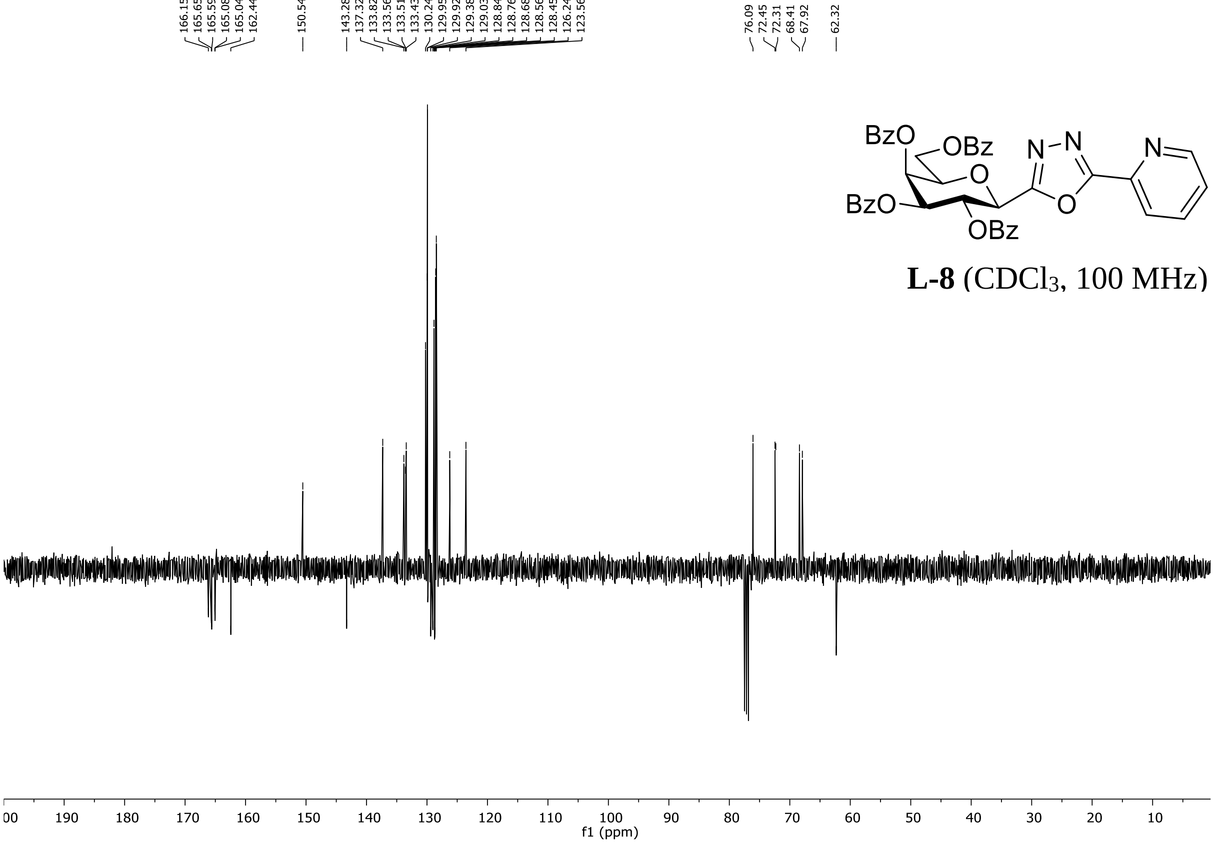

L-8 ($CDCl_3$, 100 MHz)

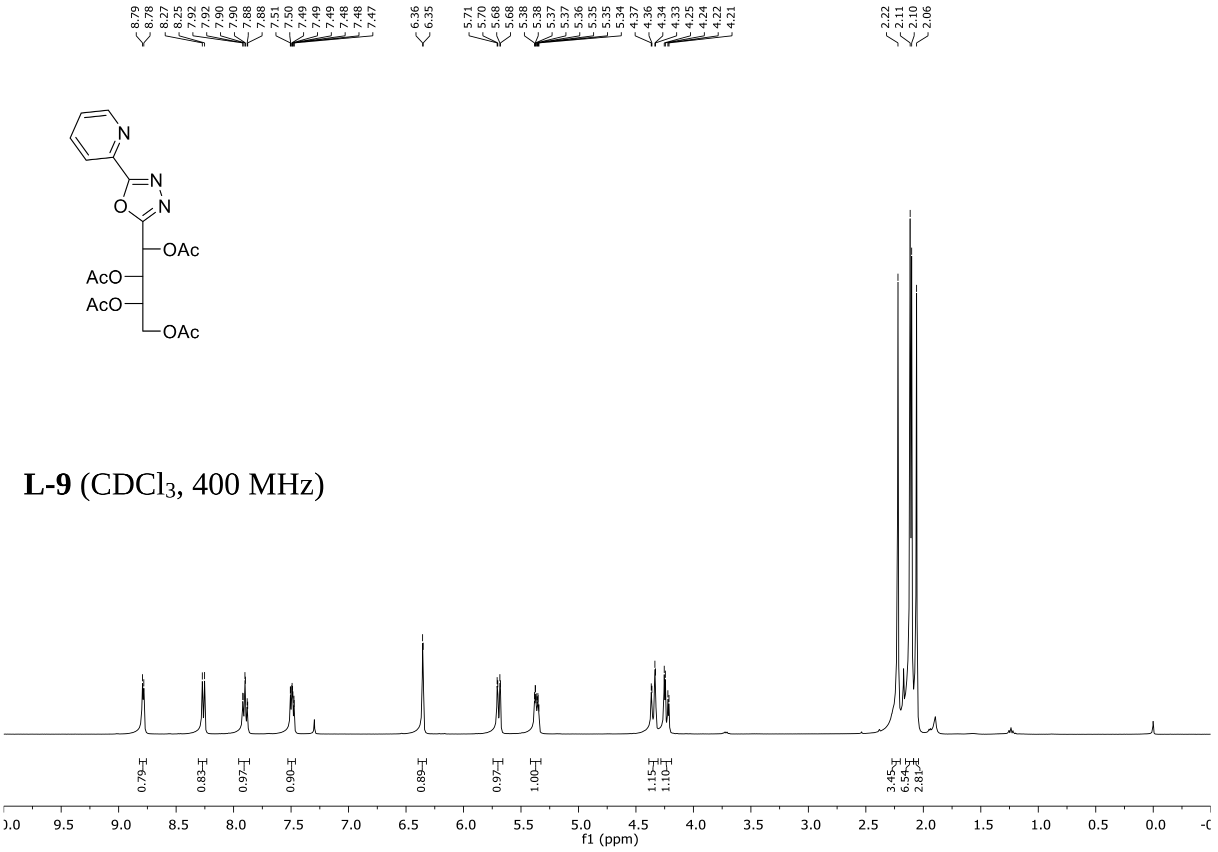

L-9 ($CDCl_3$, 400 MHz)

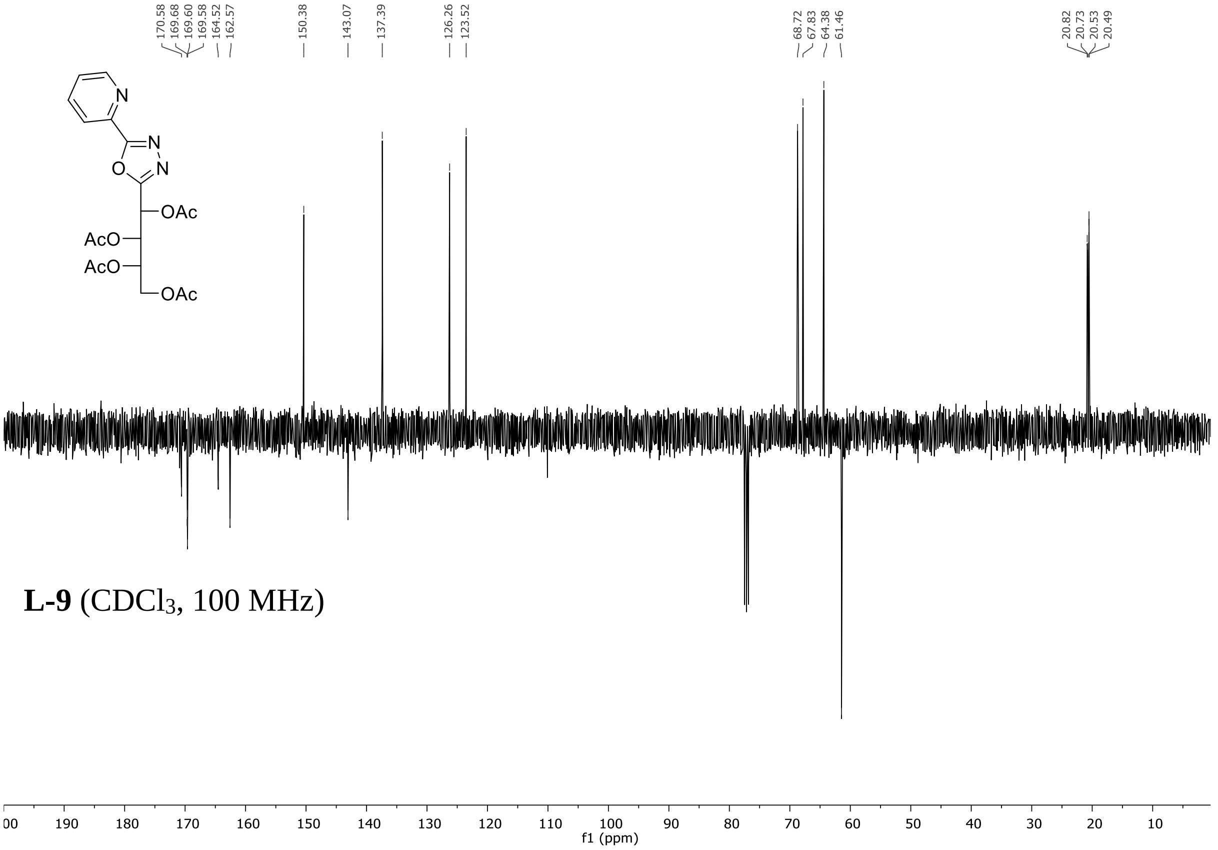

L-9 ($CDCl_3$, 100 MHz)

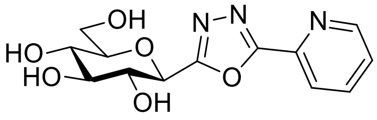

L-10 (CD3OD, 400 MHz)

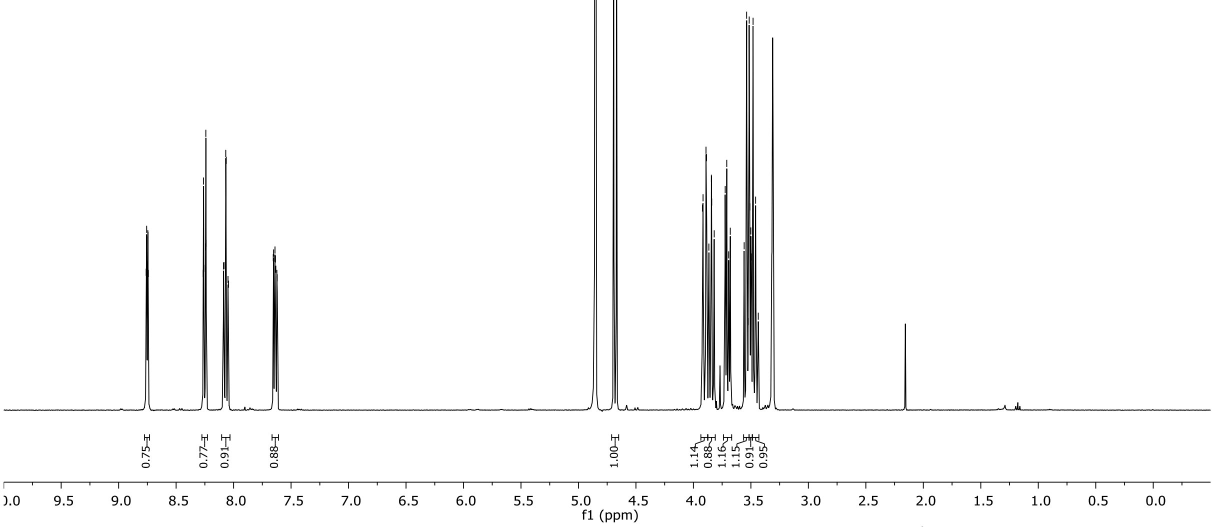

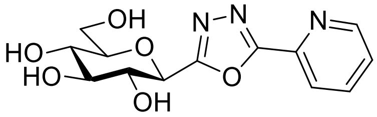

L-10 (CD3OD, 90 MHz)

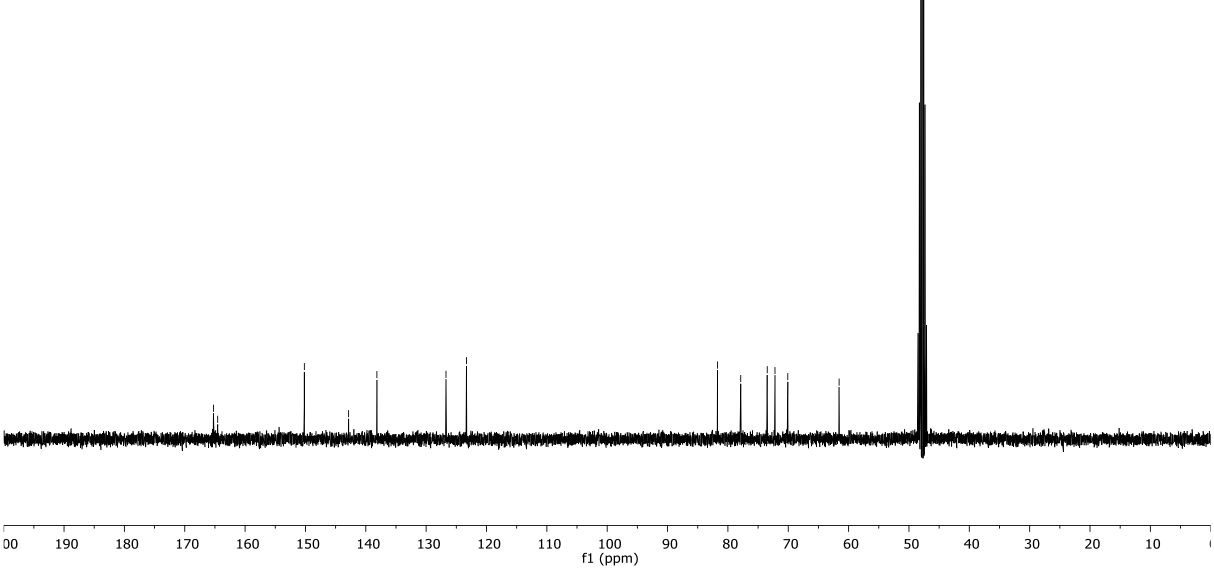

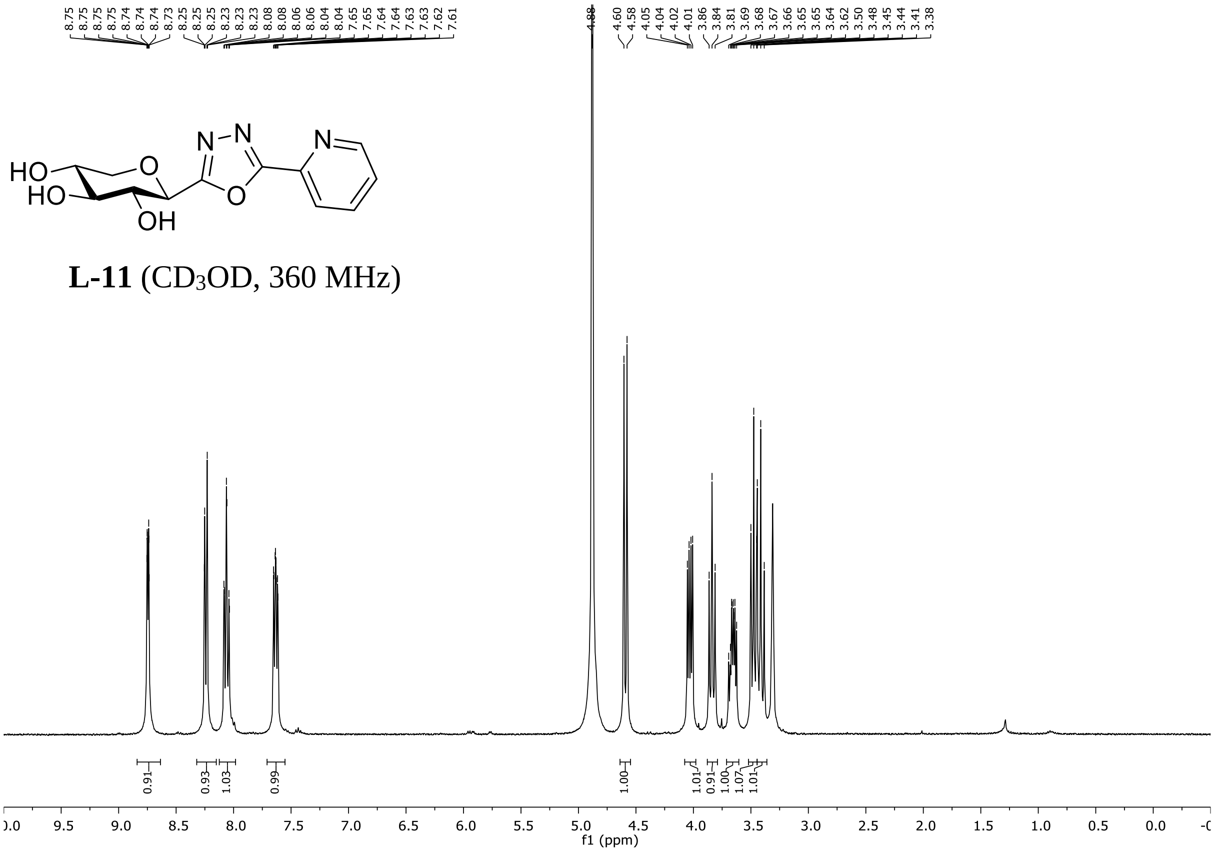

L-11 (CD$_3$OD, 360 MHz)

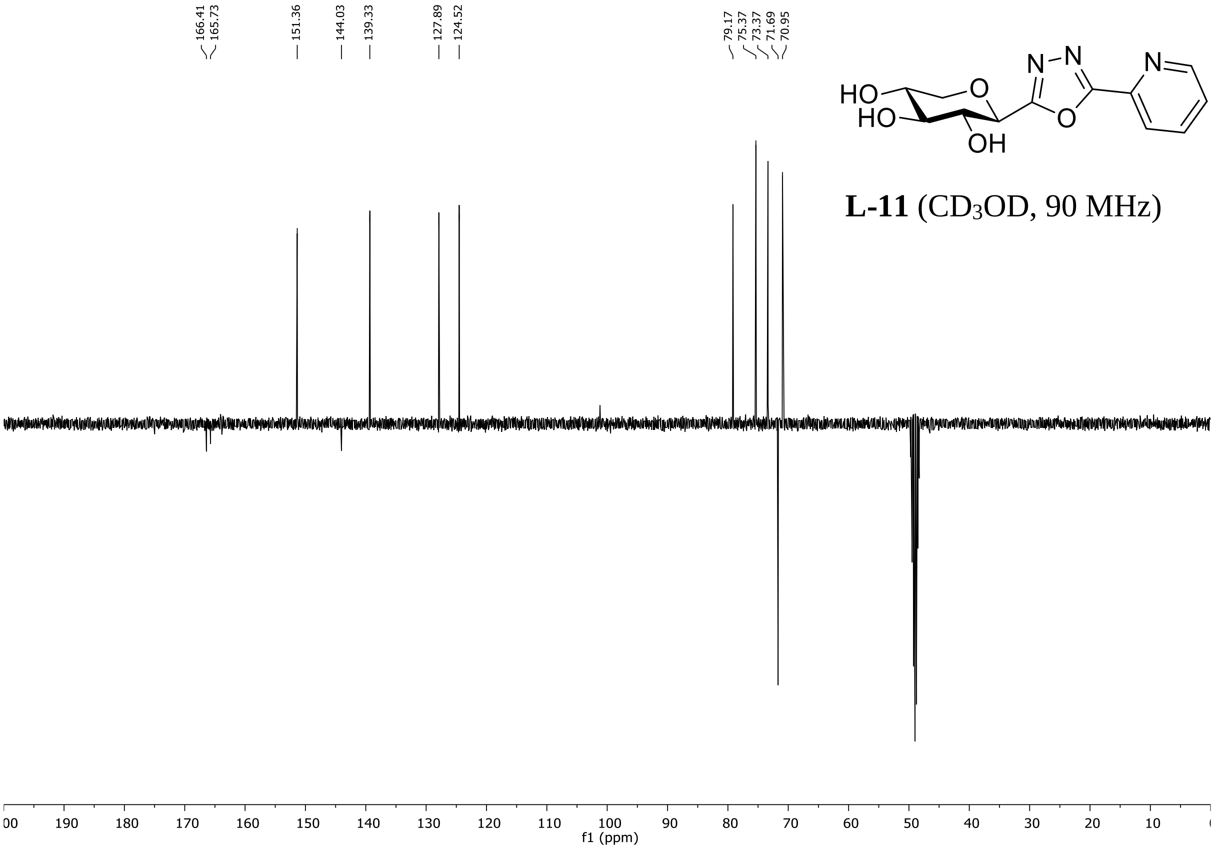

L-11 (CD$_3$OD, 90 MHz)

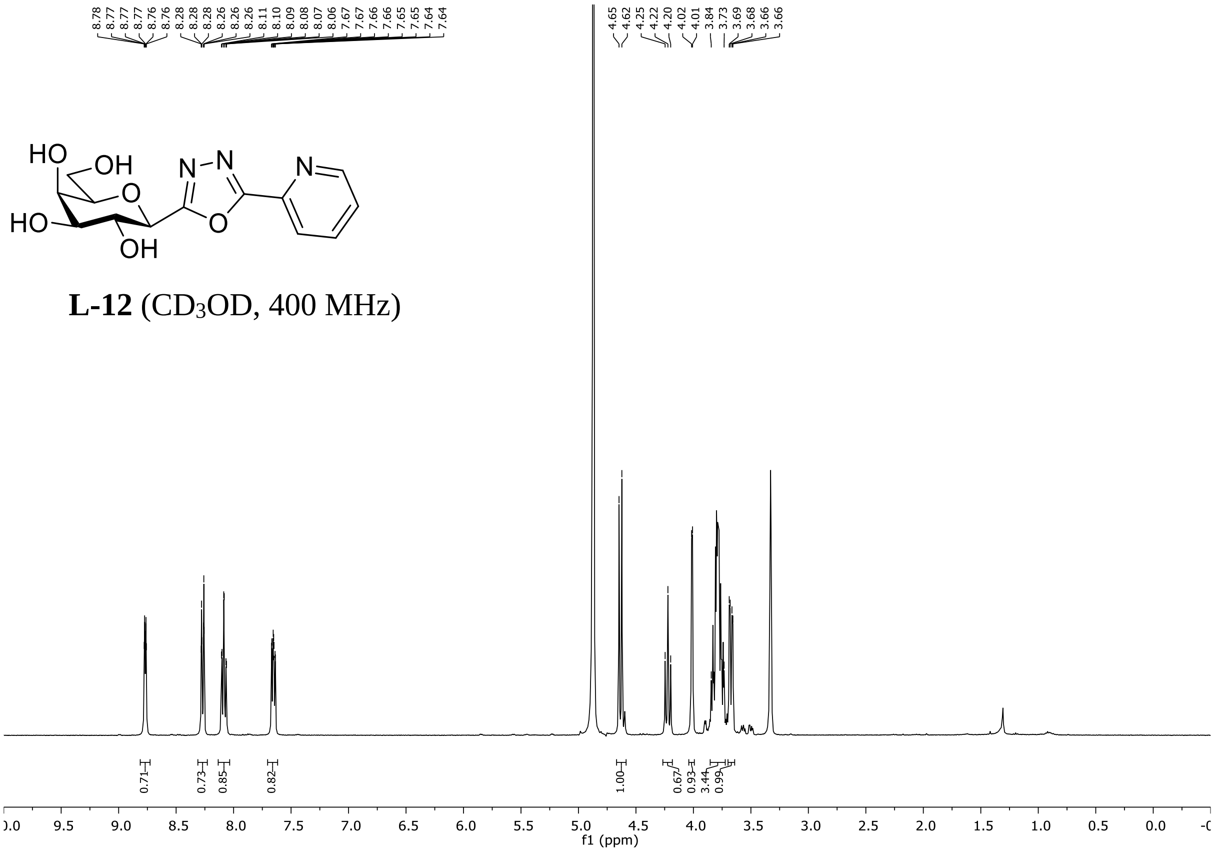

L-12 (CD$_3$OD, 400 MHz)

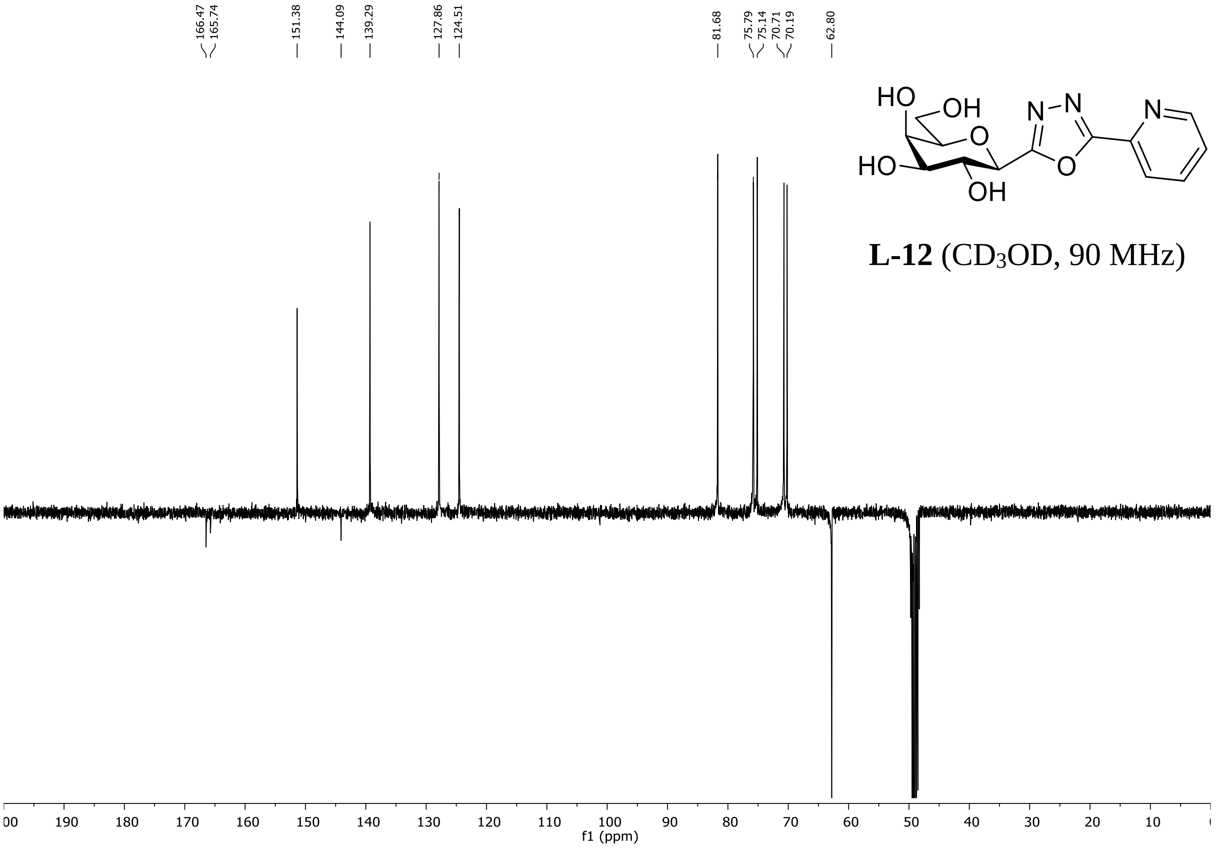

L-12 (CD$_3$OD, 90 MHz)

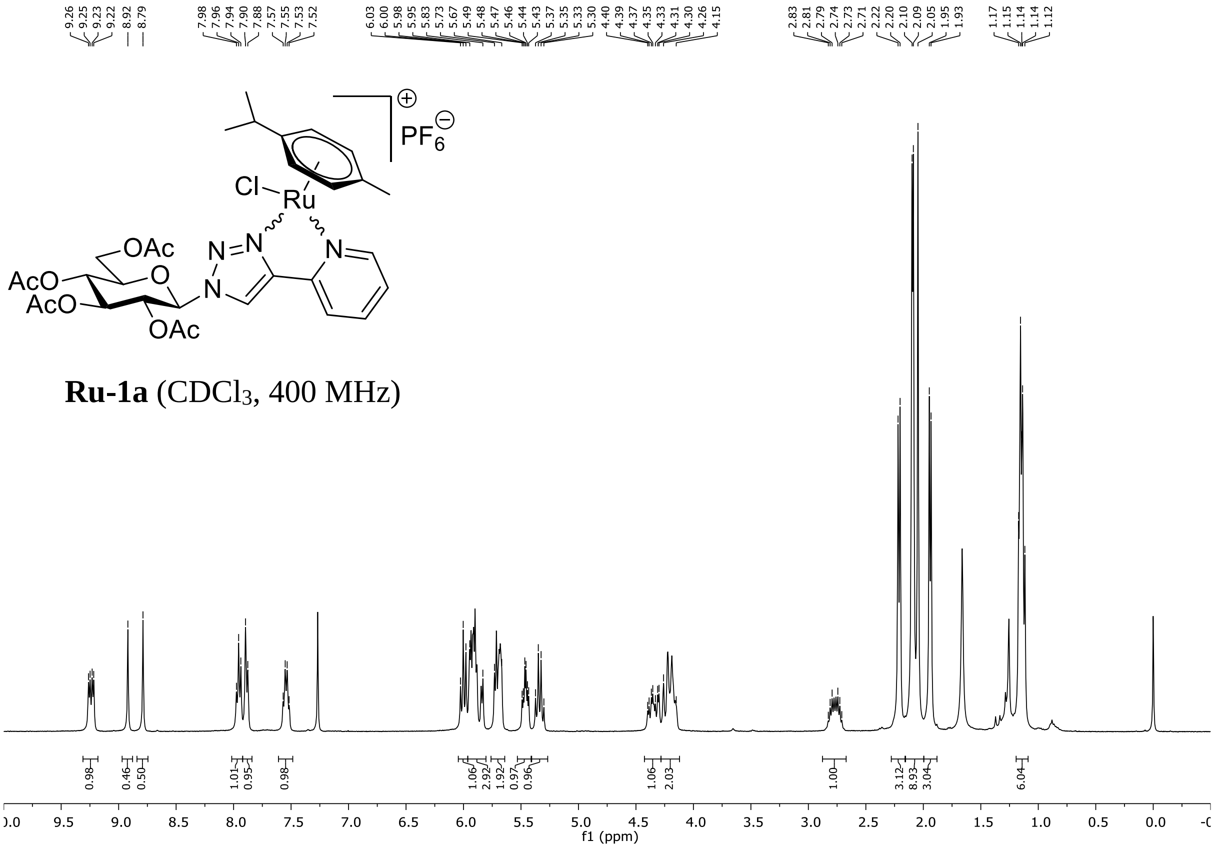

Ru-1a ($CDCl_3$, 400 MHz)

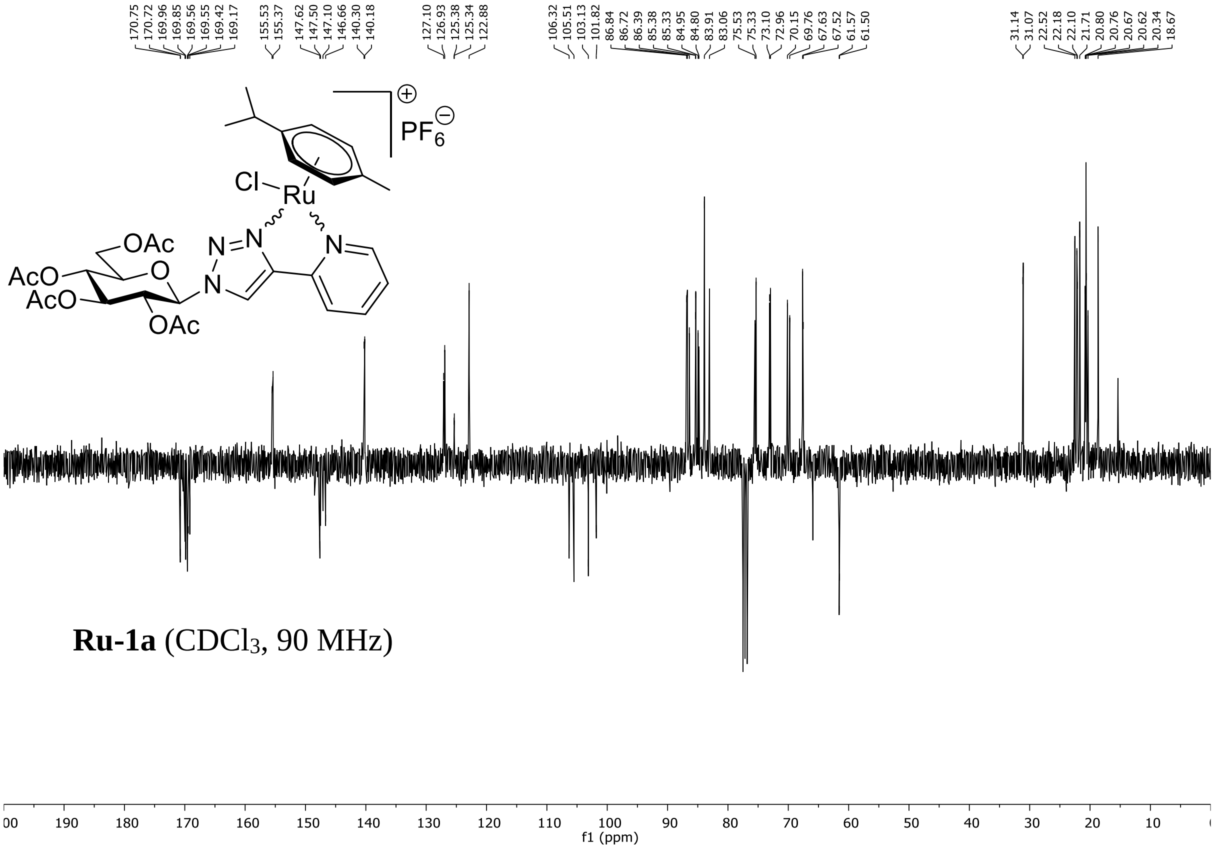

Ru-1a ($CDCl_3$, 90 MHz)

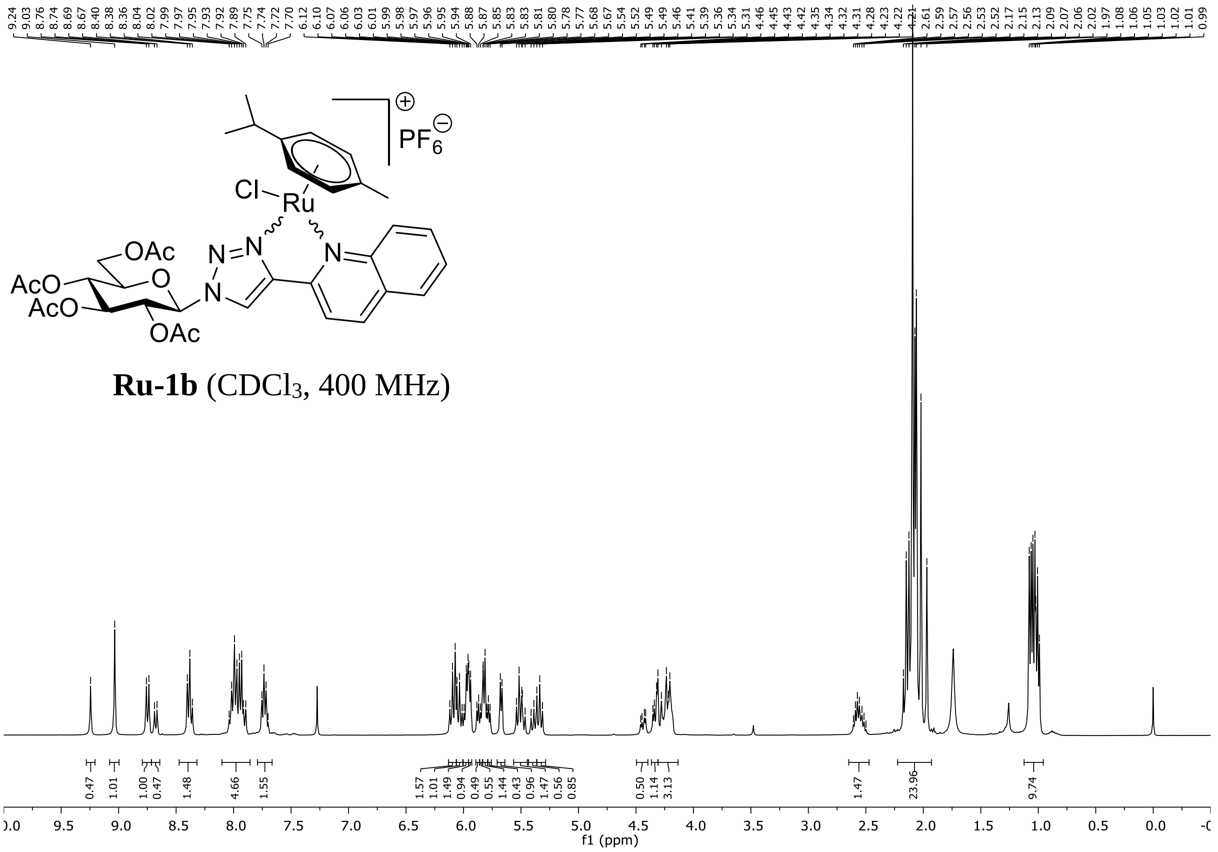

Ru-1b ($CDCl_3$, 400 MHz)

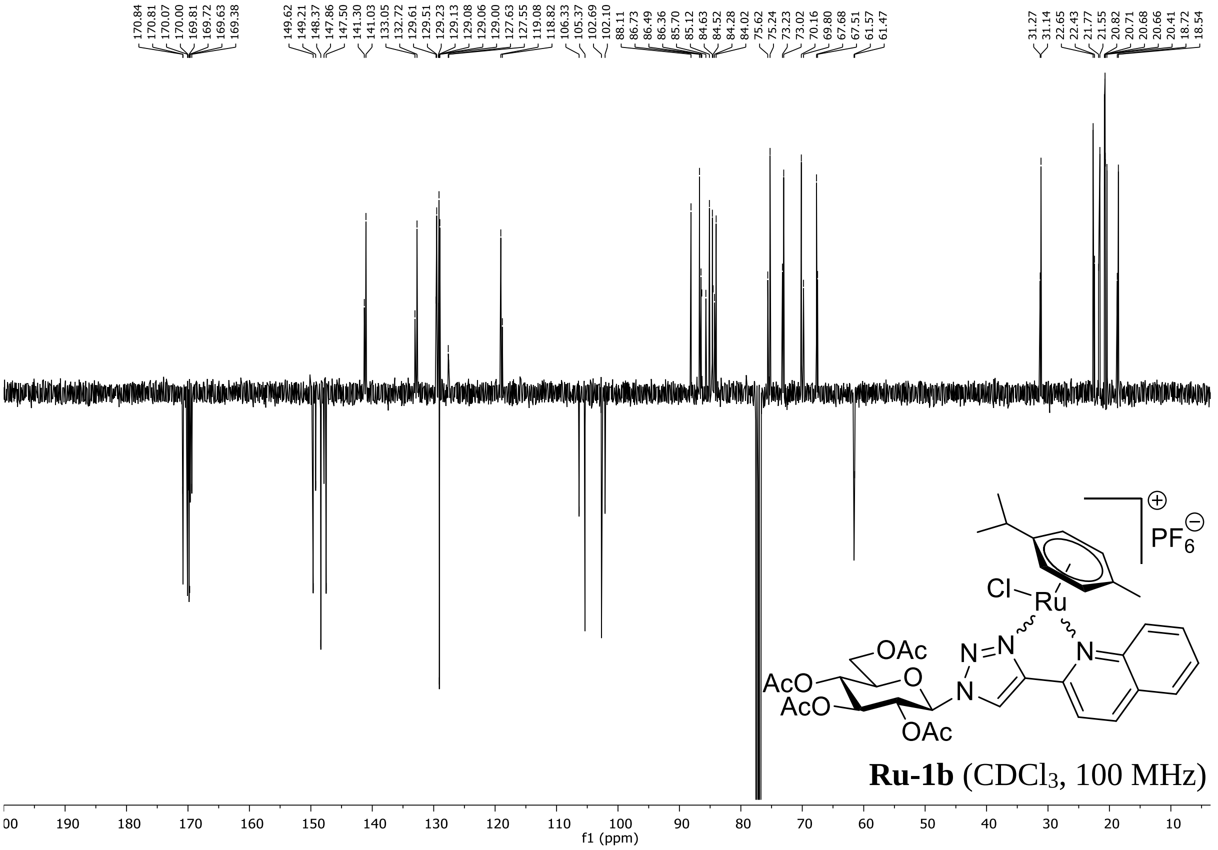

Ru-1b ($CDCl_3$, 100 MHz)

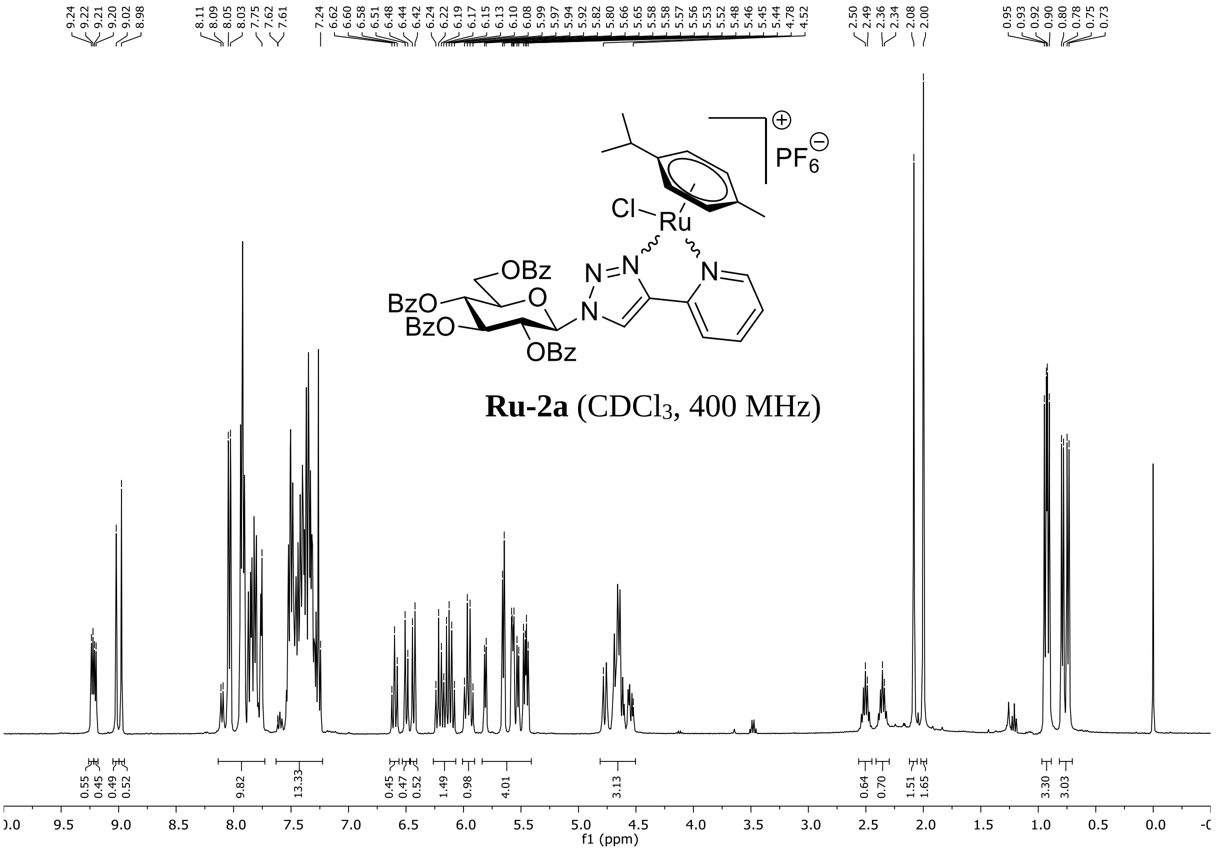
9.24 9.22 9.21 9.20 9.02 8.98
8.11 8.09 8.05 8.03 7.75 7.62 7.61
7.24 6.62 6.60 6.58 6.51 6.48 6.44 6.42 6.24 6.22 6.19 6.17 6.15 6.13 6.10 6.08 5.99 5.97 5.94 5.92 5.82 5.80 5.66 5.65 5.58 5.58 5.57 5.56 5.53 5.52 5.48 5.46 5.45 5.44 4.78 4.52
2.50 2.49 2.36 2.34 2.08 2.00
0.95 0.93 0.92 0.90 0.80 0.78 0.75 0.73
PF6
Cl
Ru
N
OBz
BzO
BzO
OBz
O
Ru-2a (CDCl3, 400 MHz)
0.55 0.45 0.49 0.52 9.82 13.33 0.45 0.47 0.52 1.49 0.98 4.01 3.13 0.64 0.70 1.51 1.65 3.30 3.03
9.5 9.0 8.5 8.0 7.5 7.0 6.5 6.0 5.5 5.0 4.5 4.0 3.5 3.0 2.5 2.0 1.5 1.0 0.5 0.0
f1 (ppm)

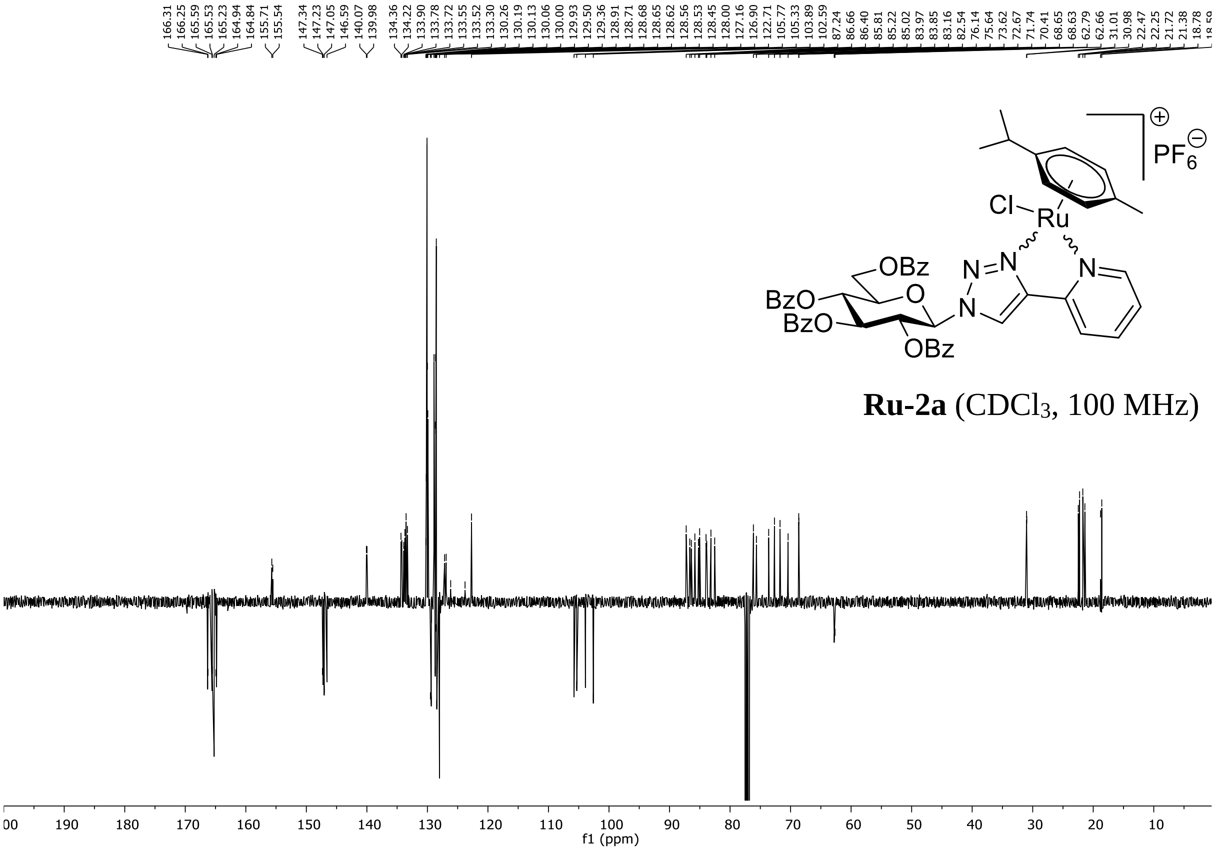
166.31 166.25 165.59 165.53 165.23 164.94 164.84 155.71 155.54 147.34 147.23 147.05 146.59 140.07 139.98 134.36 134.22 133.90 133.78 133.72 133.55 133.52 133.30 130.26 130.19 130.13 130.06 130.00 129.93 129.50 129.36 128.91 128.71 128.68 128.65 128.62 128.56 128.53 128.45 128.00 127.16 126.90 122.71 105.77 105.33 103.89 102.59 87.24 86.66 86.40 85.81 85.22 85.02 83.97 83.85 83.16 82.54 76.14 75.64 73.62 72.67 71.74 70.41 68.65 68.63 62.79 62.66 31.01 30.98 22.47 22.25 21.72 21.38 18.78
PF6
Cl
Ru
N
OBz
BzO
BzO
OBz
O
Ru-2a (CDCl3, 100 MHz)
190 180 170 160 150 140 130 120 110 100 90 80 70 60 50 40 30 20 10
f1 (ppm)

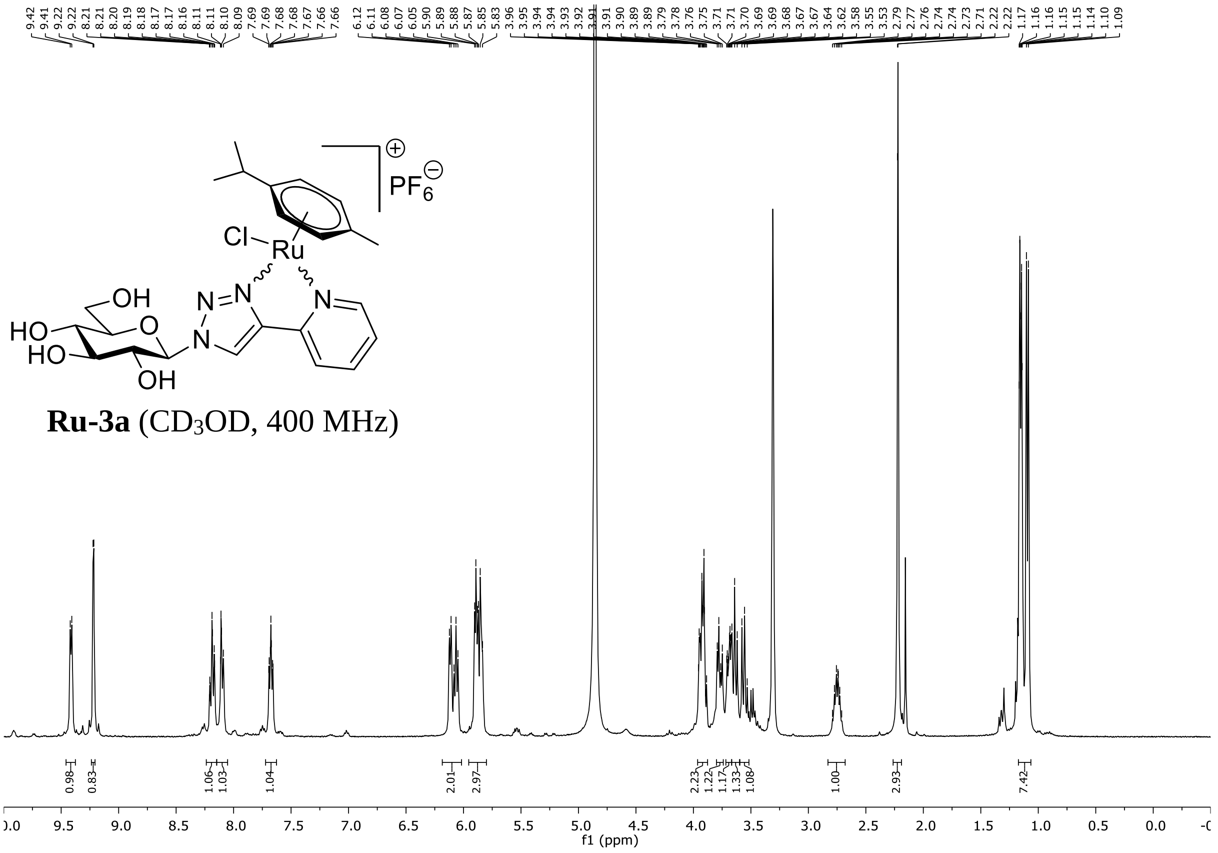
Ru-3a (CD3OD, 400 MHz)
f1 (ppm)

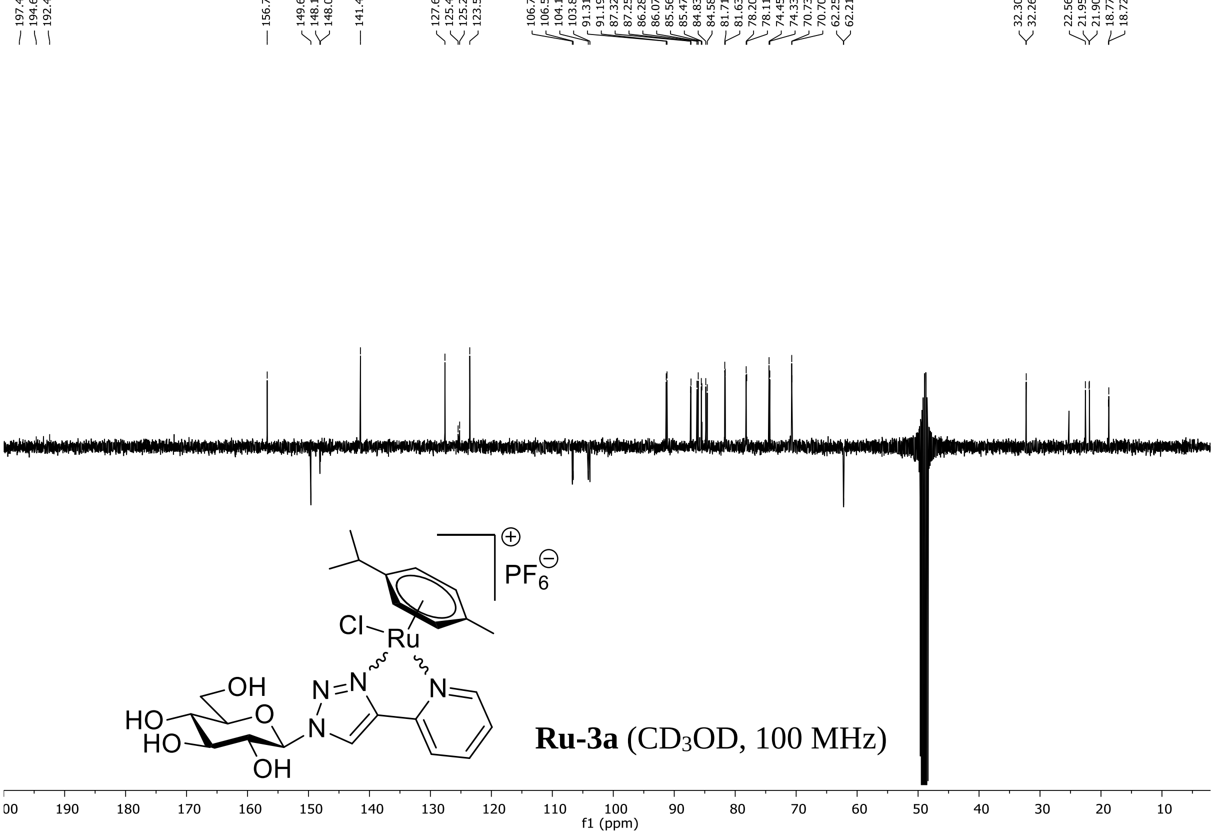
Ru-3a (CD3OD, 100 MHz)
f1 (ppm)

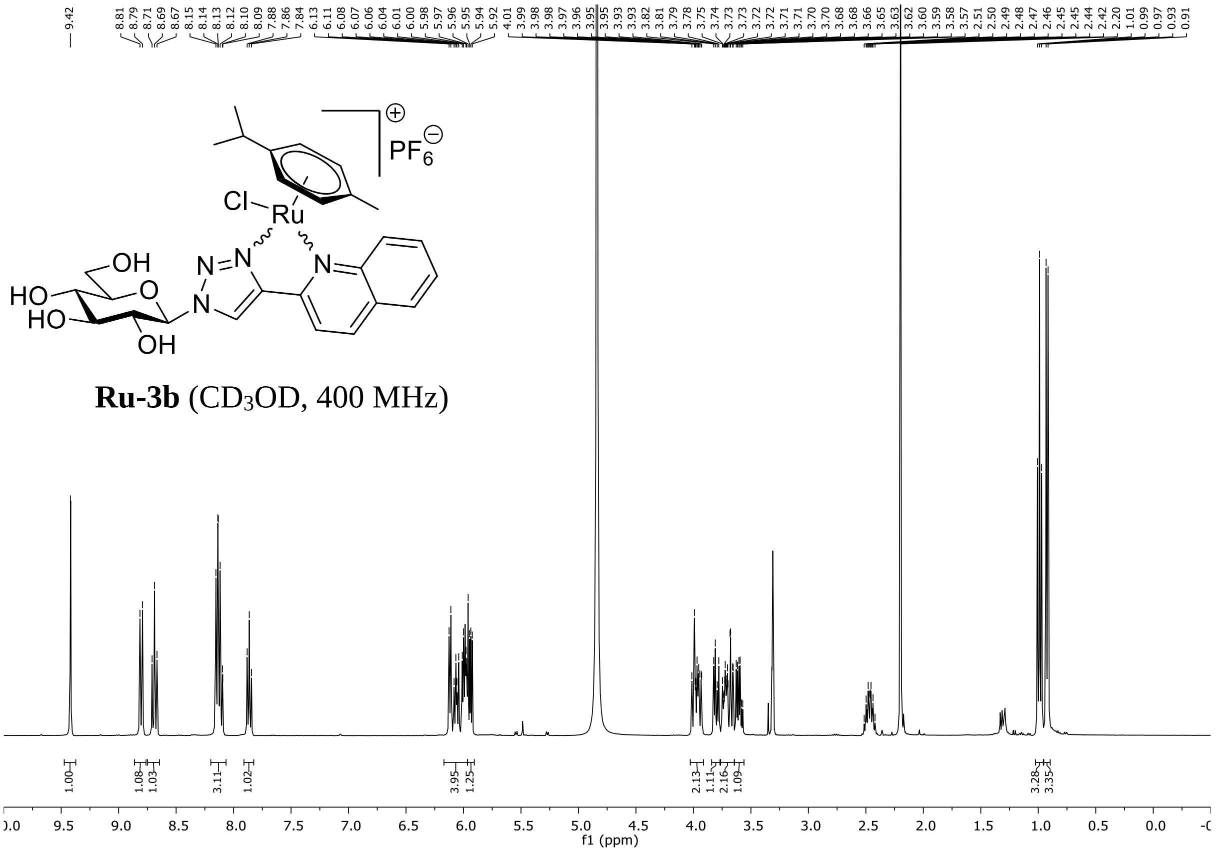

Ru-3b (CD_3OD, 400 MHz)

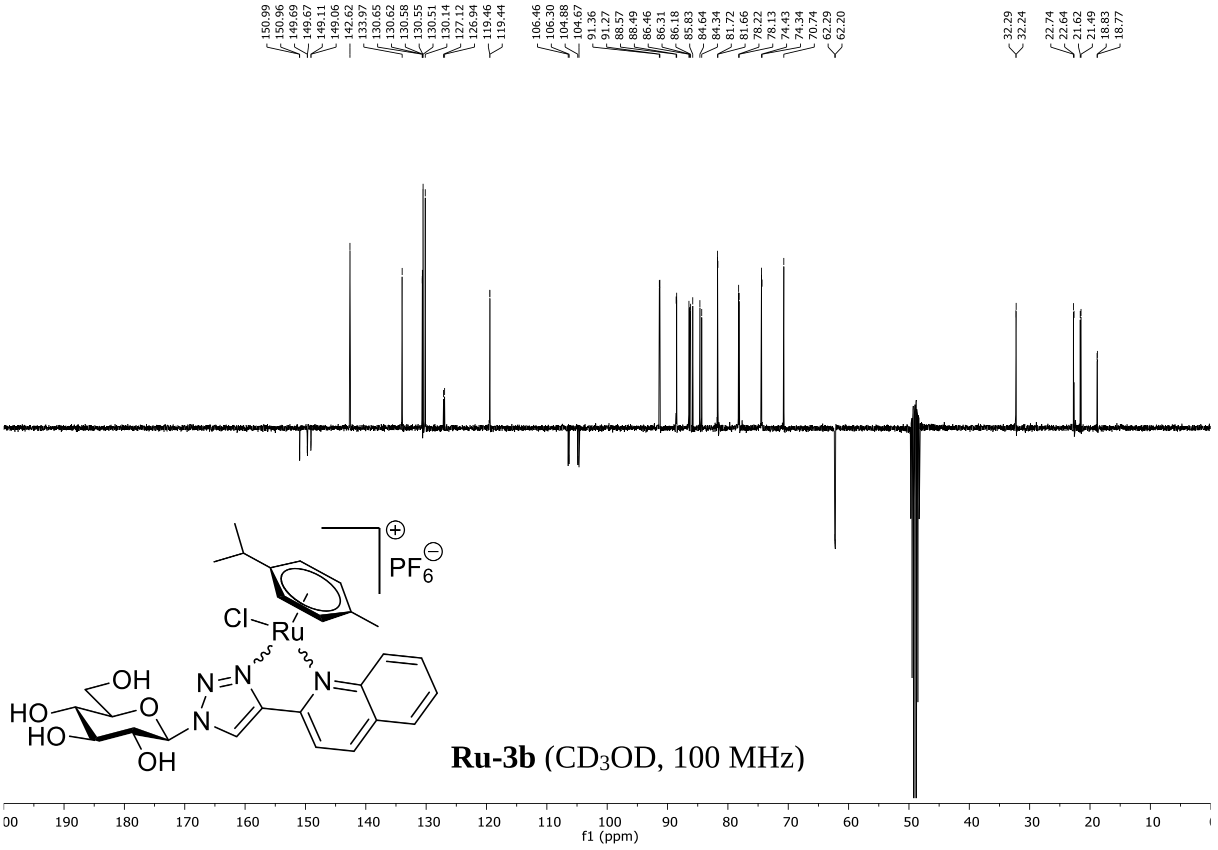

Ru-3b (CD_3OD, 100 MHz)

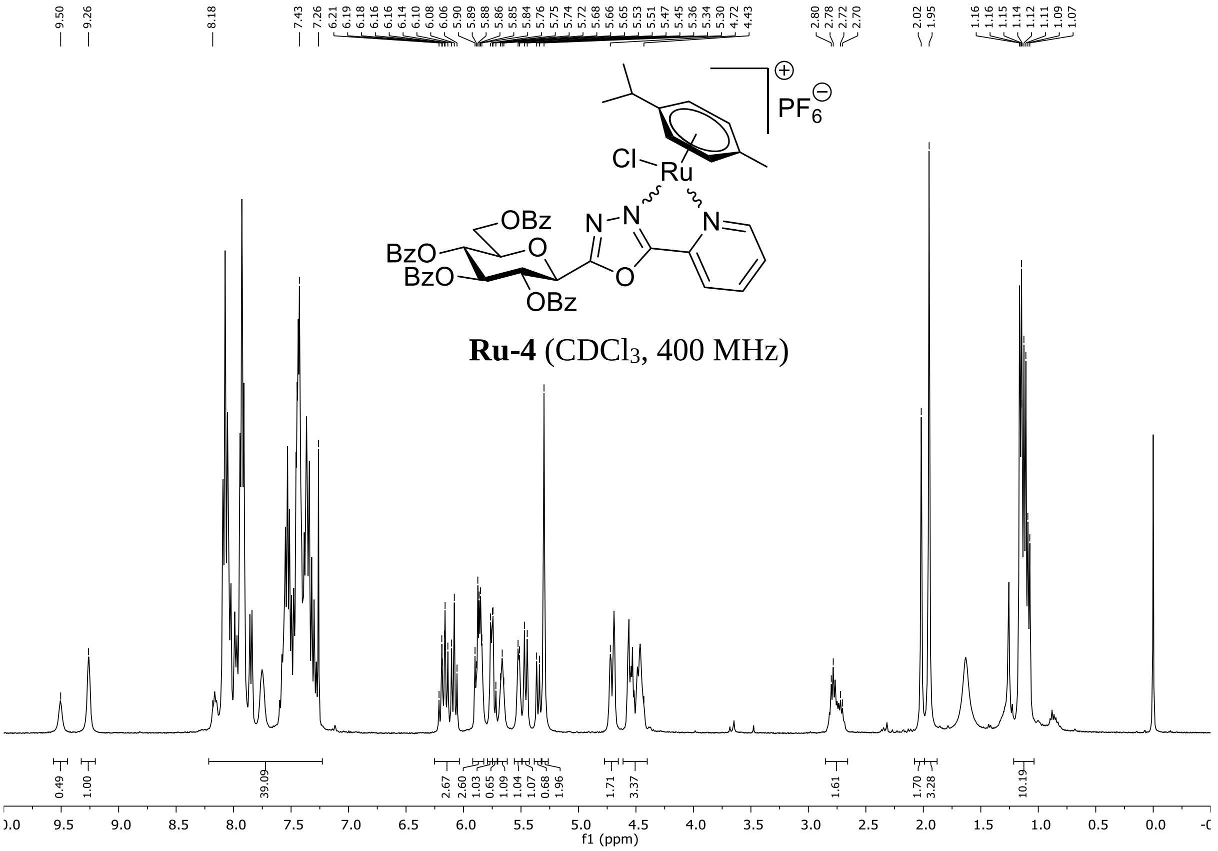
9.50
9.26
8.18
7.43
7.26
6.21 6.19 6.18 6.16 6.16 6.14 6.10 6.08 6.06 5.90 5.89 5.88 5.86 5.85 5.84 5.76 5.75 5.74 5.72 5.68 5.66 5.65 5.53 5.51 5.47 5.45 5.36 5.34 5.30 4.72 4.43
2.80 2.78 2.72 2.70
2.02 1.95
1.16 1.16 1.15 1.14 1.12 1.11 1.09 1.07
PF6
Cl
Ru
N
N
N
O
O
OBz
BzO
BzO
OBz
Ru-4 (CDCl3, 400 MHz)
0.49 1.00 39.09 2.67 2.60 1.03 0.65 1.09 1.04 1.07 0.68 1.96 1.71 3.37 1.61 1.70 3.28 10.19
9.5 9.0 8.5 8.0 7.5 7.0 6.5 6.0 5.5 5.0 4.5 4.0 3.5 3.0 2.5 2.0 1.5 1.0 0.5 0.0
f1 (ppm)

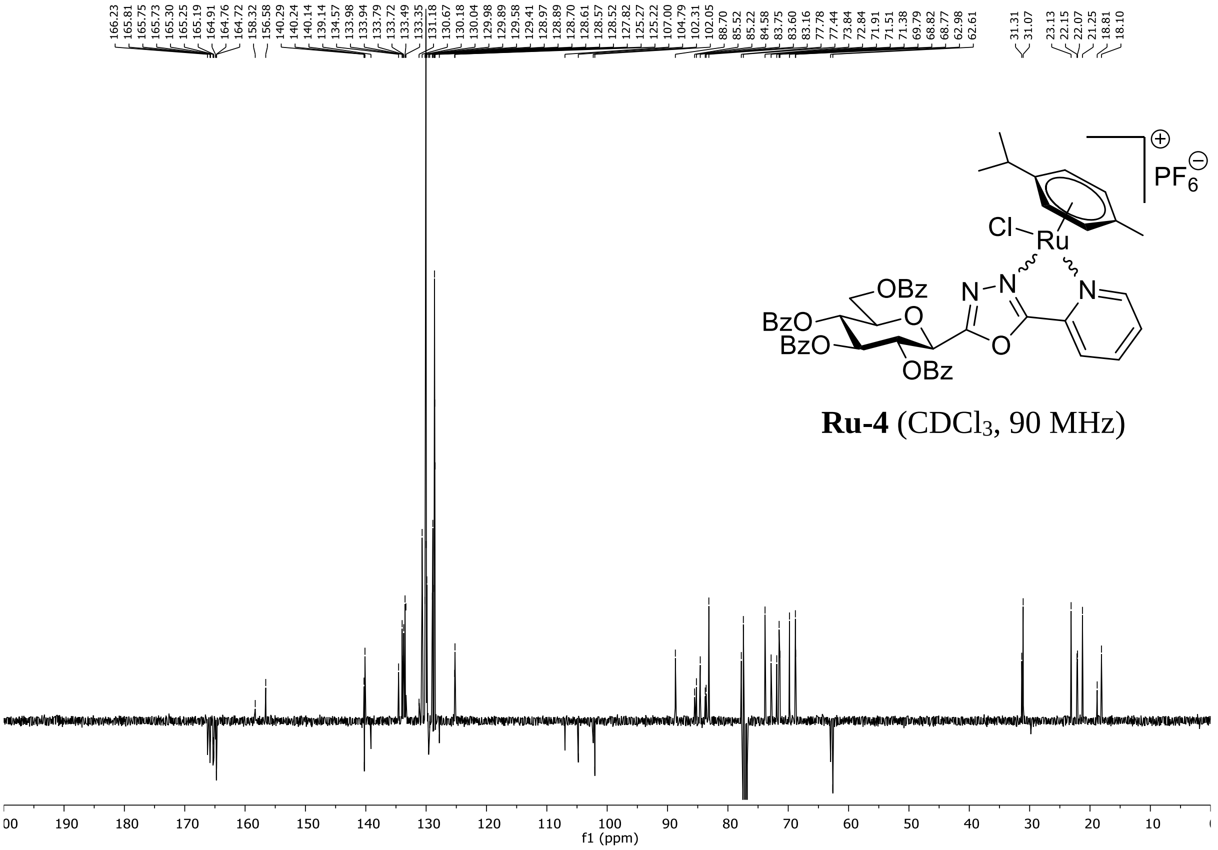
166.23 165.81 165.75 165.73 165.30 165.25 165.19 164.91 164.76 164.72 158.32 156.58 140.29 140.24 140.14 139.14 134.57 133.98 133.94 133.79 133.72 133.49 133.35 131.18 130.67 130.18 130.04 129.98 129.89 129.58 129.41 128.97 128.89 128.70 128.61 128.57 128.52 127.82 125.27 125.22 107.00 104.79 102.31 102.05 88.70 85.52 85.22 84.58 83.75 83.60 83.16 77.78 77.44 73.84 72.84 71.91 71.51 71.38 69.79 68.82 68.77 62.98 62.61
31.31 31.07
23.13 22.15 22.07 21.25 18.81 18.10
PF6
Cl
Ru
N
N
N
O
O
OBz
BzO
BzO
OBz
Ru-4 (CDCl3, 90 MHz)
190 180 170 160 150 140 130 120 110 100 90 80 70 60 50 40 30 20 10
f1 (ppm)

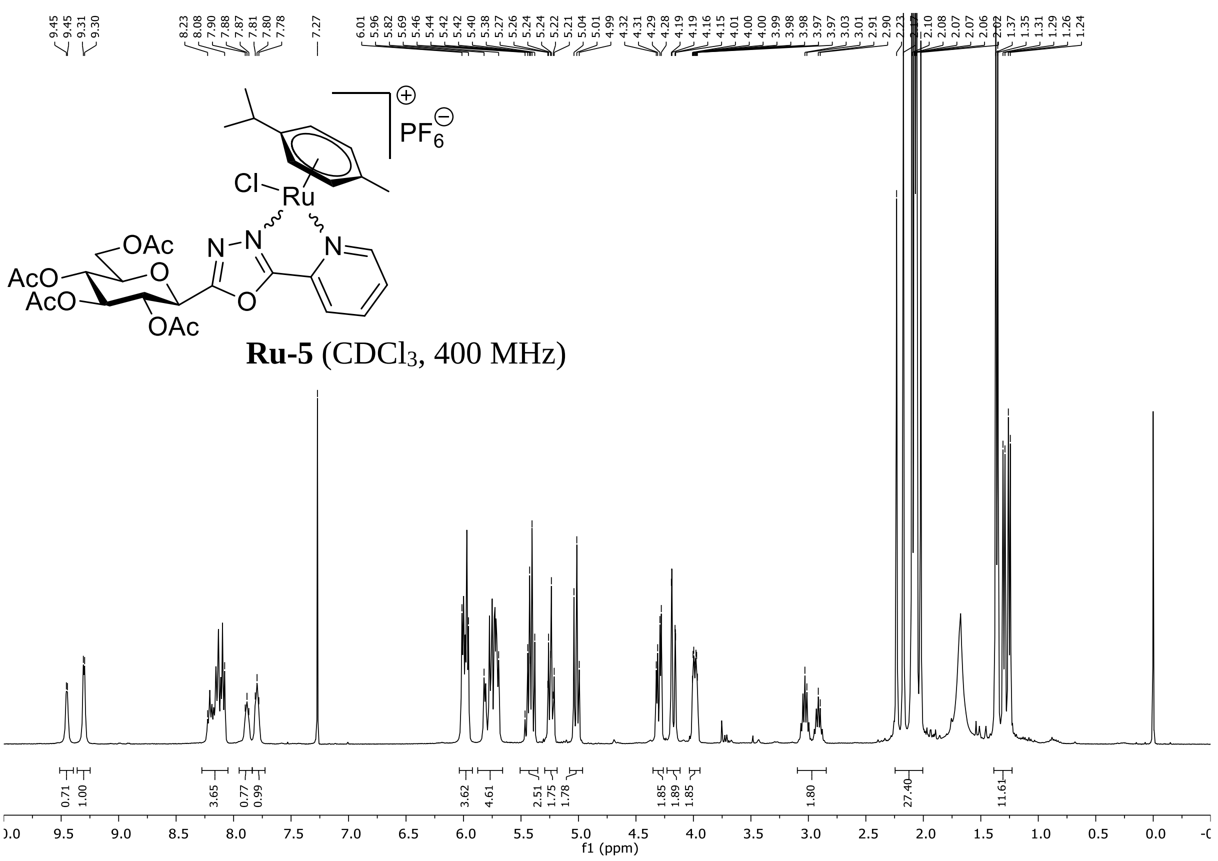

Ru-5 ($CDCl_3$, 400 MHz)

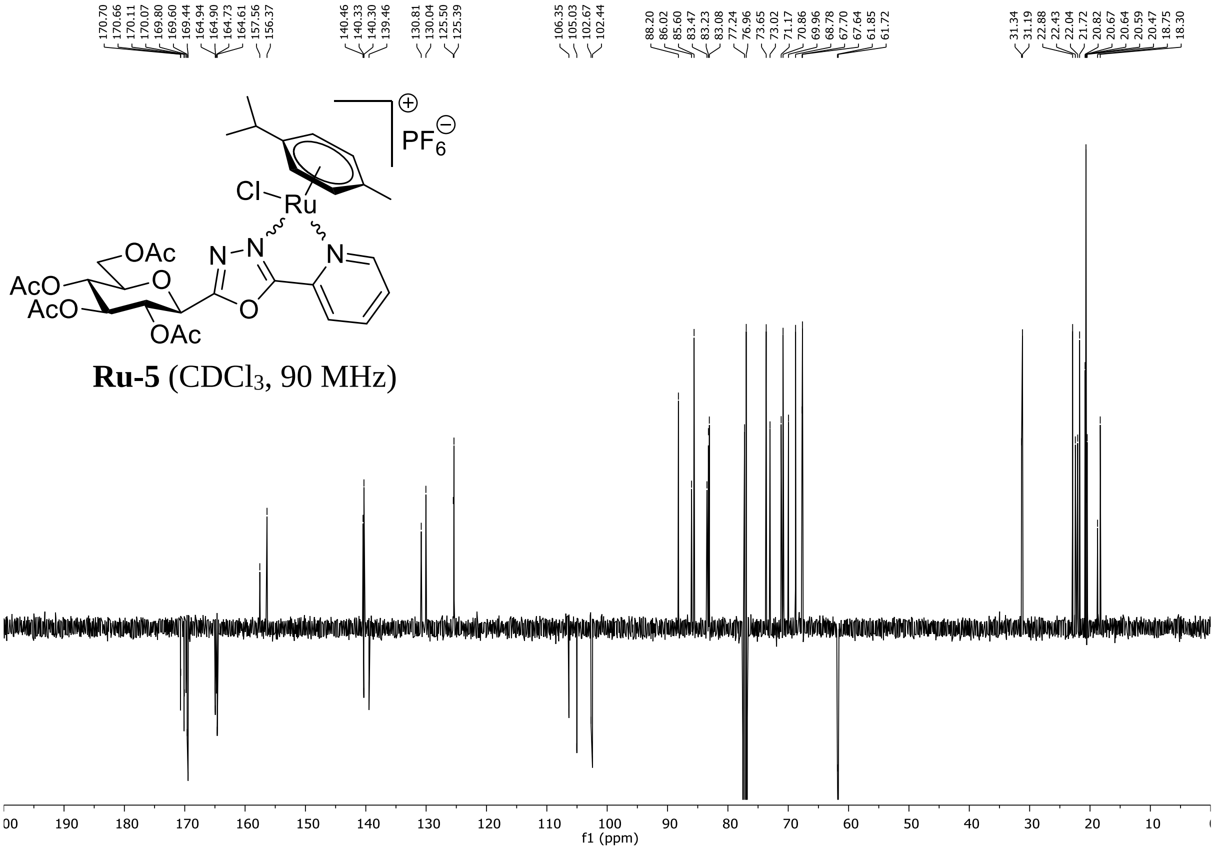

Ru-5 ($CDCl_3$, 90 MHz)

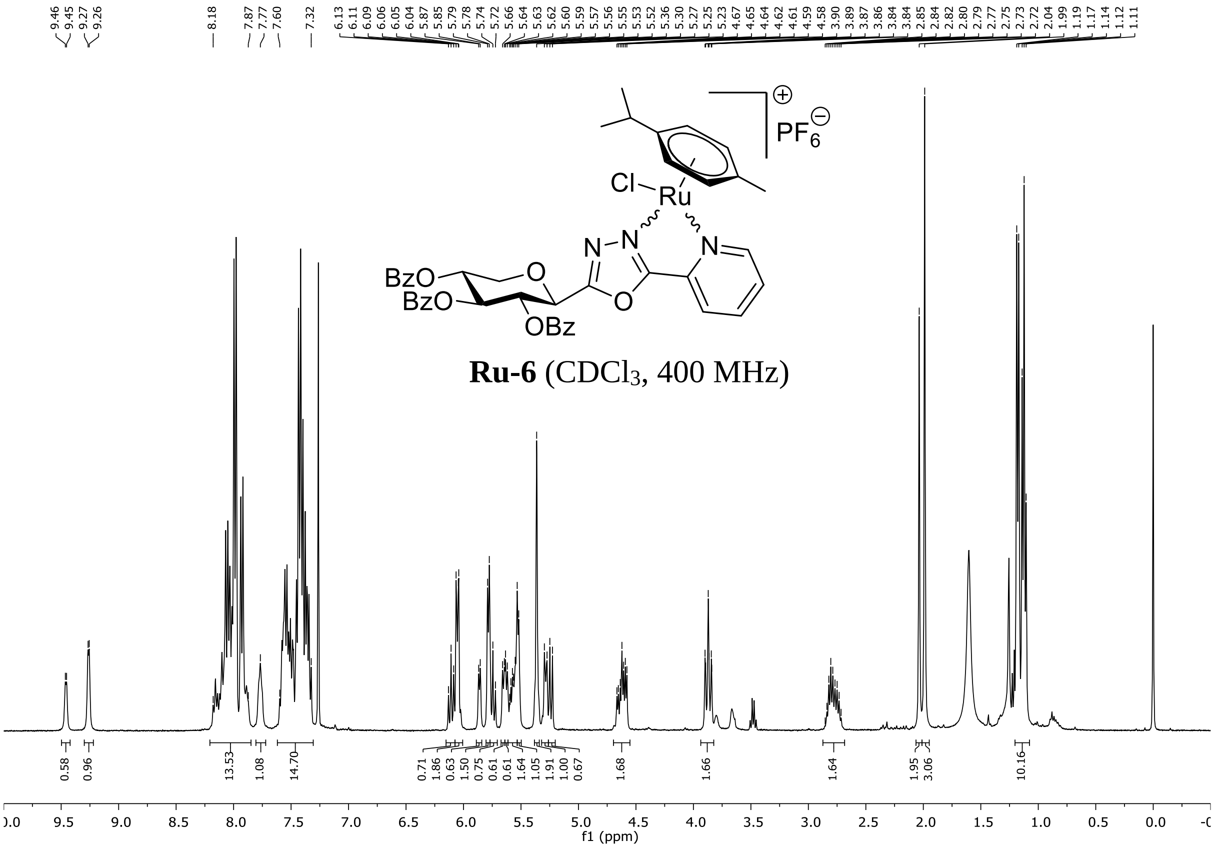
PF6
Cl
Ru
N
N
N
O
BzO
BzO
O
OBz
Ru-6 (CDCl3, 400 MHz)
f1 (ppm)

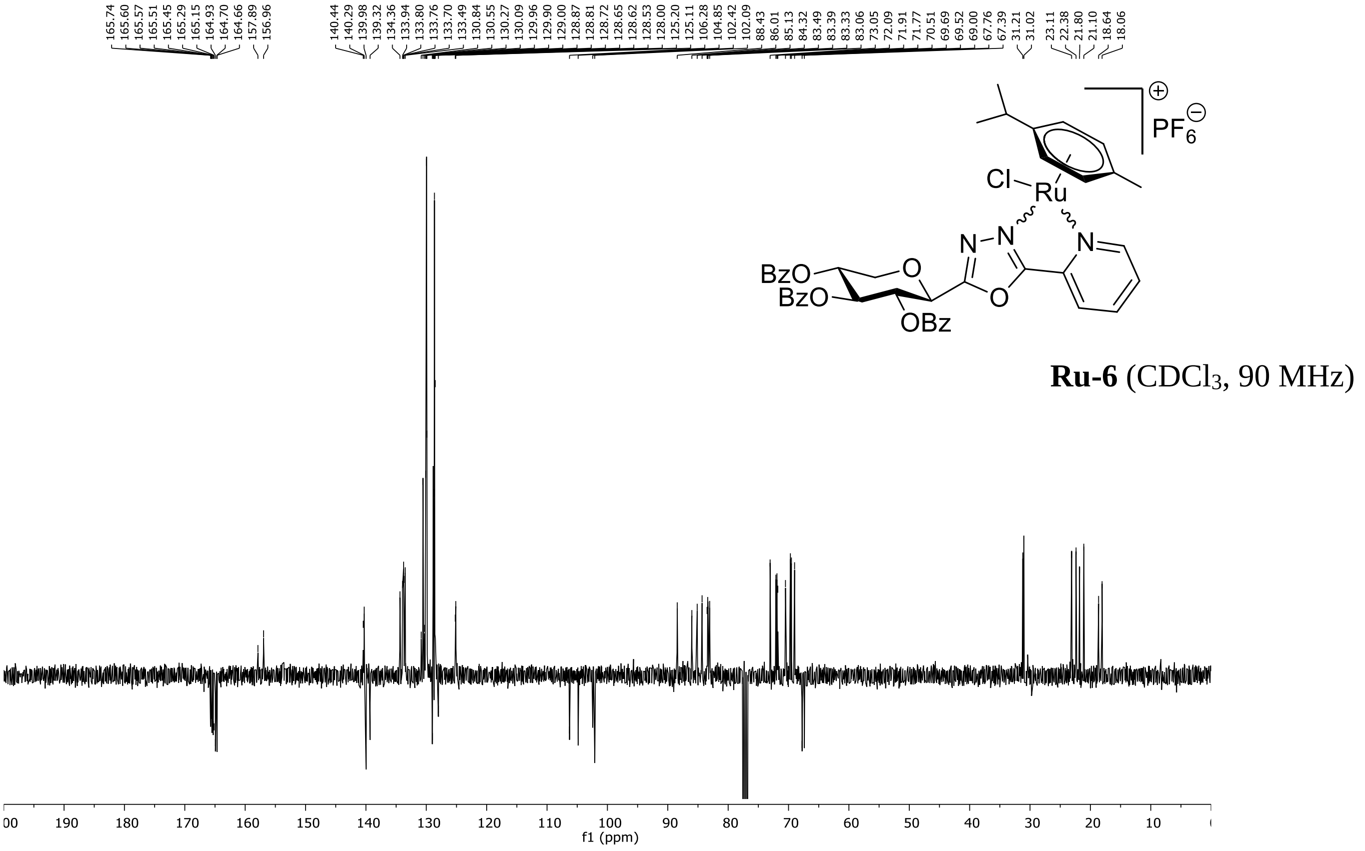
PF6
Cl
Ru
N
N
N
O
BzO
BzO
O
OBz
Ru-6 (CDCl3, 90 MHz)
f1 (ppm)

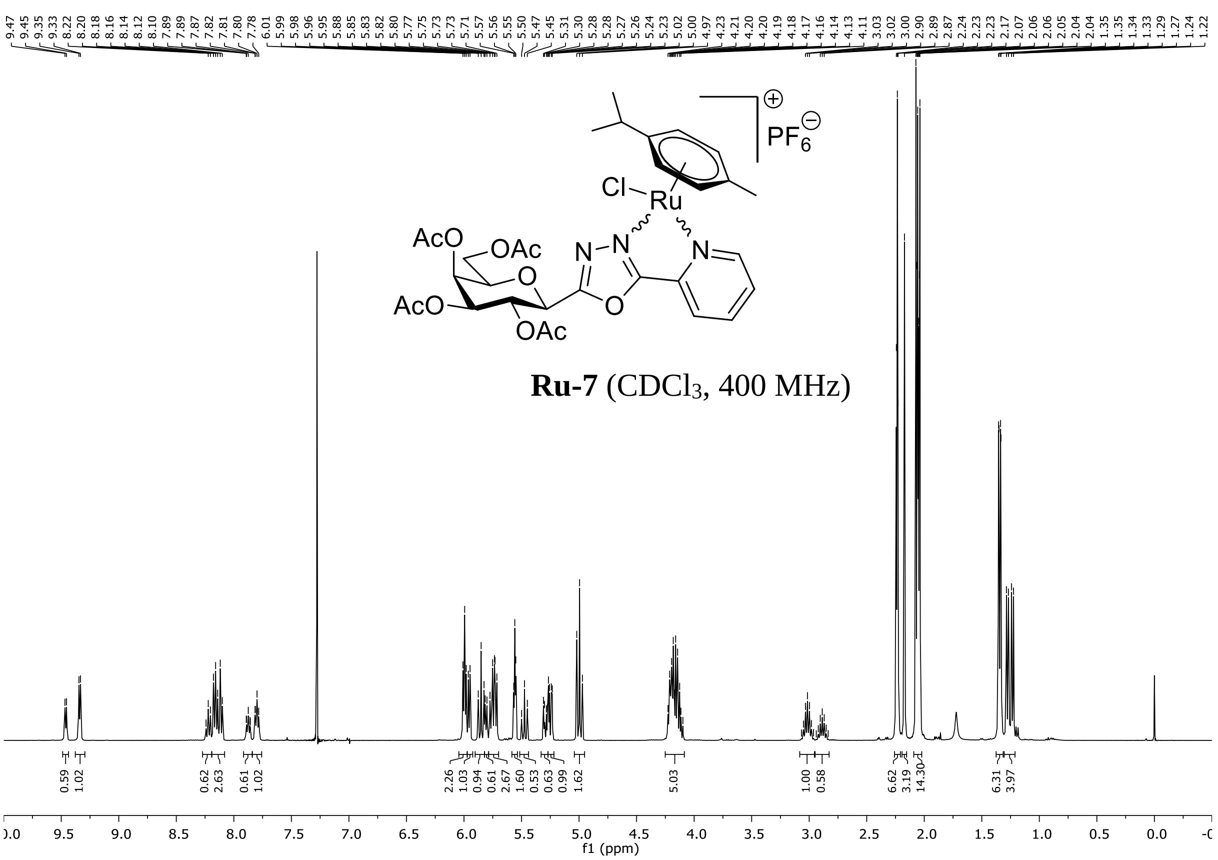
Ru-7 (CDCl3, 400 MHz)
f1 (ppm)

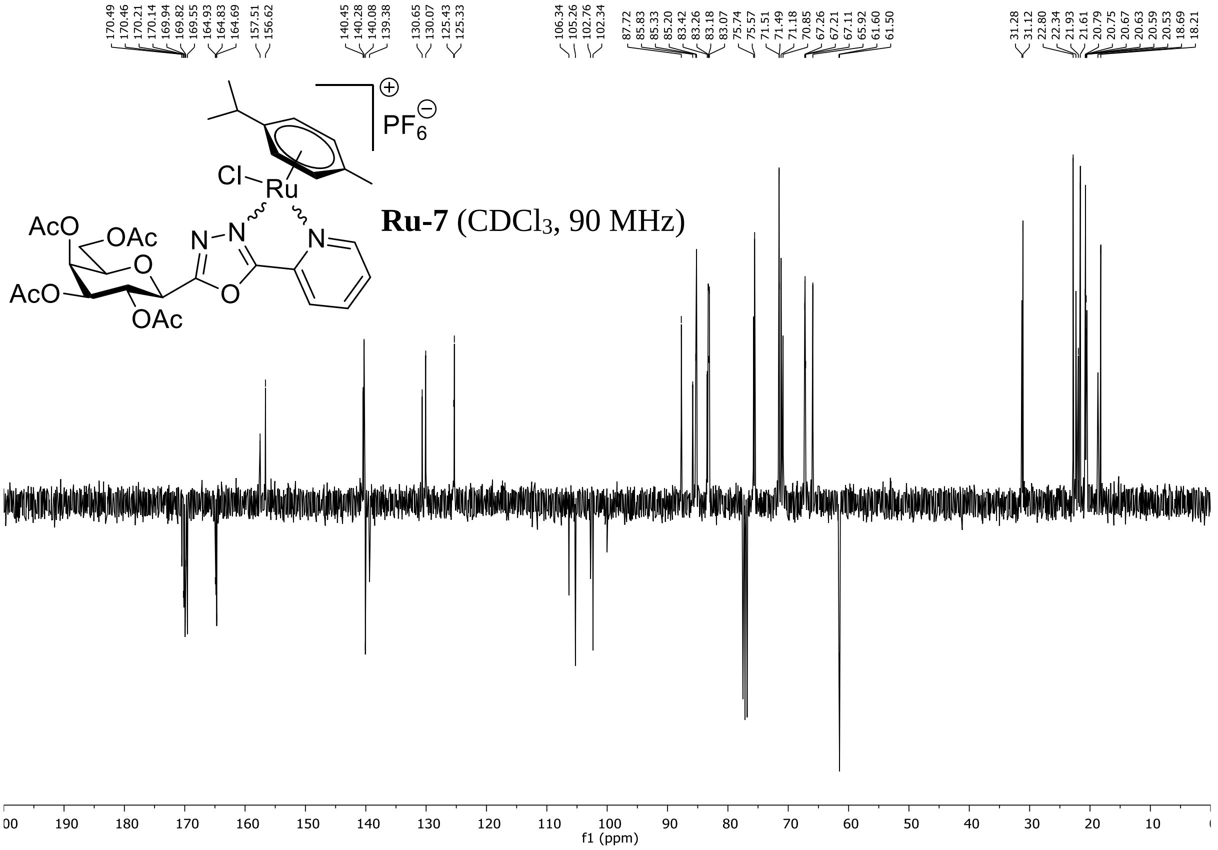
Ru-7 (CDCl3, 90 MHz)
f1 (ppm)

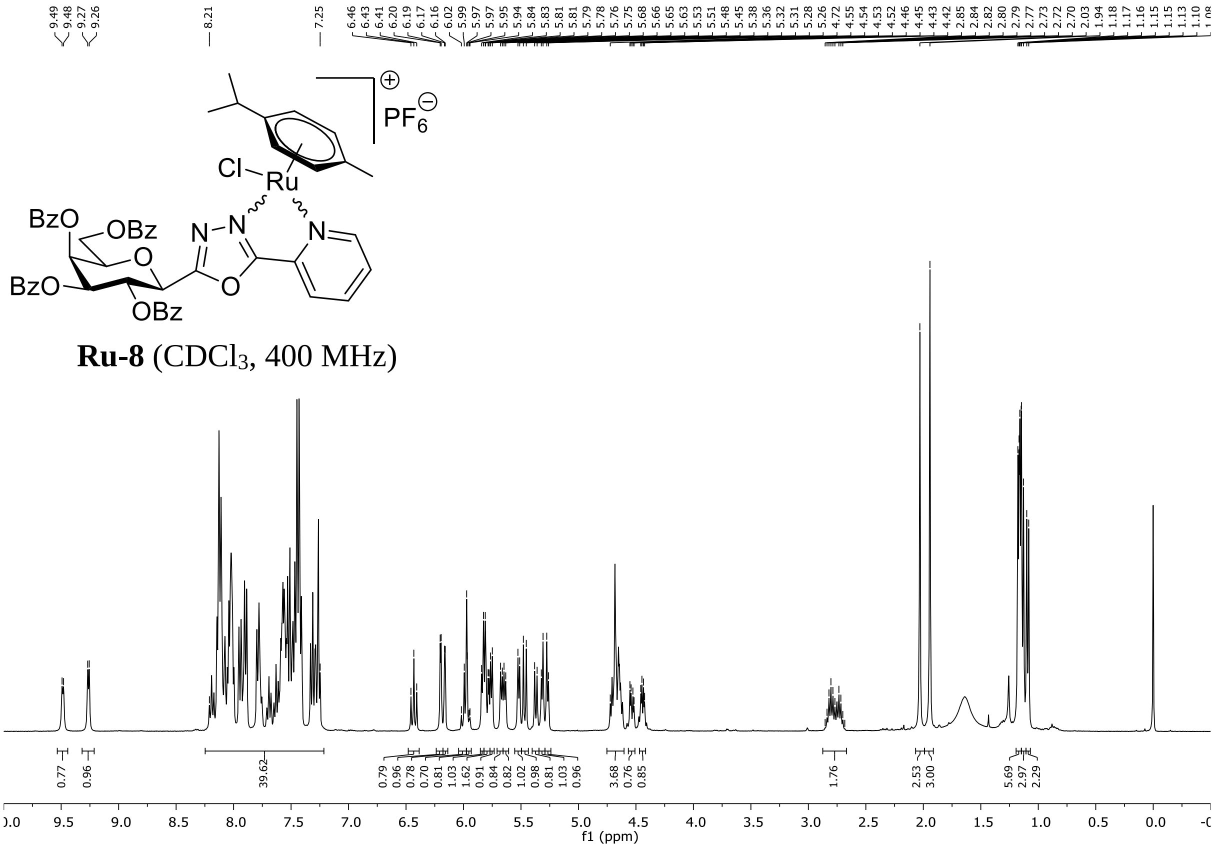

Ru-8 (CDCl$_3$, 400 MHz)

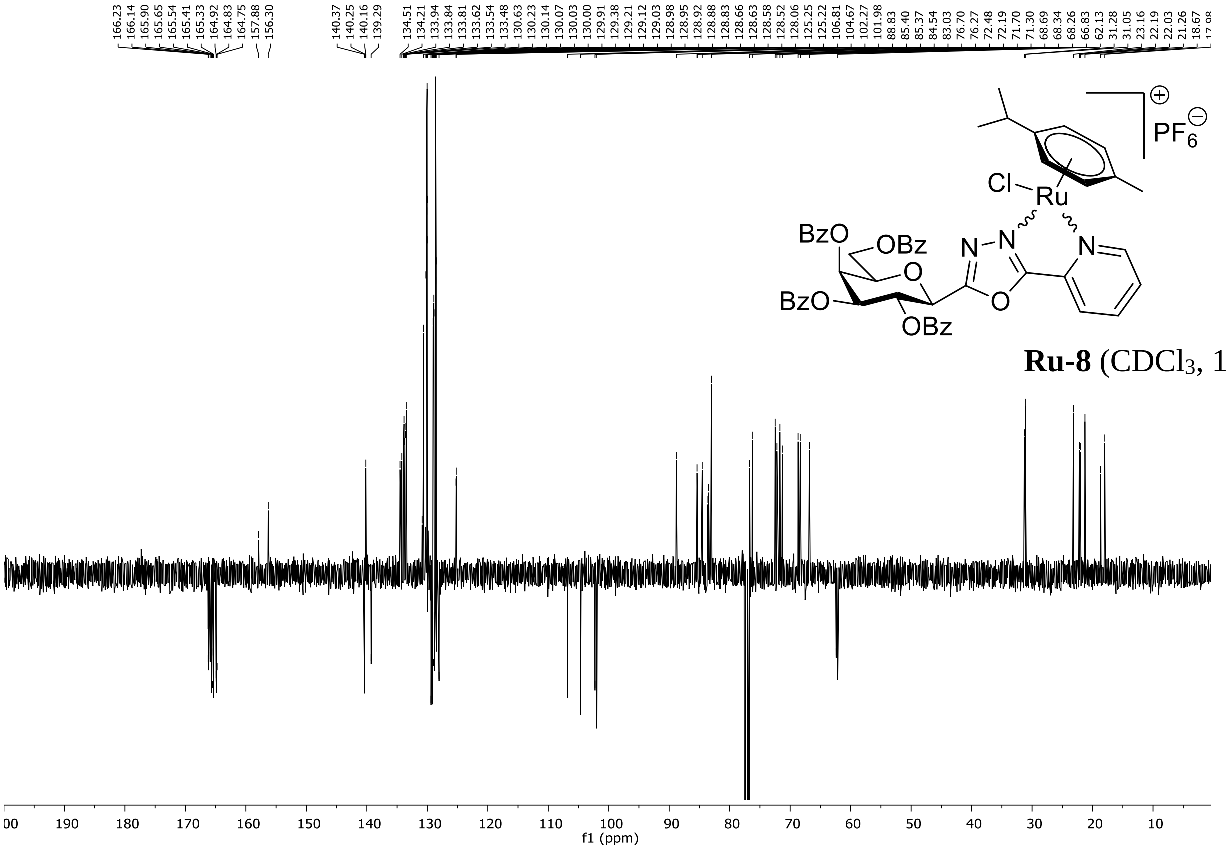

Ru-8 (CDCl$_3$, 100 MHz)

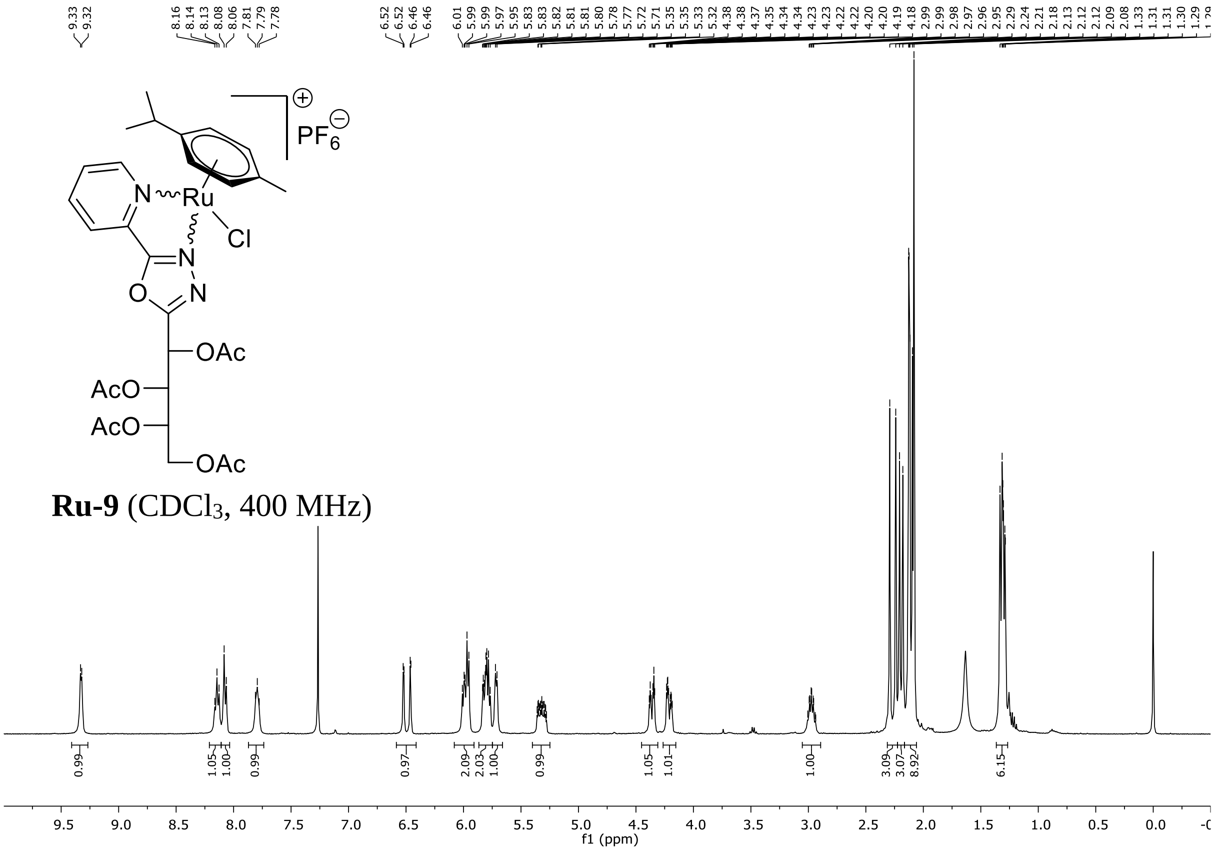

Ru-9 (CDCl$_3$, 400 MHz)

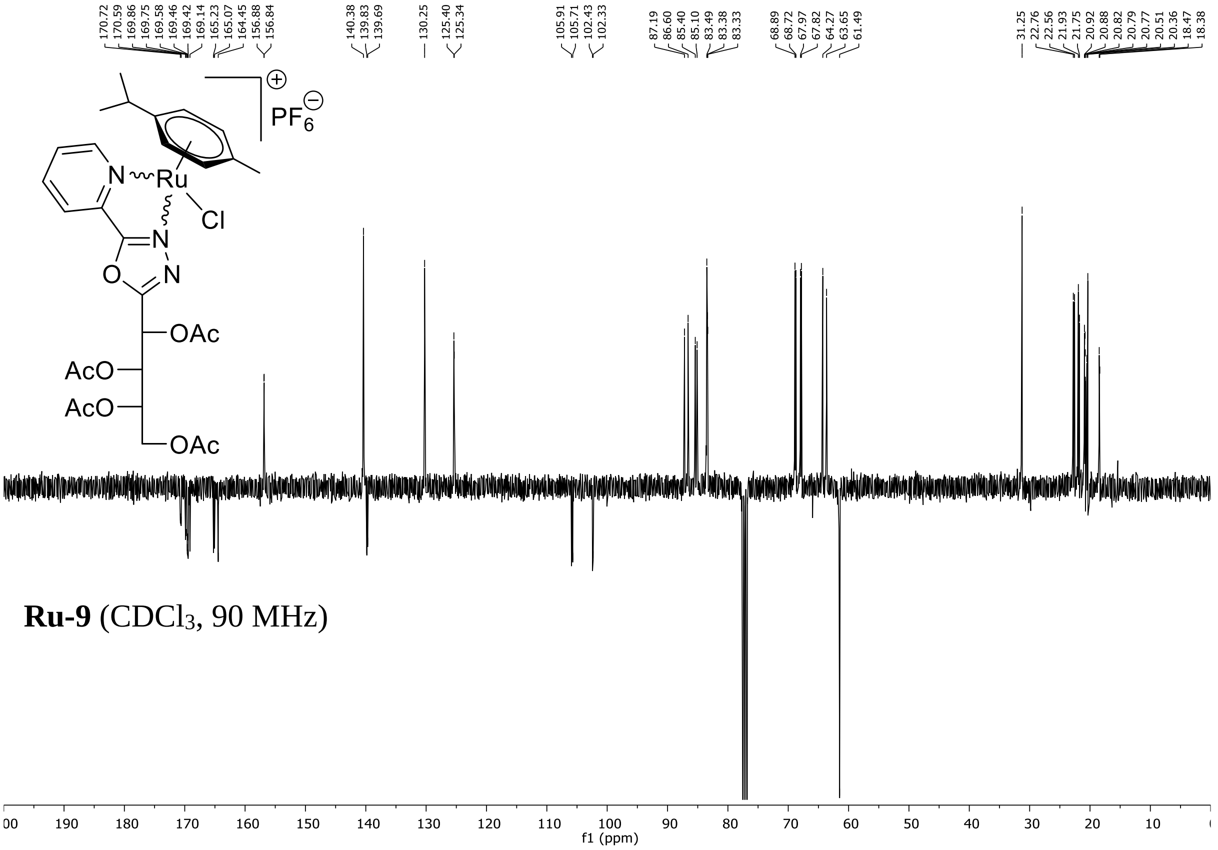

Ru-9 (CDCl$_3$, 90 MHz)

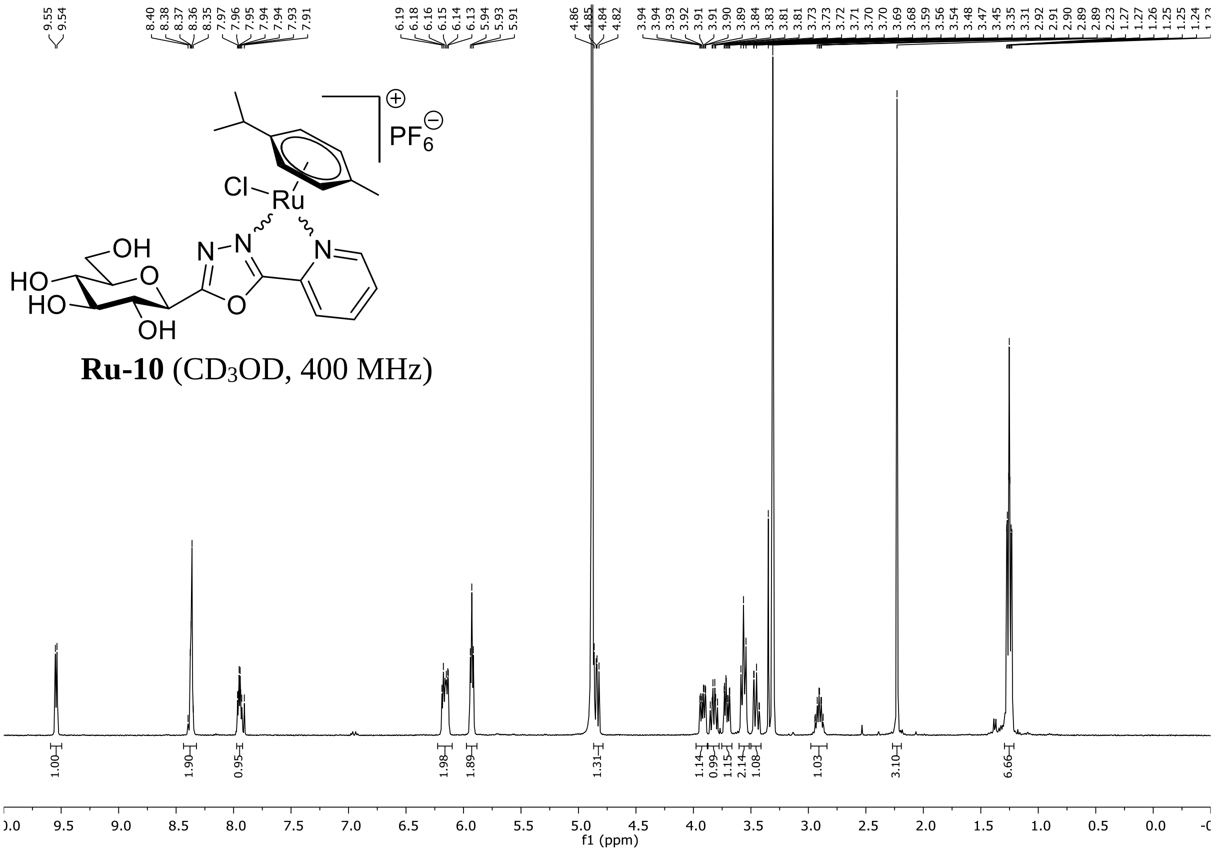

Ru-10 (CD$_3$OD, 400 MHz)

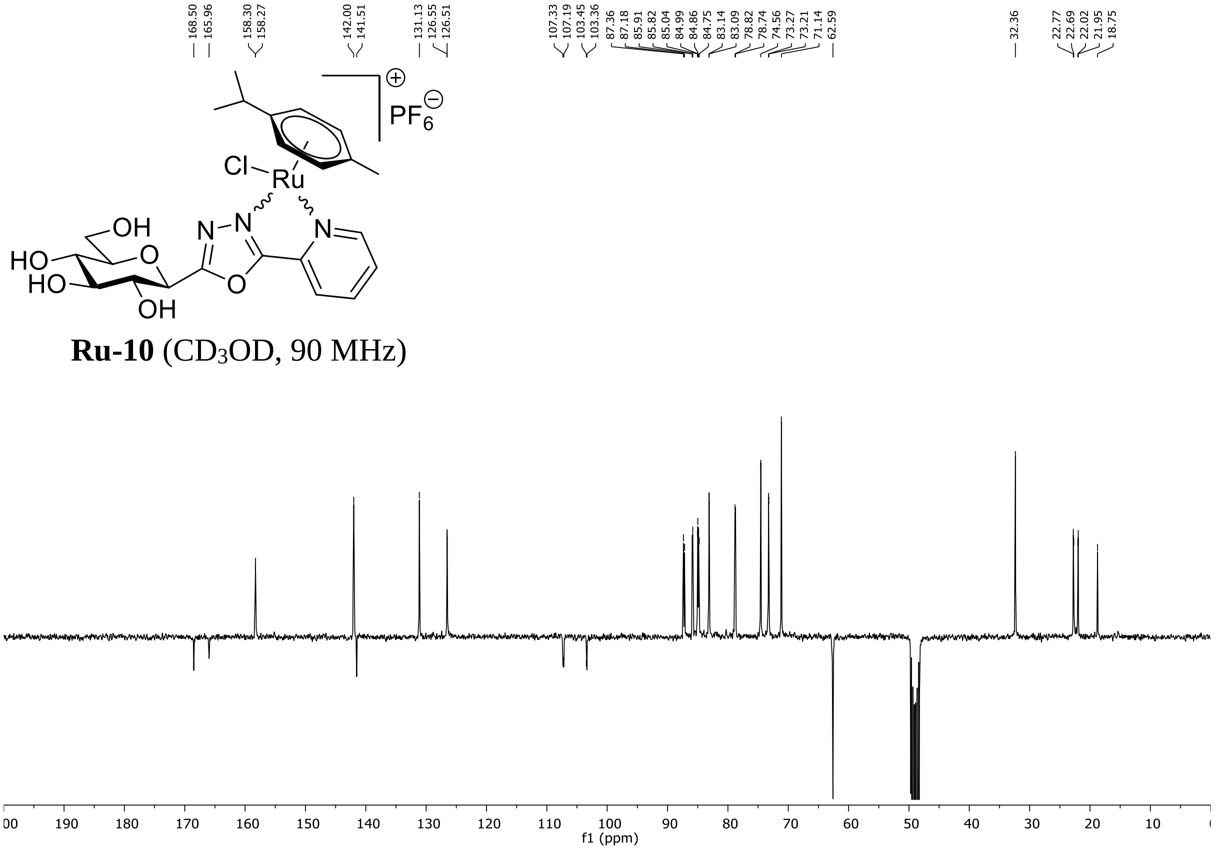

Ru-10 (CD$_3$OD, 90 MHz)

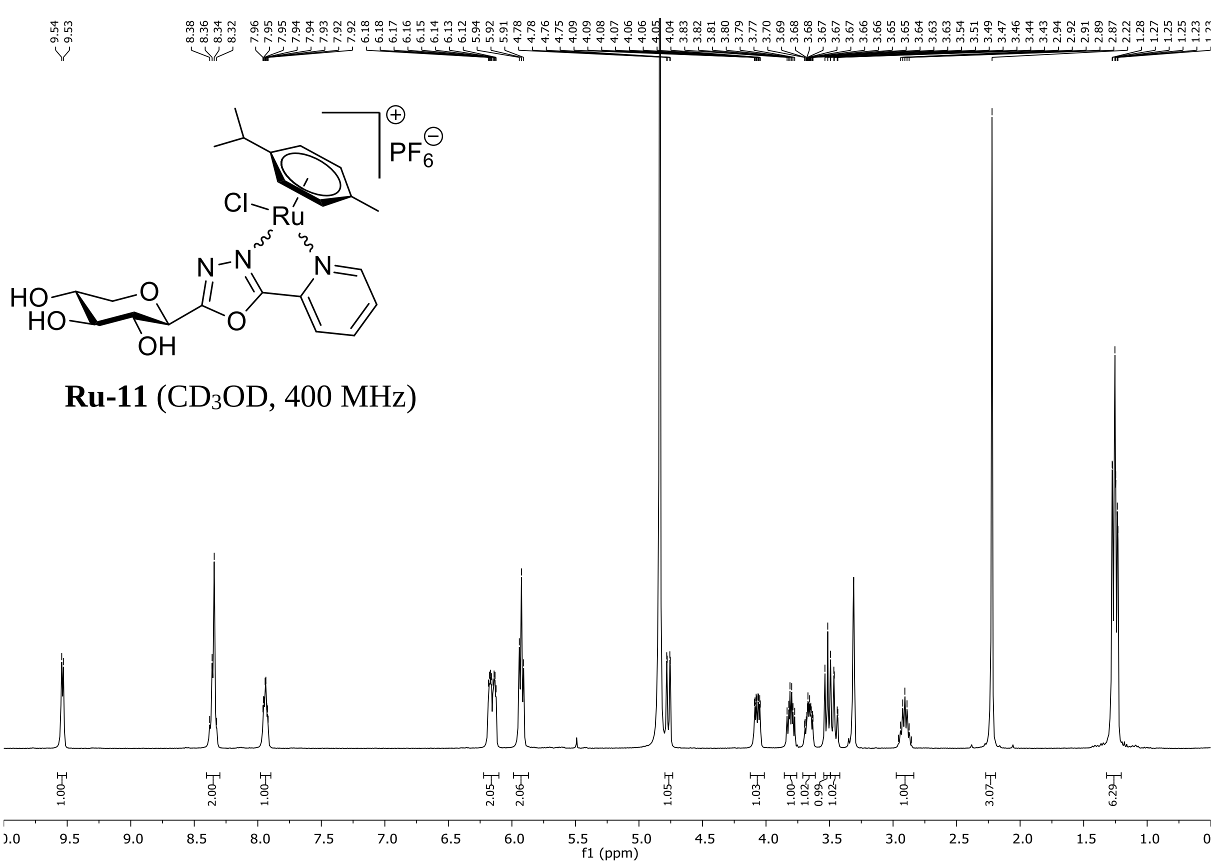
Cl
Ru
PF6
N
O
HO
HO
OH
Ru-11 (CD3OD, 400 MHz)
f1 (ppm)

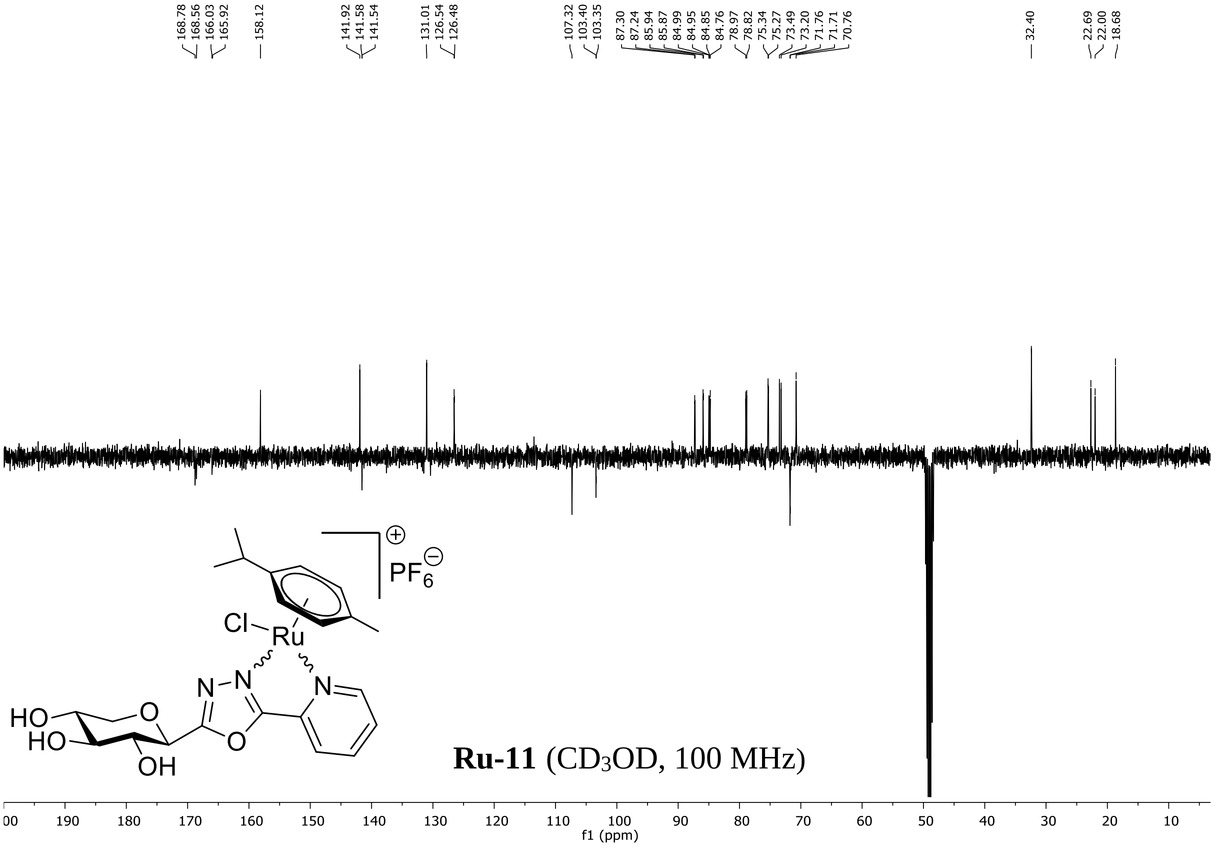
Cl
Ru
PF6
N
O
HO
HO
OH
Ru-11 (CD3OD, 100 MHz)
f1 (ppm)

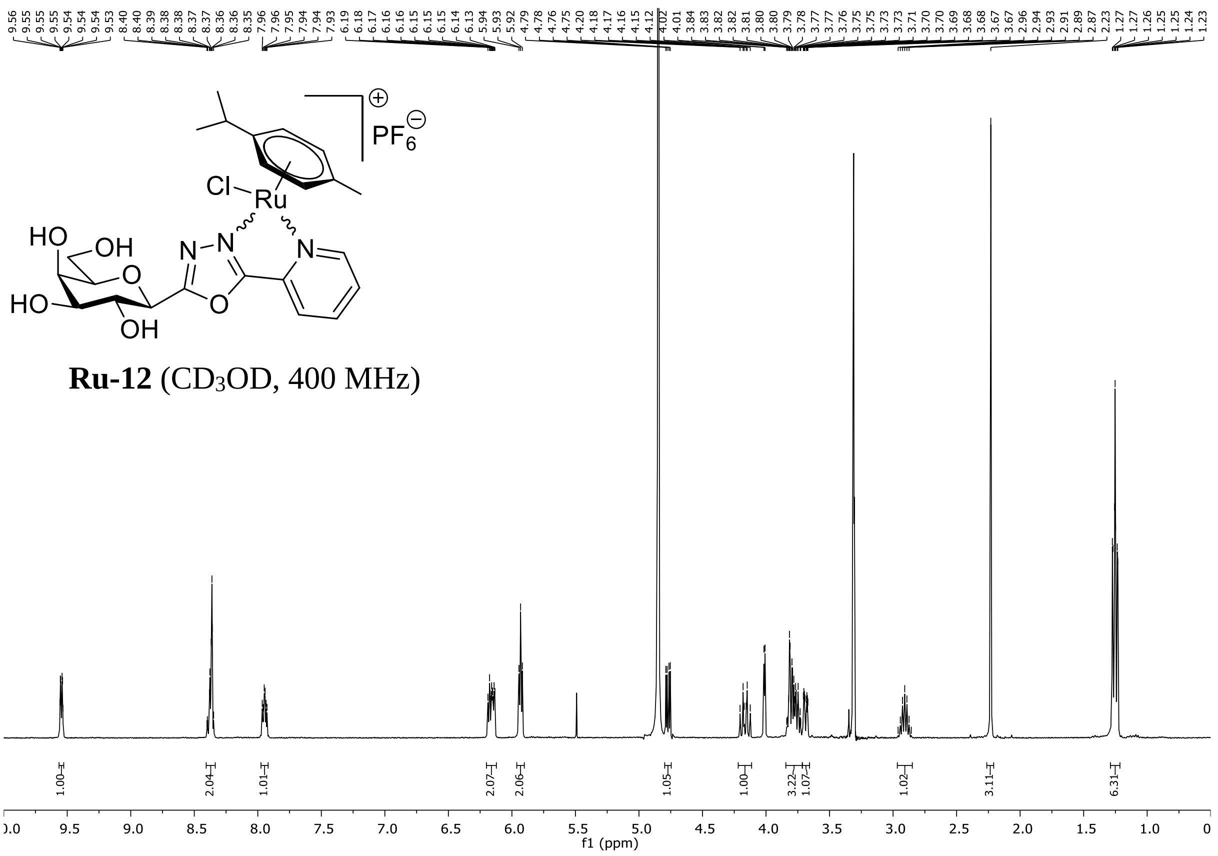

Ru-12 (CD3OD, 400 MHz)

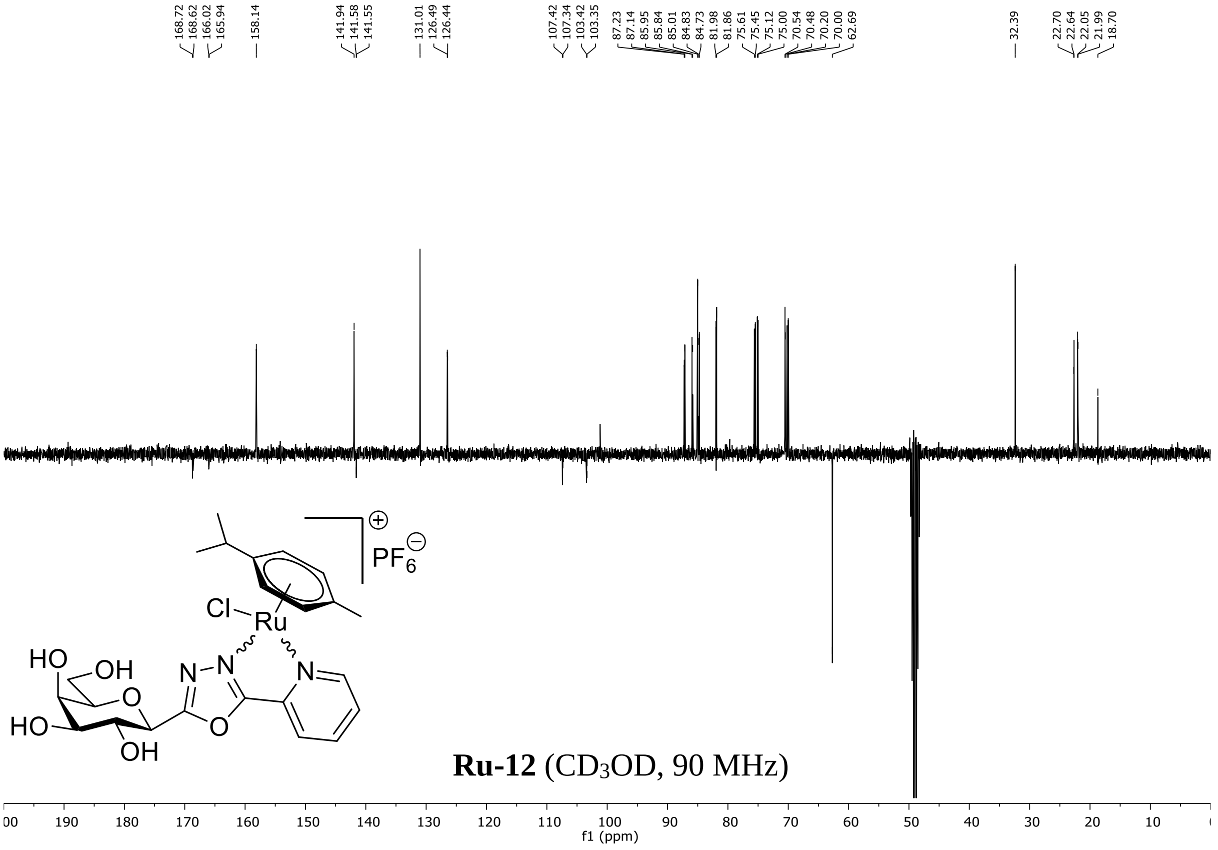

Ru-12 (CD3OD, 90 MHz)

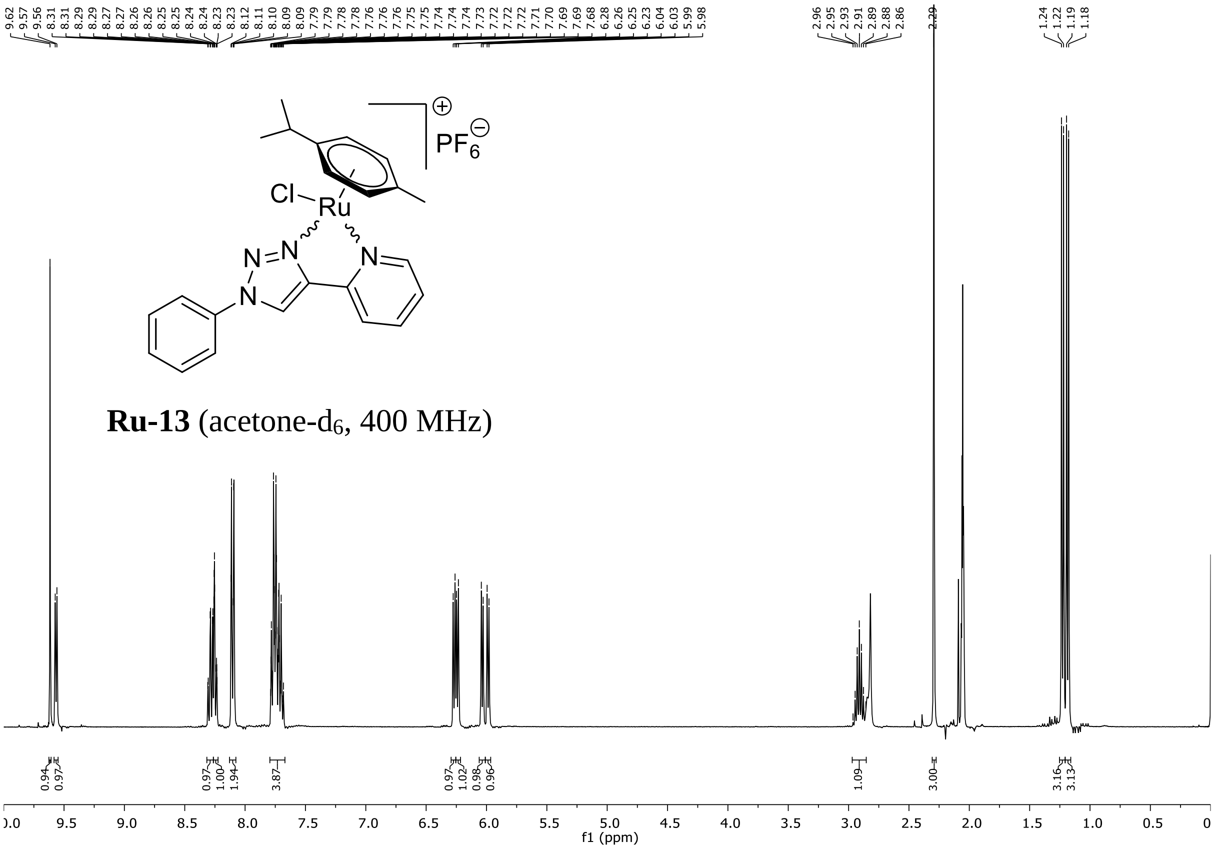
9.62 9.57 9.56 8.31 8.31 8.29 8.29 8.27 8.27 8.26 8.26 8.25 8.25 8.24 8.24 8.23 8.23 8.12 8.11 8.10 8.09 8.09 7.79 7.79 7.78 7.78 7.76 7.76 7.76 7.75 7.75 7.74 7.74 7.74 7.73 7.72 7.72 7.72 7.71 7.70 7.69 7.69 7.68 6.28 6.26 6.25 6.23 6.04 6.03 5.99 5.98
2.96 2.95 2.93 2.91 2.89 2.88 2.86
2.29
1.24 1.22 1.19 1.18
Cl
Ru
N
N
N
N
PF6
Ru-13 (acetone-d6, 400 MHz)
0.94 0.97 0.97 1.00 1.94 3.87 0.97 1.02 0.98 0.96 1.09 3.00 3.16 3.13
f1 (ppm)

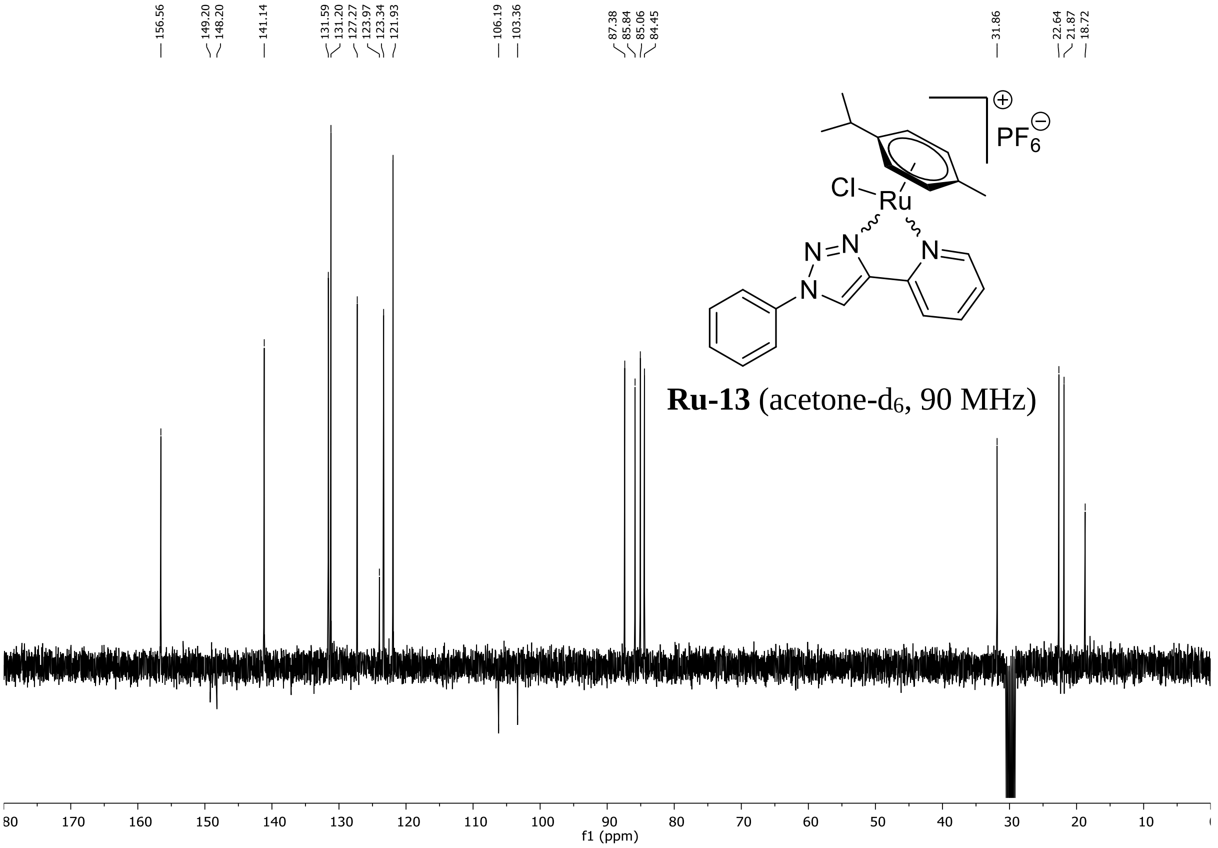
156.56
149.20 148.20
141.14
131.59 131.20 127.27 123.97 123.34 121.93
106.19 103.36
87.38 85.84 85.06 84.45
31.86
22.64 21.87 18.72
Cl
Ru
N
N
N
N
PF6
Ru-13 (acetone-d6, 90 MHz)
f1 (ppm)

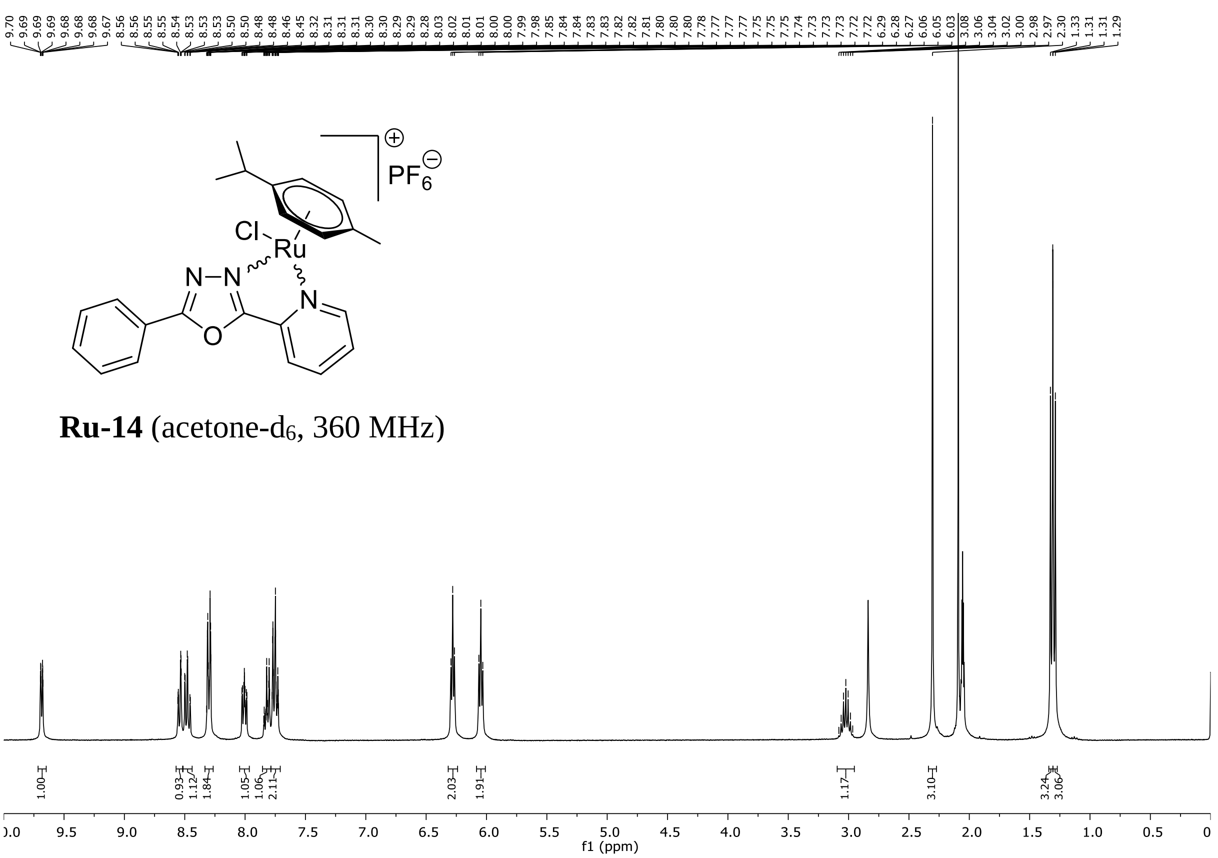

Ru-14 (acetone-d$_6$, 360 MHz)

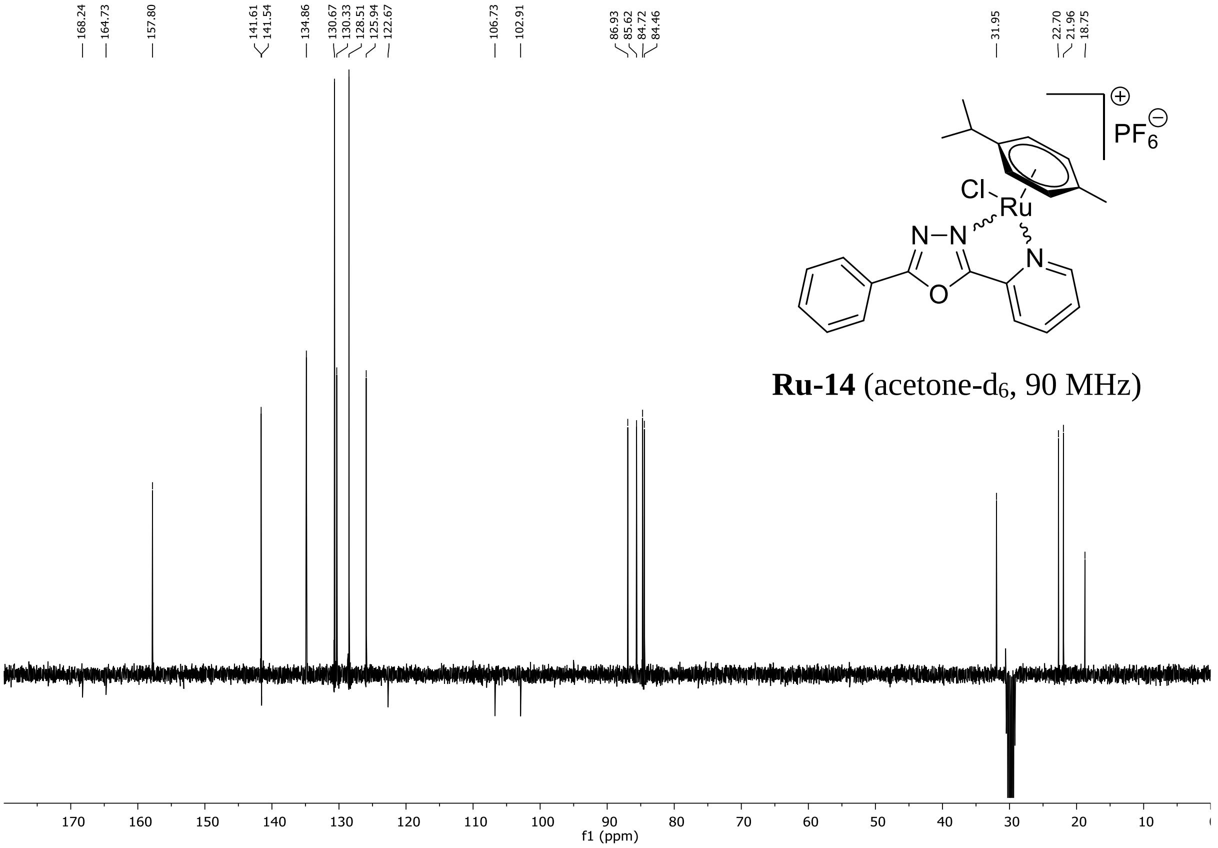

Ru-14 (acetone-d$_6$, 90 MHz)

3. **A representative example for the stability of the complexes in aqueous medium**

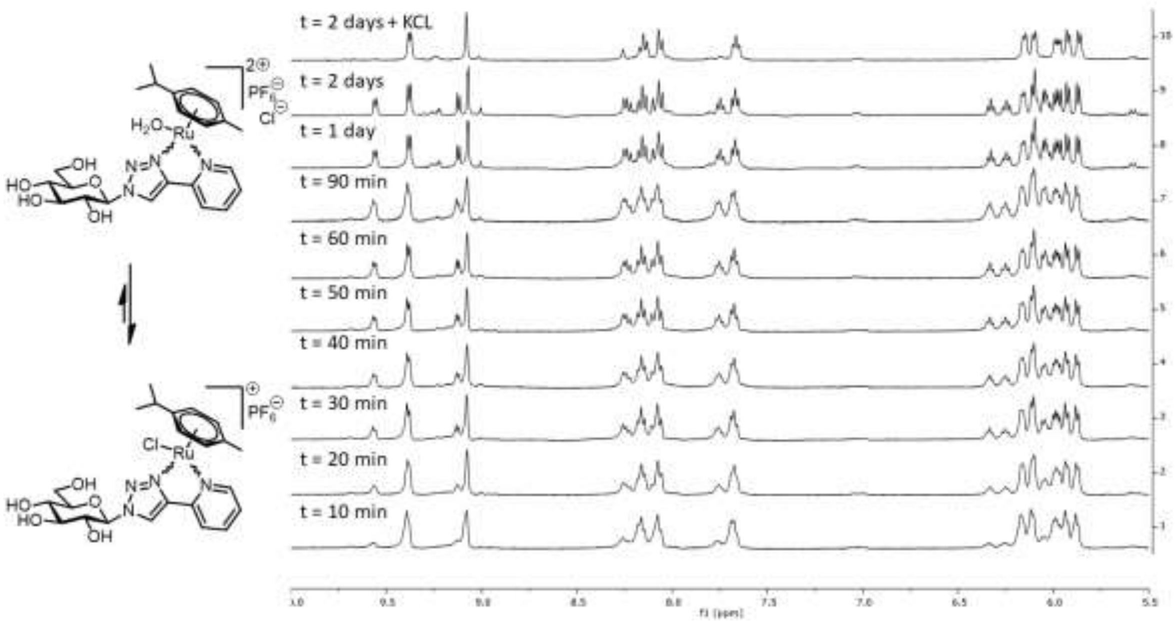

Figure S1. Time dependence of the ^{1}H NMR spectra of **Ru-3a** in D_2O over a 2 day period. By the addition of KCl (100 eq.) the original spectrum could be recovered indicating only Cl^-/D_2O exchange during the period of time of the study.

4. **Table S9.** Distribution coefficient of the synthesized complexes (logD)

Complex		LogD	Complex		logD
**Ru-1a**	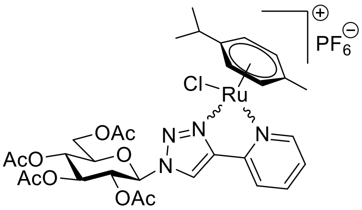	-1.09	**Ru-7**	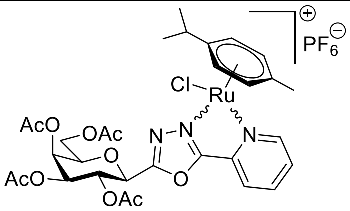	-1.29
**Ru-1b**	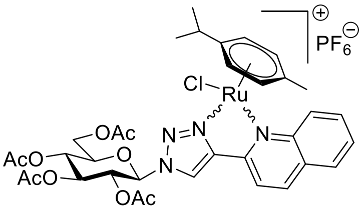	-0.97	**Ru-8**	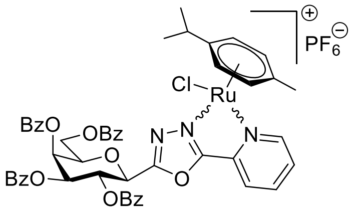	2.44
**Ru-2a**	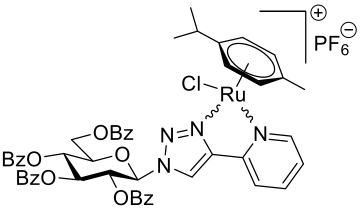	2.85	**Ru-9**	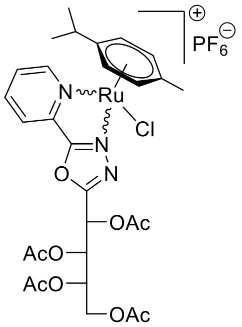	-1.35
**Ru-3a**	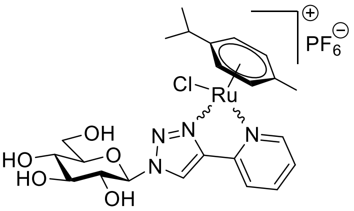	-1.85	**Ru-10**	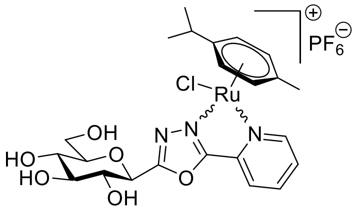	-1.93
**Ru-3b**	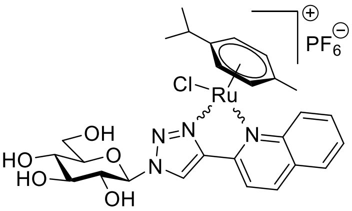	-1.30	**Ru-11**	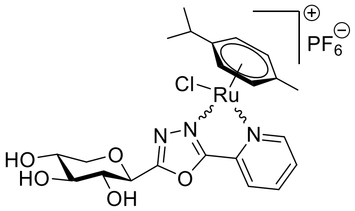	-1.73
**Ru-4**	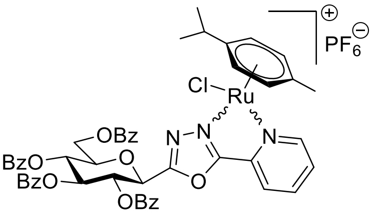	2.41	**Ru-12**	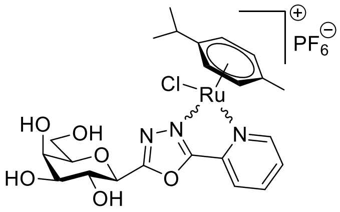	-1.73
**Ru-5**	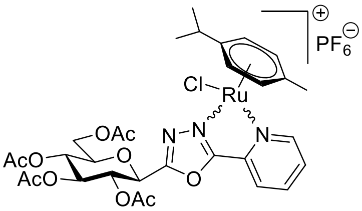	-1.80	**Ru-13**	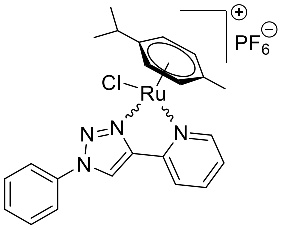	0.44
**Ru-6**	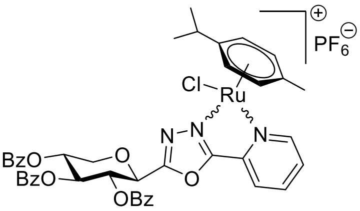	2.04	**Ru-14**	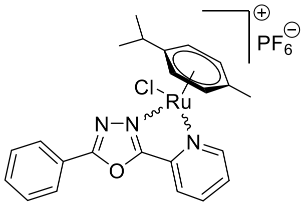	1.63
